# Antibody-Antigen Binding Interface Analysis in the Big Data Era

**DOI:** 10.3389/fmolb.2022.945808

**Published:** 2022-07-14

**Authors:** Pedro B. P. S. Reis, German P. Barletta, Luca Gagliardi, Sara Fortuna, Miguel A. Soler, Walter Rocchia

**Affiliations:** ^1^ CONCEPT Lab, Istituto Italiano di Teconologia, Genova, Italy; ^2^ Bioisi, University of Lisbon, Lisbon, Portugal; ^3^ Universidad Nacional de Quilmes/CONICET, Quilmes, Argentina; ^4^ The Abdus Salam International Centre for Theoretical Physics (ICTP), Trieste, Italy; ^5^ Dipartimento di Scienze Matematiche, Informatiche e Fisiche, Universita’ di Udine, Udine, Italy

**Keywords:** antibody-antigen complex, epitope analysis, paratope analysis, complementarity-determining region (CDR), structure analysis and characterization

## Abstract

Antibodies have become the Swiss Army tool for molecular biology and nanotechnology. Their outstanding ability to specifically recognise molecular antigens allows their use in many different applications from medicine to the industry. Moreover, the improvement of conventional structural biology techniques (e.g., X-ray, NMR) as well as the emergence of new ones (e.g., Cryo-EM), have permitted in the last years a notable increase of resolved antibody-antigen structures. This offers a unique opportunity to perform an exhaustive structural analysis of antibody-antigen interfaces by employing the large amount of data available nowadays. To leverage this factor, different geometric as well as chemical descriptors were evaluated to perform a comprehensive characterization.

## 1 Introduction

Antibodies (Ab) have acquired an unquestionable and unprecedented importance as molecular recognition tools in biotechnology and medicine. Their great versatility has enabled their common use in a broad variety of applications, such as for diagnostic immunoassays, biosensors for target detection, or for therapeutic applications. Indeed, the number of FDA-approved therapeutic antibodies achieved in 2021 one hundred units (Mullard, 2021). The optimization of the Ab manufacturing and the development of new Ab derivatives at a lower cost or with enhanced performance, such as therapeutic antibody fragments and bispecific antibodies, has significantly contributed to the gradual establishment of personalized and precision medicine therapies in hospitals (Grilo and Mantalaris, 2019; Jin et al., 2022). Moreover, the COVID-19 emergency has accelerated the development and use of Abs in new antiviral treatments and passive immunotherapies (Taylor et al., 2021).

This scenario has made more compelling the need for a deeper understanding of the molecular details of antibody-antigen (Ab-Ag) binding. Fortunately, the experimental structural data are helping this process of thorough comprehension. According to the Structural Antibody Database (SabDab) (Dunbar et al., 2013; Schneider et al., 2021), the 2021 increase in the number of experimentally determined Ab-Ag structures reached 66% with respect to the previous year, and 136% with respect to the five preceding years. 4638 Ab-Ag structures have been deposited in the SabDab at time of writing. The availability of such a large structural database with atomic detailed Ab-Ag complexes enables for extensive statistical studies of Ab binding. Capturing the hallmarks of this interaction has a direct impact on structural prediction tools and Ab design approaches. Moreover, the application of statistical inference and machine learning techniques on this data set could also be used to realize new and better predictive tools.

To date, many works have already employed Ab-Ag structural databases to study the features of the antigen (Rubinstein et al., 2008; Sun et al., 2011; Kringelum et al., 2013; Zheng et al., 2015), the antibody (Peng et al., 2014; Kuroda and Gray, 2016; Tsuchiya and Mizuguchi, 2016; Nguyen et al., 2017) or the whole complex (MacCallum et al., 1996; Ramaraj et al., 2012; Kunik and Ofran, 2013; Stave and Lindpaintner, 2013; Dalkas et al., 2014; Wang et al., 2018). Despite the differences in the adopted data sets or in the employed analysis methodologies, a consensus has been reached on some representative features of the Ab-Ag interface, as commented in the Discussion section of this work. Nevertheless, some quantitative disagreement on certain features, such as the size of the Ab-Ag interface or its amino acid composition, can still be found, mainly due to the limited size of the analyzed data sets. To the best of our knowledge, a database of 403 Ab-Ag structures was the largest ever used for this kind of analysis (Nguyen et al., 2017).

In this work, we present a thorough analysis of the main geometric and physico-chemical features that describe the interaction between Ab and Ag in a data set of 1425 Ab-Ag complexes. Moreover, we propose a novel protocol for the identification of hydrophobic clusters at protein-protein interfaces that allowed us to map the polar and hydrophobic interactions occurring at the binding interface and to rationalize the role of highly-represented amino acids in the Ab-Ag complex.

## 2 Methods

### 2.1 The Ab-Ag Data Set

The Ab-Ag complex structures used in this work were extracted from the SabDab (Dunbar et al., 2013) in May 2022. We selected a non-redundant set of 1309 structures with 90% Ab sequence identity and with Ag comprising at least 50 amino acids. Non-proteic antigens, such as short peptides and nucleic acid based Ag, were not included in the selection. Ab Complementarity Determining Regions (CDRs) have been annotated with ANARCI (Dunbar and Deane, 2015) using Chothia numbering scheme. All structures have been stripped of their crystallisation waters as well as ions and other small molecules, when present. Before doing this, however, the information coming from the 597 structures having resolved water molecules was used to estimate the role of water-mediated interactions in Ab-Ag binding. Missing atoms were reconstructed with PDB2PQR (Dolinsky et al., 2007) (49 structures failed). Therefore, the results reported in this work concern the remaining set of 1260 PDB structures from which we extracted 1425 distinct Ab-Ag complexes. This difference is mostly due to multiple Abs binding the same Ag in different regions.

### 2.2 Ab-Ag Interface Definition

We define the Ab-Ag binding interface as the portions of the Solvent Excluded Surface (SES) of Ab and Ag which become buried upon binding. It is worth highlighting that the two individual structures of the Ag and the Ab are rigidly extracted from the structure of the complex and therefore no rearrangement is considered. Analysis of local rearrangement upon binding can for instance be found in Rubinstein et al. (2008). The area of the binding interface, here indicated as A (), can be calculated as the following combination of areas:

$$A\left(\mathrm{interface}\right)=A\left({SES}_{\text{Ab}}\right)+A\left({SES}_{\text{Ag}}\right)-A\left({SES}_{\text{Ab}-\text{Ag}}\right)$$

A(interface) is the sum of the individual buried surface areas of Ab and Ag.

In the literature, the most common definition of surface in this kind of calculations is the Solvent Accessible Surface (SAS), probably because of the easiness of calculating its area. We however think that, while in many cases the numerical differences between the SAS Area and the SES Area are limited (in our experience the latter is around 95% of the former, on average) the most appropriate definition is that of the SES. Both definitions go back to the work of Richards (1977) and consider a spherical probe, representing water, rolling over a given structure of a molecule. Then, the SAS is the locus of the centers of the rolling probe while the SES is the locus of the tangent points between the probe and the protein in the locally convex regions of the protein whilst it is the surface of the spherical probe itself in the concave, or re-entrant, ones. More details can be found in Decherchi et al. (2013) and in Supplementary Figures S1A,B. For the sake of clarity, we note that the SES “touches” the atoms that contact the solvent and therefore is a measure of their solvent exposedness. In this work, each area contribution was estimated with the NanoShaper software (Decherchi and Rocchia, 2013).

Atoms that become buried upon complex formation are defined as *interfacial*. In this work, a residue is considered interfacial if it possesses at least one interfacial atom. Here, we also define the *epitope* as the set of interfacial residues of the Ag, and the *paratope* as the set of those belonging to the Ab. The total buried surface area may not exactly correspond to the sum of the areas of all interfacial residues, since peripheral residues may become only partially buried upon binding. Moreover, as already noted by (Ramaraj et al., 2012; Kuroda and Gray, 2016), only in the case of good packing and therefore high complementarity, one can assume that the interface area of the Ag (i.e., the area of the epitope) and that of the paratope are equal and correspond to half of the buried surface area, A(interface). Finally, this procedure excludes regions potentially involved in water-mediated interactions, which, by definition, occur between atoms that are more than a water molecule away and therefore remain solvent exposed also upon binding.

Based on the previous definitions, and exploiting the list of exposed atoms for a given SES provided by NanoShaper, epitope and paratope can be identified by comparing different surfaces. Given the set of residues (Ab-Ag)_surf_ at the surface of the Ab-Ag complex and the set of residues Ag_surf_ at the surface of the Ag, the epitope can be defined as the portion of residues participating to the SES of the Ag which do not participate to the SES of the complex. In more mathematical terms, referring to the framework of set theory, this corresponds to the relative complement of (Ab-Ag)_surf_ in Ag_surf_:

$$Epitope={Ag}_{\text{surf}}\backslash {\left(\text{Ab}-\text{Ag}\right)}_{\text{surf}}$$ (1)

The same logic can be applied to the paratope:

$$Paratope={Ab}_{\text{surf}}\backslash {\left(\text{Ab}-\text{Ag}\right)}_{\text{surf}}$$ (2)

Recalling that an Ab is composed by two chains, known respectively as light (L) and heavy (H), each comprising three CDRs (conventionally labelled L1-3 and H1-3), the above analysis can be repeated to identify the interface between each of the six CDRs and the Ag. The composition of the SES of the Ab-Ag complex is thus calculated while retaining only one CDR at the time and discarding the rest of the Ab structure, ${\left({CDR}^{i}-\text{Ag}\right)}_{\text{surf}}$ , *i* ∈ {H1, H2, H3, L1, L2, L3}. This leads to six more runs of NanoShaper, one for each CDR-specific epitope $\left({Epitope}_{\text{CDR}}^{i}\right)$ , resulting in six CDR-Ag interfaces.

$${Epitope}_{\text{CDR}}^{i}={Ag}_{\text{surf}}\backslash {\left({CDR}^{i}-\text{Ag}\right)}_{\text{surf}}$$ (3)

The individual ${\left({CDR}^{i}-\text{Ag}\right)}_{\text{surf}}$ sets allow also to determine which epitope residues are concurrently interacting with two CDRs $\left({Epitope}_{\text{Shared}}^{i,j}\right)$ :

$${Epitope}_{\text{Shared}}^{i,j}={Epitope}_{\text{CDR}}^{i}\cap {Epitope}_{\text{CDR}}^{j},\;i\neq j$$ (4)

The union of these sets allows to evaluate the total epitope residues interacting with more than one CDR per Ab-Ag complex:

$${Epitope}_{\text{Shared}}=\underset{i,j\in \left\{H1,H2,H3,L1,L2,L3\right\},\;i\neq j}{⋃}{Epitope}_{\text{Shared}}^{i,j}$$ (5)

Two more NanoShaper runs are performed to characterize the SES relative to the heavy, ${\left({Ab}^{\text{H}}-Ag\right)}_{\text{surf}}$ , and to the light, ${\left({Ab}^{\text{L}}-Ag\right)}_{\text{surf}}$ , Ab chains. Similarly, the epitope residues buried by one Ab chain are defined as

$${Epitope}_{\text{chain}}^{k}={Ag}_{\text{surf}}\backslash {\left({Ab}^{k}-\text{Ag}\right)}_{\text{surf}}\;k\in \left\{H,L\right\}$$ (6)

With this extra information it is possible to pinpoint the residues that are part of the epitope due to cavities formed between the two Ab chains, which we call Epitope_InterChain_.

$${Epitope}_{\text{InterChain}}=Epitope\backslash \left({Epitope}_{\text{chain}}^{\text{H}}\cup {Epitope}_{\text{chain}}^{\text{L}}\right)$$ (7)

Using a similar logic, one can define Epitope_InterCDR_, that is the interfacial residues that are part of the epitope due to cavities formed between two or more CDRs. They can be derived from the comparison of the binding interfaces between the Ag and the CDRs taken individually or taken altogether (CDR^all^ is the structure of all the CDRs of the Ab discarding the remaining part of it).

$${Epitope}_{\text{InterCDR}}={Epitope}_{\text{CDR}}^{\text{all}}\backslash {Epitope}_{\text{CDR}}^{\text{individuals}}$$ (8)

$${Epitope}_{\text{CDR}}^{\text{all}}={Ag}_{\text{surf}}\backslash {\left({CDR}^{\text{all}}-\text{Ag}\right)}_{\text{surf}}$$ (9)

$${Epitope}_{\text{CDR}}^{\mathrm{individuals}}=\underset{i\in \left\{H1,H2,H3,L1,L2,L3\right\}}{⋃}{Epitope}_{\text{CDR}}^{i}$$ (10)

The Ab framework region was also subject to analysis. Each variable domain of the antibody (V_H_, V_L_) is formed by the three CDRs and the framework (Fw), which in principle acts as a scaffold for the CDRs. In fact, although it is accepted that CDRs are chiefly participating to the binding, also the framework can contribute to the paratope. The framework-specific epitope subregion (Epitope_Fw_) can be identified by considering the binding interface of the Ag in contact with only the Ab framework ${\left({Ab}_{\text{Fw}}-\text{Ag}\right)}_{\text{surf}}$ ,

$${Epitope}_{\text{Fw}}={Ag}_{\text{surf}}\backslash {\left({Ab}_{\text{Fw}}-\text{Ag}\right)}_{\text{surf}}$$ (11)

Finally, from the set difference between the entire epitope and the union of all the CDR individual epitopes one can retrieve (Epitope_ExtraCDR_), the set of epitope residues that are not directly buried by any CDR, taken individually.

$${Epitope}_{\text{ExtraCDR}}=Epitope\backslash {Epitope}_{\text{CDR}}^{\mathrm{individuals}}$$ (12)

Epitope_ExtraCDR_ includes Epitope_InterCDR_, Epitope_InterChain_ and Epitope_Fw_.

A graphical sketch of these definitions is provided in Supplementary Figure S1C.

### 2.3 Characterization of the Binding Surfaces

For our analysis of binding surfaces, we exploited the ability of NanoShaper to provide a triangulation of the SES, in the form of a mesh, as well as a list of exposed atoms for a given molecular system.

**FIGURE 1:**
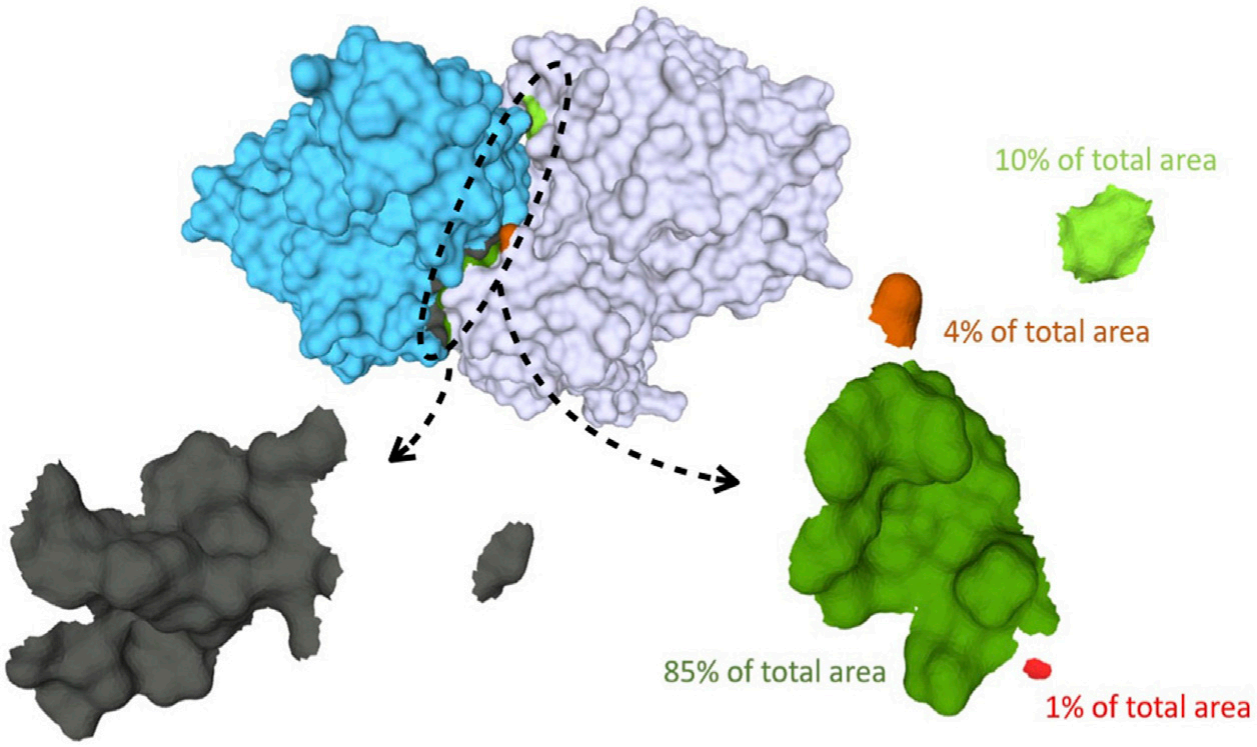
Binding surfaces identification and separation into connected components for Human Hedgehog acyltransferase in complex with two Fab antibody fragments (pdb code: 7MHY). In cyan the surface of the antibody fragment; in white, the antigen. The paratope, shown in the left bottom, is in gray while the different connected components of the epitope surface are colored in green and red, respectively. On the right: zoom of the epitope surface patches. Each color indicates a distinct connected component of the epitope. Components that are a fraction below 5% of the total contact area are discarded, yielding in this case two residual components.

We first identify the interfacial atoms of epitope and paratope. Then, we determine the mesh vertices of epitope and paratope that are closest to them. To avoid checking for each system *n*_*b*_ × *n*_*v*_ pair distances, being *n*_*b*_ the number of interfacial atoms and *n*_*v*_ that of all the vertices in the triangulation, we make use of the list of closest-atoms-per-vertex, also returned by NanoShaper. However, a direct comparison between this list and the interfacial atoms would only lead to the generation of unreasonably fragmented surfaces. We therefore extend the set of vertices from those closest to interfacial atoms to those closest to their parent residues (Eqs 1, 2). Finally, we perform a pruning and keep only the vertices located within 4 Å from any interfacial atom of the opposite interface (e.g. when building the epitope mesh, the pruning takes into account the paratope atoms and vice versa). This step is necessary to avoid including vertices unreasonably far from the contact region (e.g. in regions where part of a residue is oriented away from the contact region). By having selected only the vertices in the vicinity of the interfacial residues, the number of pair distances to be calculated is greatly reduced. As illustrated by the top panel of Figure 1, this procedure allows to define epitope and paratope meshes, distinct from the rest of the SES. As already mentioned, we note that the area of epitope and paratope surfaces calculated in this way does not precisely correspond to half of the buried surface area, due to both the extension from interfacial atoms to their parent residues and to the possible presence of small cavities at the interface.

Thereafter, the epitope mesh is analyzed to establish the number of connected components (distinct surface patches) and their relative contribution to the whole epitope contact area. This analysis, performed *via* the *pymeshlab* Python library Muntoni and Cignoni (2021)– an interface of the popular *MeshLab* software Cignoni et al. (2008), allows to split the epitope surface in distinct connected components and measure their area. To avoid spurious contribution from small disconnected patches we discard all components whose area is below 5% of the total epitope area. This is illustrated in the bottom part of Figure 1.

### 2.4 Epitope Residue Exposedness

In order to quantify the preference of the epitope location for the most exposed regions of the antigen, we consider the SES of the antigen calculated with three different probe radius values: *R*_*p*1_ = 1.4 Å, *R*_*p*2_ = 9 Å and *R*_*p*3_ = 100 Å. The first SES definition is the usual one, that considers all the regions accessible by the solvent. *R*_*p*2_ approximates the size of Ab CDRs (Rubinstein et al., 2008). *R*_*p*3_ is an arbitrarily large value leading to a SES which approaches the *convex hull* limit. The convex hull of a set of points (here generalized to atoms) is the smallest convex set containing them. In this limit, only the most protrusive atoms in the solvent are still exposed (since the very large probe sphere is unable to seep into any groove).

We want to assess the portions of the epitope that reside in the regions at different exposedness. We therefore calculate the fraction of residues which are exclusively exposed at a given probe radius, with respect to the total number of residues of the epitope. Note that being $E_{R}$ the set of exposed residues at a given probe radius R, $E_{R=100}\subset E_{R=9}\subset E_{R=1.4}$ .

### 2.5 p*K*_a_ Shifts Calculation

The p*K*_a_ values of the titratable residues at the Ab-Ag interface were estimated with PypKa (Reis et al., 2020) by using default parameters. PypKa was successfully run on 875 of the 1425 Ab-Ag pairs. The calculations were performed with an ionic strength of 0.1 M and the dielectric constant for the protein was set to 15. To obtain p*K*_a_ shifts with respect to their values in water, experimental p*K*_a_ values for all the amino acid residues considered were taken from (Thurlkill et al., 2006) and (Grimsley et al., 2009). These values were measured for each amino acid in capped alanine-based pentapeptides in which the central residue is titratable, thus including the average effect of protein backbone. The reported p*K*_a_ shifts reflect the environment change caused by the binding.

### 2.6 Hydrophobic Interaction Analysis

**FIGURE 2:**
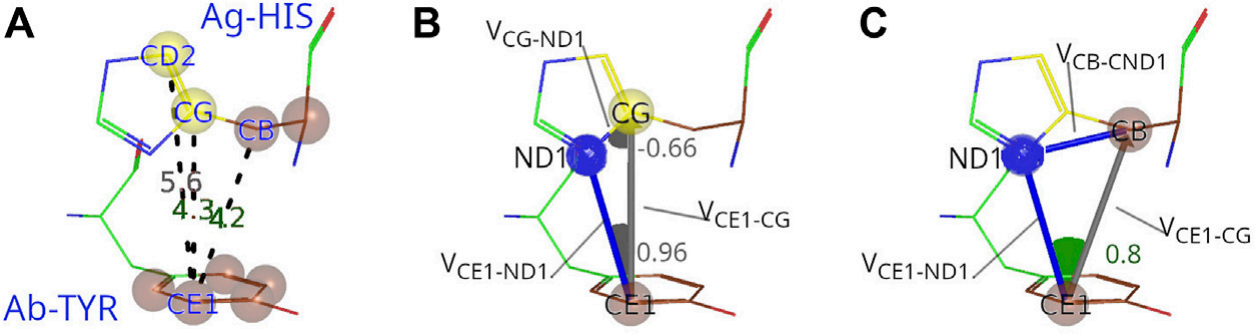
Procedure to characterize C-C interactions between Ag and Ab. **(A)** The example of interacting carbon pair from PDB ID: 4CMH shows that the distances between CE1 atom from Tyr92 (paratope) and CB and CG atoms from His79 (epitope) are within the threshold (5 Å), but not between CE1 and CD2. **(B)** Evaluation of the potential shielding between CE1 and CG. The first dot product between ${\vec{V}}_{\mathrm{CE1-CG}}$ and ${\vec{V}}_{\mathrm{CE1-ND1}}$ exceeds the threshold (0.85) and indicates that the ND1 atom from His79 is either between the C atoms, or behind CG. The second dot product between ${\vec{V}}_{\mathrm{CE1-CG}}$ and ${\vec{V}}_{CG-ND1}$ confirms the shielding as its value is below the threshold (−0.2). **(C)** Evaluation of the potential shielding between CE1 and CB. The first dot product value between ${\vec{V}}_{CG-CE2}$ and ${\vec{V}}_{CG-ND1}$ below the threshold (0.85) already proves that CE1-CB interaction is cleared. In all figures, carbon atoms accepted for the hydrophobic cluster are shown in brown, while rejected C atoms are in yellow and nitrogen in blue.

The hydrophobic interaction between Ab and Ag was characterized by a graph-based clustering of the positions of the interacting carbon (C) atoms of the paratope and of the epitope. A pair of interfacial C atoms that are close enough to interact and, at the same time, are not shielded by any surrounding polar atoms are defined as interacting C atoms. To determine which C atoms from the interface belong to a hydrophobic cluster, the following steps were carried out:

1. The atomic pairwise distances between C atoms of the epitope and the paratope are calculated using the MDTraj library (McGibbon et al. (2015)). C atoms belonging to each binding partner that are closer than 5 Å are annotated as potentially interacting.
2. For each stored C-C interaction, a putative shielding from oxygen or nitrogen atoms due to their location between both Cs, is evaluated. For the evaluation: a triangle is formed between the C pair and each of the nearby polar atoms. Then, the inner angles of these triangles are evaluated to determine if the polar atom is located between the two. This is done by calculating the dot products of the vectors $\vec{V}$ that join each two of the three atoms. In particular, a first dot product is evaluated between the vectors that go from the paratope C to the epitope C atom and from the former to the polar atom. Dot product values lower than a threshold of 0.85 indicate that the C atom pair is cleared. Otherwise, a polar atom is either between both C atoms or “behind” the epitope C atom. Thus, a second dot product is calculated to resolve the ambiguity, namely the product of the vector connecting the paratope C to the epitope C, and the vector connecting the latter to the polar atom. Dot product values lower than −0.2 indicate that the polar atom is actually shielding the C atoms. A detailed graphical example of steps 1–2 is showed in Figure 2.
3. If the C pair is cleared from any shielding, it is added to a graph where the C atoms are represented as nodes and their interaction as edges. If one of the C atoms was already present in the graph then only the new C is added as a node and an edge is formed between the new C and the previous one. The Networkx library (Hagberg et al. (2008)) was used to construct the graph and extract its connected components.
4. From this graph, connected components are extracted. A connected component in a graph is a set of nodes that are mutually reachable by traversing their connecting edges. In our graph model, where C atoms are nodes and interactions between the C atoms are edges, each connected component represents a hydrophobic cluster.

It is worth pointing out that the results of this analysis proved to be quite insensitive to variations in the chosen C-C distance threshold, in the 4–5 Å range, and also with respect to the considered angle thresholds, chosen heuristically.

### 2.7 Polar Interaction Analysis

Polar interactions between heavy atoms of Ab and Ag were evaluated by employing HBPLUS (McDonald and Thornton, 1994) with its default 3.9 Å distance threshold. The output from HBPLUS was later parsed to differentiate standard H-bonds from salt bridges. Salt bridges were defined as those between the side-chain cationic nitrogen atom from Arginine or Lysine and the side-chain anionic oxygen atom from Glutamate or Aspartate. When water molecules were present in the PDB structure, hydrogen bond interactions between water molecules and Ab or Ag were also computed with HBPLUS. Water-mediated interactions were identified by evaluating crystallographic water molecules simultaneously forming hydrogen bonds with polar atoms of the Ag and the Ab.

### 2.8 Ring Interactions

*π*-*π*, cation-*π* and anion-*π* interactions were evaluated as representative interactions in which aromatic rings are involved. We identified a *π*-*π* interaction, in which two aromatic rings are interacting in a parallel conformation, when the absolute value of the dot product of their normal vectors is above 0.85 and their centers of mass are closer than 5 Å (*see* Supplementary Figure S2A). *π*-ion interactions satisfied a similar set of criteria: if the ion and the center of mass of the aromatic ring were closer than 5 Å, and the vector joining them had a dot product against the normal vector of the ring with an absolute value above 0.70 (meaning the ion is facing the surface of the ring), then such ion-ring pair was classified as interacting (Supplementary Figure S2B).

## 3 Results

### 3.1 Antibody Analysis: The Paratope

**FIGURE 3:**
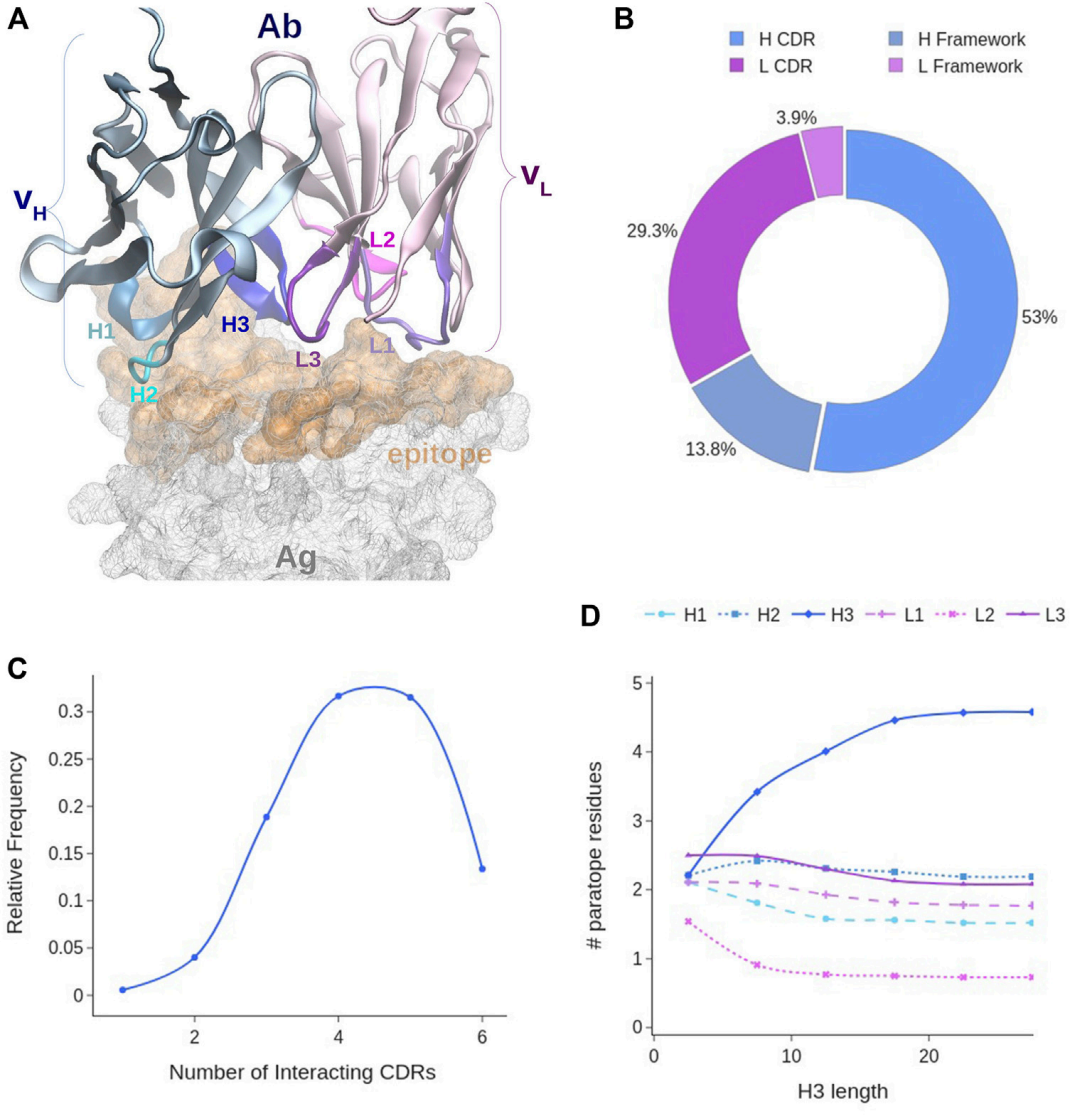
Paratope analysis. **(A)** Covox-269 Fab interacting with the receptor binding domain of SARS-Cov-2 spike protein (PDB code: 7NEH) as representative structure of an Ab-Ag complex. **(B)** Distribution of paratope residues according to Ab chain type and CDR presence. **(C)** Distribution of CDRs in the paratope. **(D)** Distribution of paratope residues given the number of H3 residues. Color code: light chain elements (magenta), heavy chain elements (cyan).

The paratope is the region of the Ab surface that is directly interacting with the corresponding antigen. It usually includes portions of both the light (L) and heavy (H) Ab chains. In particular, the paratope is often assumed to include all of the six CDRs, which are located in the variable domains V_*H*_ and V_*L*_ of the respective chains (*see* Figure 3A).

The six CDRs are optimized by our immune system to generate high affinity paratopes for the molecular recognition of a specific target.

Our analysis of the paratopes showed that 95% of them include both H and L chains, with the latter contributing to a lesser extent. Indeed, ∼67% of the paratope residues belong to the H chain (*see* Figure 3B). On average, the paratope contains 15.6 ± 4.7 residues, 10 of which belonging to the H chain and 5 to the L chain (Supplementary Figure S3A). In the majority of paratopes (85%), we found the co-participation of framework (Fw) residues. This is an interesting aspect as their main role is to act as a scaffold for the CDRs (*see* Figure 3B). In fact, their contribution, while generally less important than that of the CDRs, can account for as much as 30% of the paratope. However, in general, CDR residues still participate up to around the 80% of the paratope, and a paratope with less than 70% of CDR participation was very rarely observed. Over the whole dataset, the CDRs contribute on average with 12 residues.

The contribution of each CDR to the paratope was calculated over the whole dataset. On average either 4 or 5 CDRs are interacting with the target (Figure 3C). Observing less than three CDRs participating in the binding is unlikely and may correlate with poor binding affinity. We found that the most frequently participating CDR is H3, accounting for about one third of all the paratope residues (Supplementary Figure S3B). Conversely, only 6% of the paratope residues are from L2, making it the least participating one. On average, H3 and L3 have the highest participation: four residues from H3 and 2 from L3, while L2 and H1 have the lowest contribution (Supplementary Figure S3C). Since previous works studied the influence of H3 length to the binding conformation of the paratope, we analyzed this possible correlation. Although a typical H3 loop contains on average 13 residues, there is a wide range of observed lengths, with 68% of H3s having between 8 and 17 residues (Supplementary Figure S3D). Our analysis showed indeed a negative correlation between H3 length and the participation of other CDRs to the paratope for lengths between 1 and 10 residues (Figure 3D). However, above this range of lengths, the size of H3 only minimally affects the binding of other CDRs. Indeed, when H3 contains more than 10 residues, it stops influencing the amount of other CDRs’ residues interacting with the epitope.

**FIGURE 4:**
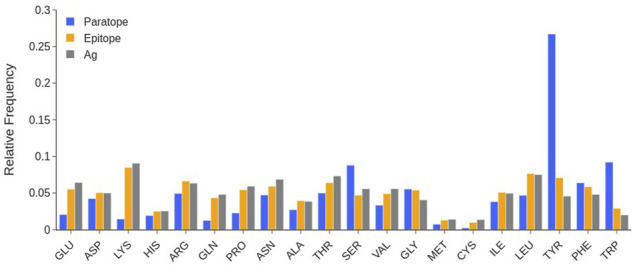
Comparison between the amino acid composition of paratope (blue), epitope (orange), and the entire SES of the antigen (gray). Individual structures are taken from that of the complex, by rigid removal. Data calculated over the SabDab database as of May 2022 (1425 structures).

The analysis of the amino acid composition of the paratopes over the whole database in Figure 4 shows that at least 25% of the paratope residues are Tyrosines (Tyr), followed by the ∼10% being Serines (Ser) and Tryptophanes (Trp).

Other small sized residues such as Glycines (Gly), Threonines (Thr) or Asparagines (Asn) are also quite frequent in the paratope, while Glutamine (Gln) has a low occurrence. As per charged residues, there is a clear preference for Aspartates (Asp) and Arginines (Arg) over Lysines (Lys) and Glutamates (Glu). Other amino acids such as Cysteines (Cys), Histidines (His), Methionines (Met) and Prolines (Pro) are very scarcely represented.

### 3.2 Antigen Analysis: The Epitope

**FIGURE 5:**
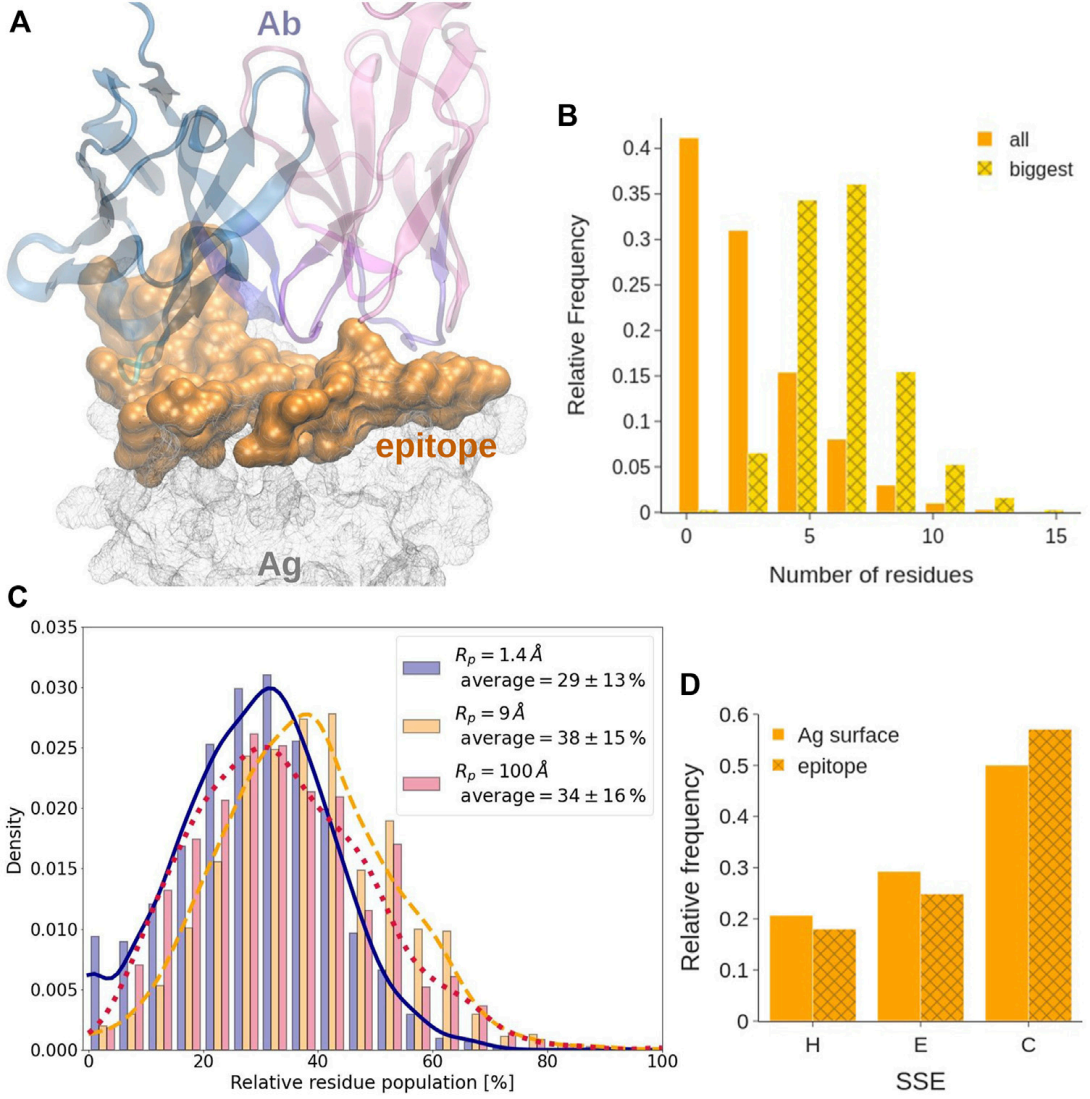
Epitope analysis. **(A)** Covox-269 Fab interacting with the receptor binding domain of SARS-Cov-2 spike protein (PDB code: 7NEH) as representative structure of Ab-Ag complex. The antigen is represented as a solid surface. Color code: antibody heavy/light chains (cyan/magenta), epitope (orange). **(B)** Length, expressed in residue number, distribution in all sequential epitope stretches and in the longest one. **(C)** Distribution of the fraction of residues at the epitope exclusively exposed at each considered probe radius. **(D)** Distribution of the secondary structure of the epitope. The different secondary structure elements were grouped in helix (H), strand (E), and loops (C).

The epitope is the region of the Ag surface that is directly interacting with the Ab (*see* Figure 5A). The structural analysis of the antigens in our database shows that on average the epitope contains 14.6 ± 4.9 residues, thus it is similar in size to the paratope. Epitopes with less than six residues or more than 25 are rarely observed (Supplementary Figure S4A).

#### Epitope Surface Organization

We find that, in most of the considered systems, the epitope surface is constituted by a single connected component, namely a patch. Indeed, only about 25.5% of epitope surfaces consist of more than a single patch. Out of them, about 83% are constituted by just two patches. We also find that in most epitopes with multiple patches, the largest patch covers about 80% of the total epitope area. Therefore, even when the epitope is fragmented, the biggest component is still mostly responsible for the binding.

#### Epitope Linearity

Consistently with what observed previously, *see* for example Haste Andersen et al. (2006) and references therein, epitopes comprising only one linear amino acid sequence, often called sequential or linear epitopes, are rarely observed. Indeed, epitopes often are conformational, i.e., consisting of portions of the Ag that are discontinuous in sequence and become spatially close only upon folding. Analysing how many linear segments they contain and how prominent they are has a significant interest from the biotechnological standpoint in the quest for identification of better and more specific binders. This analysis can be performed by evaluating the number and length of the continuous sequence stretches contained in an epitope. With a tolerance of one gap in the sequence, 80% of epitopes contains between three and eight stretches (Supplementary Figure S4B). Their length follows a power law distribution that approaches 0 at around 10 residues, indicating that more than 70% of stretches contains less than three residues. However, if only the longest stretch of each epitope is considered, they are observed to follow a normal length distribution centered at five to seven residues (*see* Figure 5B).

#### Epitope Exposedness

It has been suggested that epitope residues may be located in more exposed regions as compared to the remaining antigen surface (Novotny et al., 1986; Rubinstein et al., 2008). When considering the degree of exposedness of epitope residues as defined in the Methods, we find that, on average, less than 29% of the epitope is located in the less exposed part of the SES of the antigen, where a probe of *R* = 9 Å could not get. More in detail, ∼38% of the epitope is located in the region of intermediate exposedness, while ∼34% corresponds to residues in the most exposed patches. The distribution of the residue degree of exposedness is summarized in Figure 5C. This is consistent with the fact that epitopes are mostly located in regions accessible to CDRs.

#### Epitope Secondary Structure

According to our secondary structure analysis, performed using the DSSP tool (Kabsch and Sander, 1983), and reported in Figure 5D and in Supplementary Figures S17–S19, epitopes have significantly less regular secondary structure than the rest of the antigens they belong to. Indeed, by grouping and comparing different secondary structural elements, epitopes are shown to contain less helices and strands, with respect to the entire antigens, while they are enriched in coils. Statistical analysis of the distributions of the different structural elements was conducted at the 0.1% of significance with the two-sample Kolmogorov-Smirnov test, confirming our hypothesis. This might correlate with a higher flexibility of these regions, as already observed by Westhof et al. (1984). Detailed statistics on the secondary structure elements is provided as Supplementary Data (dssp_epitope.csv and dssp_surface.csv).

#### Epitope Composition

The evaluation of the epitope amino acid composition in Figure 4 shows that, in general, there are only slight differences with respect to the composition of the remainder of the antigen surfaces, at least in the considered data set. We however observe that Tyr and Trp residues have a larger number of occurrences in epitopes. Charged residues are also slightly more frequent in protein surfaces than in the epitope. The occurrence of long-chain polar residues Asn and Gln is slightly less pronounced in epitopes than in the antigen surfaces, while Ser residue is depleted.

### 3.3 Ab-Ag Interaction Analysis

#### Ab-Ag Surface Characterization

Our evaluation of the size of Ab-Ag interfaces by computing the buried surface area amounts to (1068 ± 314) Å^2^. This result is comparable to what obtained in the literature (Rubinstein et al., 2008; Sun et al., 2011; Ramaraj et al., 2012), also considering that our definition is SES based rather than SAS based, the former being on average 5% smaller than the latter, on average (data not shown).

#### Hydrophobic Interactions

**FIGURE 6:**
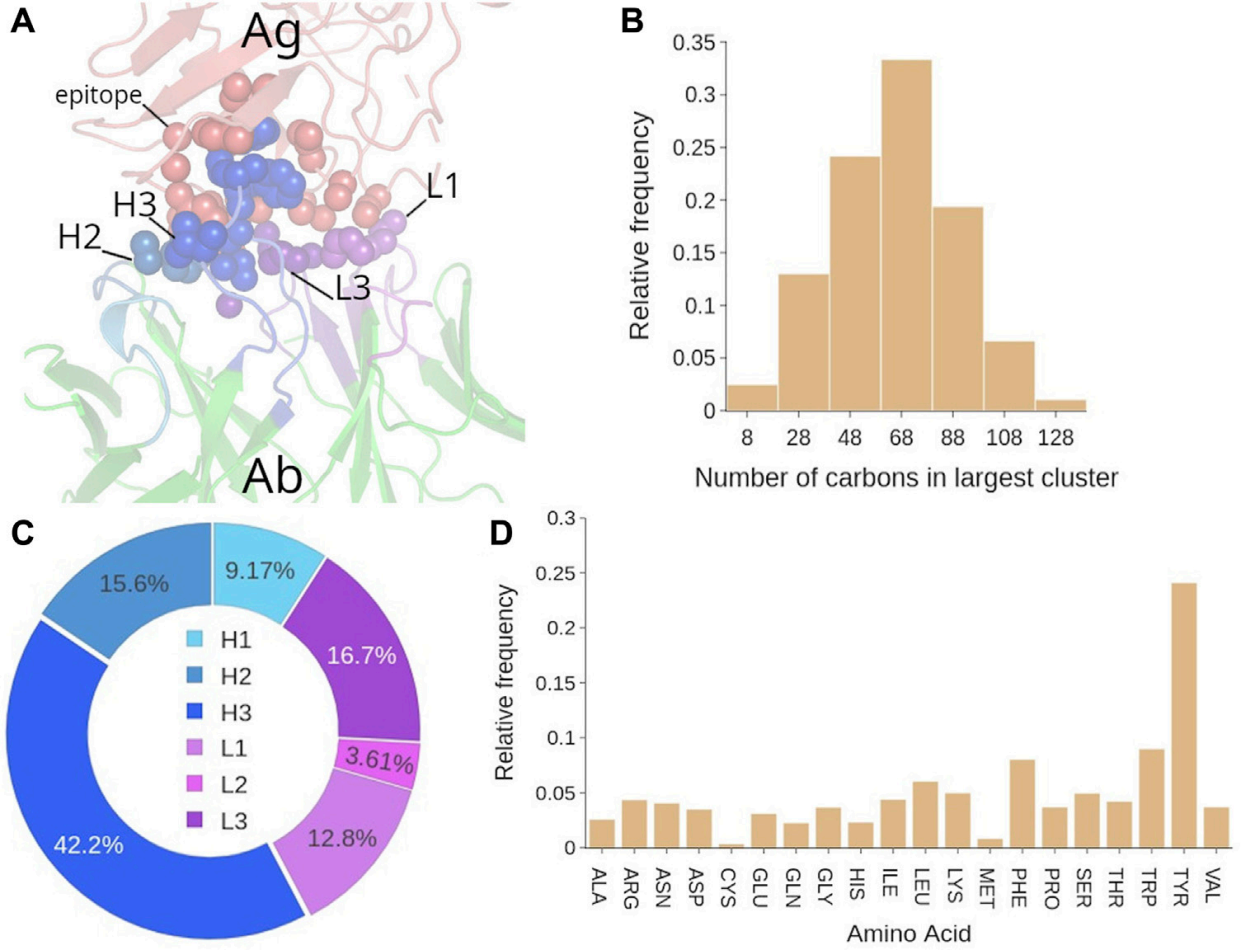
Analysis of the hydrophobic interactions in Ab-Ag complexes. **(A)** Representative structure of the hydrophobic cluster in the Ab-Ag interface. **(B)** Number of C atoms that participate in the biggest hydrophobic cluster. **(C)** Participation (in %) of each CDR to the biggest hydrophobic cluster. **(D)** Amino acid contribution in the biggest hydrophobic cluster, computed by counting the number of carbon atoms contributed by that amino acid type to the cluster.

Hydrophobic interactions were evaluated by identifying the clusters of C atoms occurring at the binding interface (*see* Methods). The number of hydrophobic clusters is on average 2 ± 1, in which one to two clusters account for almost 85% of the sample (*see* Supplementary Figure S5A). The biggest hydrophobic cluster in each Ab-Ag interface has on average 65 ± 24 atoms (*see* Figure 6A as representative structure).

In fact, the distribution of cluster size is broad since in the 75% of the confidence region we can find clusters from 40 to 100 carbon atoms (*see* Figure 6B). Regarding the size of secondary clusters, over 65% of the second biggest hydrophobic clusters are less than one third in size of their respective lead clusters (*see* Supplementary Figure S5B). This meaningful size difference may indicate that the leading hydrophobic cluster has in general a major role in the Ab-Ag binding, and for simplicity we can focus our analysis only on this one. In terms of residues, these C atoms belong to 21 ± 7 residues (Supplementary Figure S5C). Also, we find that in 73% of the Ab-Ag complexes, 2–4 CDRs participate to the biggest hydrophobic cluster (average value of 3 ± 1) (Supplementary Figure S5D), especially H3, (42% of participating residues belong to H3, *see* Figure 6C). The other CDRs included in the biggest hydrophobic cluster are L3 and H2, i.e., near H3. The evaluation of appearance of CDR pairs in the biggest cluster confirmed that H3-H2, H3-L3 and H3-L1 are the top three CDR pairs most commonly found in the biggest cluster (Supplementary Figure S6).

Structure-wise, a clear preference for coils was observed in the epitope residues of the biggest hydrophobic cluster (Supplementary Figure S7A). This result is not surprising considering the average profile of the epitope, enriched in coil structures (Figure 5D). Nevertheless, we can conclude that the most flexible structures of the epitope have a major contribution to the hydrophobic interaction. The amino acid contribution to the biggest hydrophobic cluster was first evaluated by counting the number of carbon atoms contributed by that amino acid type to the cluster (Figure 6D). This analysis showed that Tyr is clearly over-represented, followed by the aromatic residues Trp and Phe. However, when the composition is evaluated in terms of amino acid frequency, that is, avoiding multiple counting due to C atoms belonging to the same residue, then other amino acids of smaller size such as Ser, Gly and Thr emerge (Supplementary Figure S8). This is in line with the average composition of epitope and paratope.

#### Electrostatic Interactions

**FIGURE 7:**
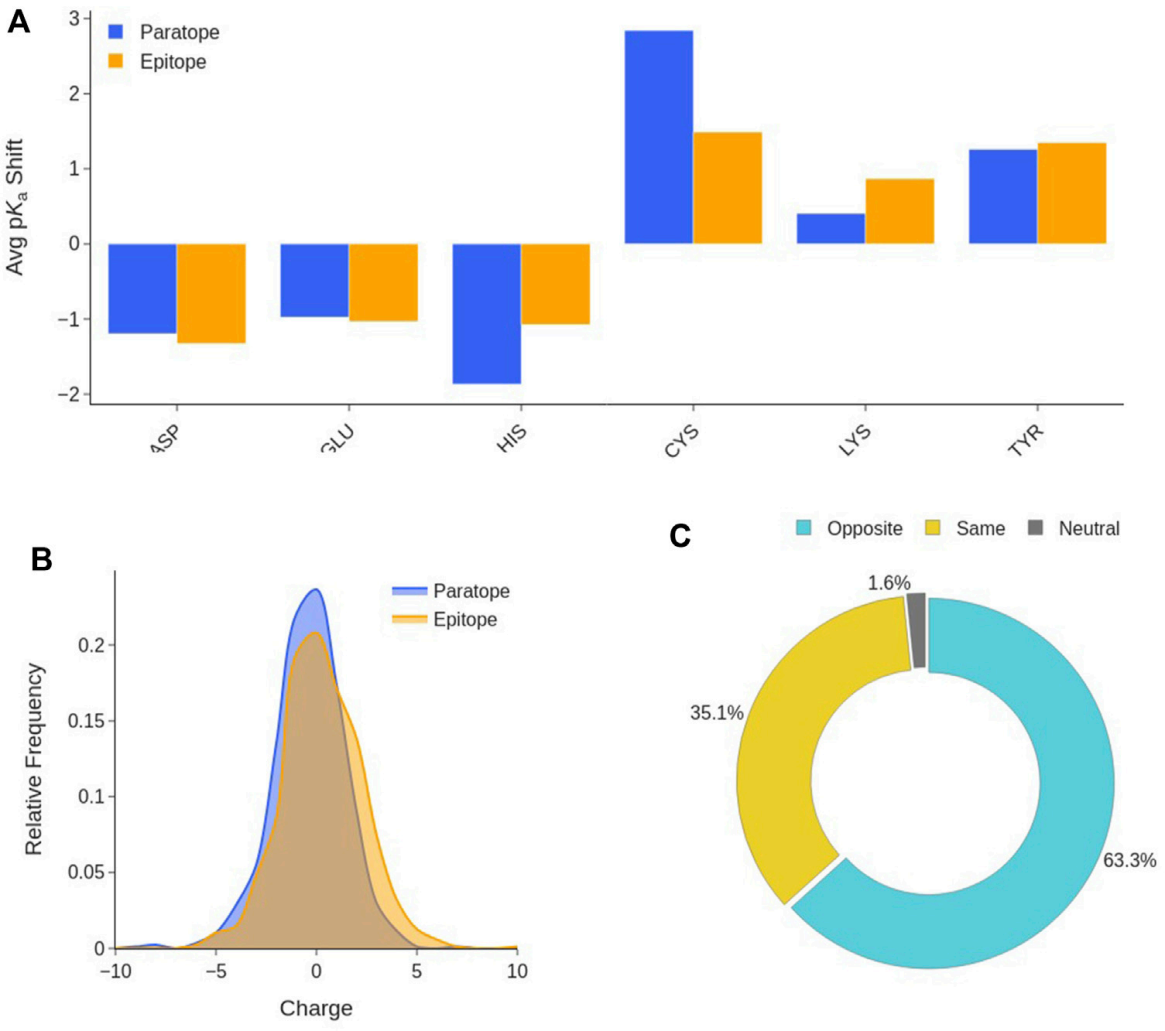
Interface titratable residues characterization. **(A)** Average p*K*_a_ shift of titratable residues at the Ab-Ag interface with respect to their value in water. **(B)** Distribution of the net charge in the paratope and epitope at pH 7.2. **(C)** Ab-Ag interface charge complementary composition. Distinctions are made among interfaces in which paratope and epitope have the same charge, opposite charges, and when one partner is neutral.

Titratable amino acids make up for more than one third of the paratope and epitope residues (Supplementary Figure S9). On average, paratopes and epitopes have 7.7 ± 3.3 (≈41%) and 6.4 ± 3.2 (≈36%) titratable residues, respectively. In order to appropriately estimate the charge state of epitopes and paratopes, we performed p*K*_a_ calculations of the residues that can titrate in the physiological pH range: Asp, Glu, His, Cys, Lys and Tyr. Our results in Figure 7A show that residues titrating in the acidic region, on average, experience a shift to lower values compared to water, while those titrating in the basic region shift to higher values.

This means that, at physiological pH, all titratable amino acids tend to the same protonation state as that preferred in water. This trend is observable in both the paratope and the epitope. This is the consequence of the new environment and interactions established at the Ab-Ag interface. Desolvation favours neutral forms, however, H-bonds can shift a residue in either direction depending on whether it is anionic or cationic and behaving as a hydrogen donor or acceptor. The neutral forms of His, Cys and Tyr are preferred at the interface. Thus, Asp, Glu, Lys and Arg will be sources of charge, likely stabilized by H-bonding and other polar interactions, in which the anionic residues prefer to behave as hydrogen acceptors, and the cationic as hydrogen donors. Subsequently, the net charge borne by the two interfaces is evaluated at pH 7.2. As shown in Figure 7B, neither the paratope nor the epitope have a clear preference for a net charge sign, absolute net charges larger than 5 are rare as both paratopes and epitopes display an average charge of -0.4 ± 1.8 and 0.3 ± 2.0, respectively. On the other hand, the study of the net charge complementarity between paratope and epitope shows that in almost two thirds of the interfaces, paratope and epitope bear opposite net charges (Figure 7C). From the distribution of the product between epitope and paratope net charges (Supplementary Figure S10), one can confirm the two previous analyses at once, as interfaces with nearly neutral charges are the preferred, while interfaces bearing opposite charge are more common than those bearing a net charge of the same sign. These results support the hypothesis that paratope and epitope are electrostatically complementary, and that electrostatic interactions may serve as anchor points to stabilize binding.

**FIGURE 8:**
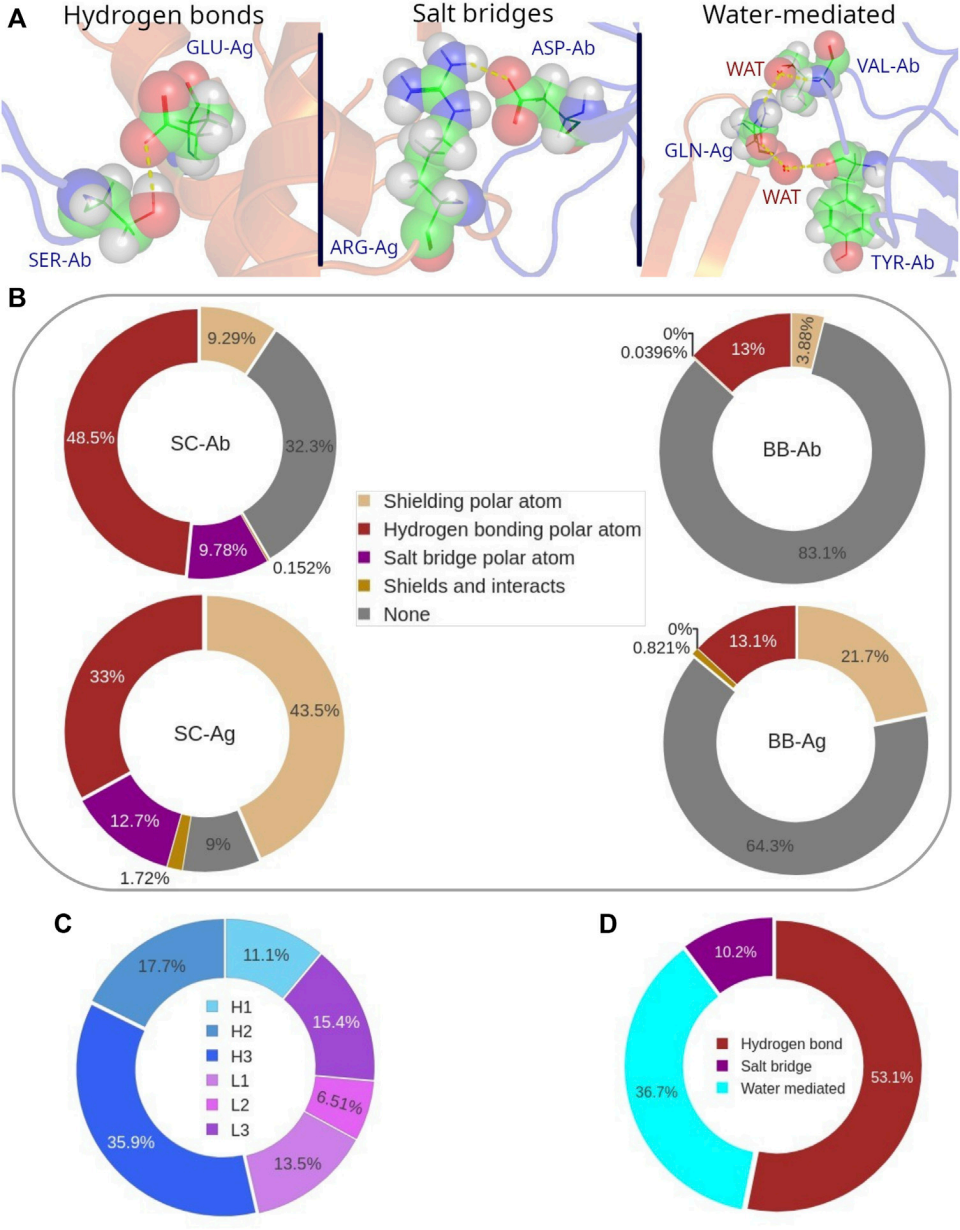
**(A)** Graphical representation of the different types of polar interactions. **(B)** Distribution of the different roles of polar atoms at the Ab-Ag interface. Participation (in %) of the polar atoms in each CDR to the polar bonds. **(C)** Participation of each CDR in polar interactions. **(D)** Distribution probability of H-bonds, salt bridges and water-mediated interactions to all polar bonds.

Polar atoms play an important role at the interface. They actually participate in the strongest non-bonding interactions: hydrogen-bonds (H-bonds), salt bridges, and water-mediated interactions (Figure 8A). For a deeper analysis of the role of the polar atoms in binding, we considered first those belonging both to backbone and side-chain in epitopes and paratopes and classified them according to whether they were performing a polar interaction, (discriminating between H-bonds and salt bridges), shielding some hydrophobic interaction, the combination of the two, and no action. In general, around 48% of polar atoms located in the residue side-chains of paratope are forming H-bonds while 10% of them are participating in a salt bridge. In the epitope, these figures turn to be 33%, and 13%, respectively (Figure 8B). In particular, we find that the proportion of H-bonds over salt bridges among the polar bonds that occur in the interface is over 6 to 1, when we don’t limit our analysis to the structures that contain water molecules (Supplementary Figure S11).

Conversely, the backbone polar atoms have a minor participation to H-bonds, since around 13% of them are forming this type of interaction, for both epitope and paratope. Overall, the number of H-bonds in an Ab-Ag interface is on average 7 ± 3, most of them formed between the side-chains of Ab and Ag.

More interestingly, over 30% of the paratope side chains as well as over 80% of the paratope backbone polar atoms are not involved in any interaction. A quite different behavior is observed in the epitope, where polar atoms have a larger role in shielding carbon atoms. This is particularly evident in the epitope side-chain region, as more than 40% of side-chain polar atoms are performing this role. It follows that most polar atoms that disrupt the hydrophobic cluster are penalizing the Ab-Ag interaction since they are not forming any type of polar bond at the interface.

The analysis of the distribution of the polar bonds, considering H-bonds and salt bridges, in the paratope shows that CDR H3 contains the highest number of polar bonds, followed by H2, L3 and L1 (Figure 8C). This result follows a similar trend as we previously observed for the distribution of the paratope residues that participate in the hydrophobic interaction. However, the contribution of the CDR H3 residues to the polar bonds decreases in parallel to the proportional increase of all other CDRs, except for L3, so that the distribution of the polar bonds is certainly broader in the paratope. Regarding the epitope, the analysis of the secondary structure of the residues that participate in polar bonds shows that polar bonds have also a clear preference for flexible coil structures in the epitope, following the same behavior of the hydrophobic interactions (Supplementary Figure S7B). This result confirms the preference of the Ab paratope to bind the less structured regions of the epitope.

The evaluation of *water-mediated interactions* was limited by the availability of Ab-Ag structures resolved with resolution high enough to pinpoint water molecules. Nevertheless, a SabDab subset of 597 Ab-Ag structures including water molecules was found. An equivalent analysis of the role of polar atoms in this database showed that their average participation to water-mediated bonds ranges 11–13% (Supplementary Figure S12). This percentage is mainly subtracted from the non-interacting polar atoms, while other roles keep their probability values. The distribution of polar bonds now considering water-mediated is 53% H-bonds, 37% water-mediated and 10% salt bridges (*see* Figure 8D).

#### CDR Co-participation in Binding

**FIGURE 9:**
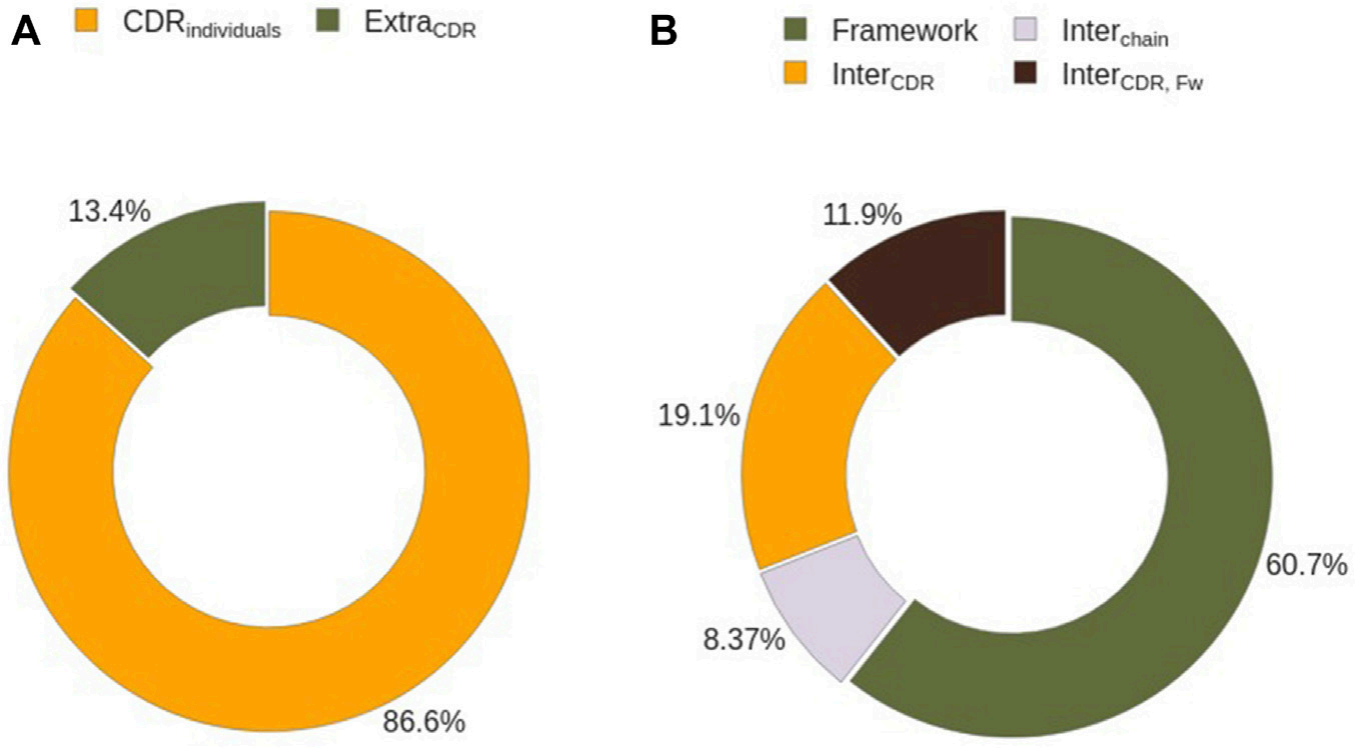
Epitope residues characterization by their interacting partner. **(A)** Identification of epitope residues interacting with single CDRs. **(B)** Sub-classification of epitope residues not interacting with CDRs on an individual basis.

We analyzed how many CDRs co-participate in binding the same region to understand whether every CDR can be studied individually. First, we assessed the number of epitope residues that can not be identified solely by the presence of individual CDRs (Epitope_ExtraCDR_), defined as the differences in the solvent exposed residues of the Ag in the absence and in the presence of CDRs (*see* Methods). 87% of residues belong to ${Epitope}_{\text{CDR}}^{\text{individuals}}$ , and only 13% to Epitope_ExtraCDR_, corresponding to those that are not directly buried by any CDR when considering individual CDRs (Figure 9A). In other words, in 91% of all epitopes one is likely to find four or less Epitope_ExtraCDR_ residues (Supplementary Figure S13A). Further, in more than one fourth of epitopes there are no Epitope_ExtraCDR_ residues, meaning that in these complexes one is able to identify the epitope solely from the union of Ag residues that interact with each single CDR.

A closer inspection of the composition of Epitope_ExtraCDR_ shows that 61% of them correspond to the interaction of the epitope with the Ab Framework (Figure 9B). Despite being the biggest contributor Epitope_ExtraCDR_, this term is zero in half of the analysed complexes. The remaining 49% are divided between the residues captured by the cavities formed by CDRs (19%, Epitope_InterCDR_), by CDRs and the Ab framework (12%, Epitope_InterCDR,FW_), and by the Ab chains (8%, Epitope_InterChain_). This cavity term between Ab chains is the least represented term in Epitope_ExtraCDR_, and 83% of the analyzed epitopes contain no Epitope_InterChain_ (Supplementary Figure S13B).

With the same data, we identified Epitope_Shared_ residues, which bind simultaneously to two or more CDRs (Supplementary Figure S13C). In ∼74% of epitopes we have observed two or less Epitope_Shared_ residues, which amount to less than one residue (0.3 ± 0.2 residues) being solvent excluded by more than one CDR, most of which are shared between H3 and L3 (Supplementary Figure S13D).

Considering the previously raised points, one might argue that performing a CDR-centric classification of the epitope residues is a reasonably good approximation. However, we should also take into account the hydrophobic cluster analysis, which shows that the co-participation of different CDRs to the same hydrophobic cluster is quite frequent (Supplementary Figure S15).

### 3.4 Role of Aromatic Residues and Serine

**FIGURE 10:**
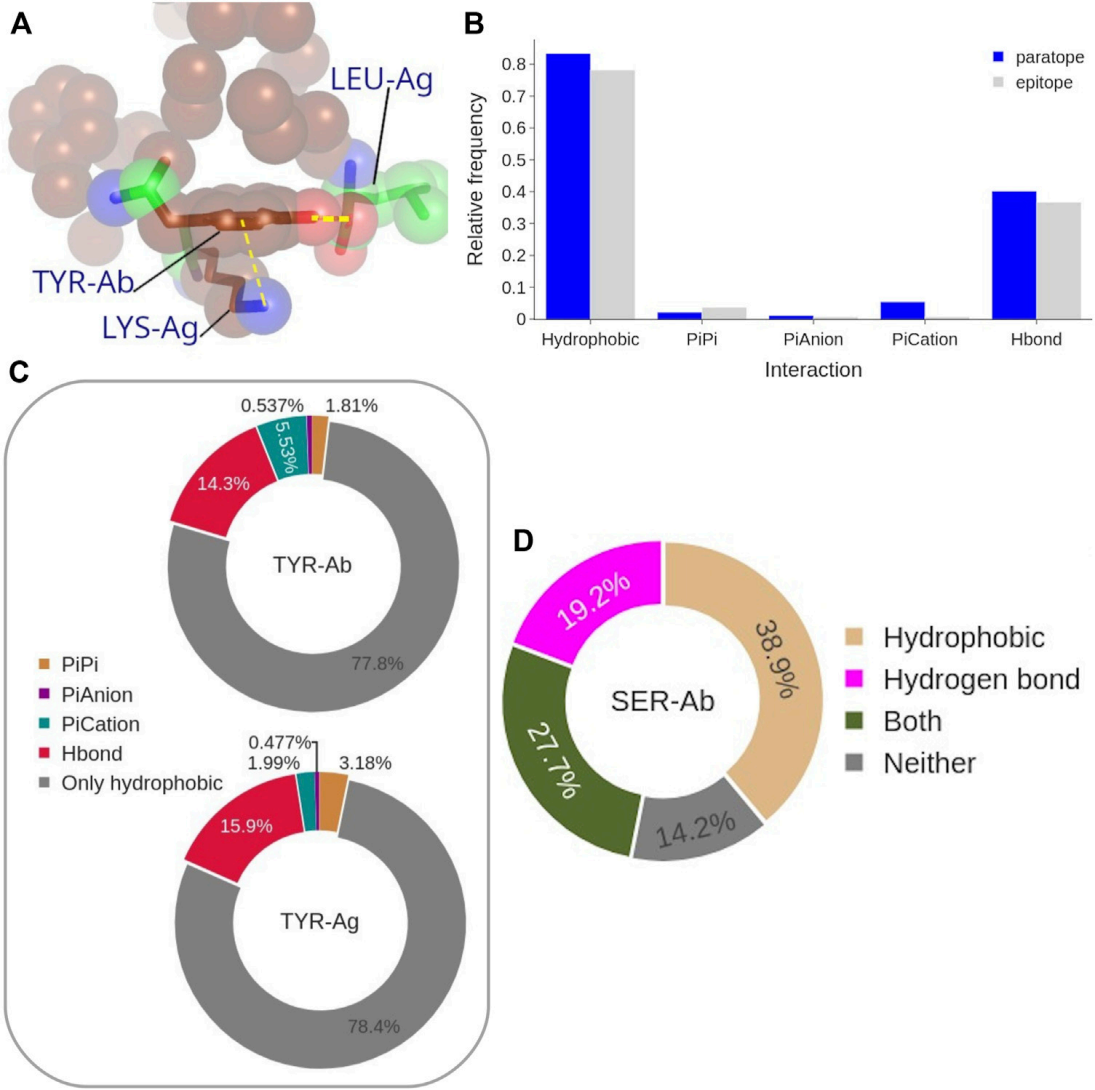
**(A)** Representative structure of coordinated interactions of Tyr from PDB: 3CVH. Tyr is participating in the main hydrophobic cluster (brown spheres) while forming H-bond and *π*-cation with two Ag residues. **(B)** Frequency of each interaction type performed by the paratope (blue) and epitope (gray) Tyrosines. **(C)** Frequency of the coordinated interactions of paratope and epitope Tyrosines that occur within hydrophobic clusters. **(D)** Frequency of paratope Ser forming H-bond, to participate in a hydrophobic cluster, both, or neither of them.

To better understand the impact on the Ab-Ag interface of the high content of Tyr, and to a minor extent other aromatic residues, in both epitope and paratope as well as that of Ser in the paratope (identified as the main players in the Ab-Ag interface in Figure 4), we analyzed in more detail their interactions. The amino acid Tyr can form different types of interactions, even concurrently, due to its aromatic ring coupled with an hydroxyl group (Figure 10A).

Aromatic ring orbitals can interact with *1*) other ring orbitals (*π* stacking), *2*) positive charged atoms (*π*-cation), and *3*) negative charged atom (*π*-anion). The procedure we used to estimate the *π*-*π* and *π*-*ion* interaction has been previously employed by (Dalkas et al., 2014), with a 4.5 Å distance threshold but a different angle criterion. The impact of the difference between the distance thresholds (5 Å instead of 4.5 Å) on our analysis was found to be negligible. Moreover, we found Dalkas’s angle criterion to be overly permissive, especially with respect to the *π*-*π* interactions. By looking at the probability of the different types of Tyr interactions to occur with respect to the interface Tyr content (Figure 10B), the most common interaction is clearly the hydrophobic one, since more than 70% of epitope Tyrosines are participating in a hydrophobic cluster. This value is even higher, up to 80%, for the paratope. Moreover, 40% of paratope and 35% of epitope Tyrosines are forming H-bonds. Aromatic interactions occur rarely, being *π*-cation interactions the most frequent aromatic interaction performed by the paratope Tyrosines (around 5% of them). In summary, 90% of all Tyr at the interface are performing at least one type of the considered interactions. This distribution is in general followed by the other aromatic residues, Trp and Phe, with the exception of their H-bond contribution which is significantly lower (*see* Supplementary Figure S14).

By looking only at the hydrophobic clusters, which are the most common type among the Tyr interactions (Figure10B), we analyzed the co-occurrence of hydrophobic interactions themselves and other Tyr interactions (Figure 10C). The results show that more than 75% of Tyr residues participate exclusively in hydrophobic clusters without participating in any other type of interaction. When Tyr is engaged in multiple interactions, the most common combination include H-bonds formation, occurring in 14% of paratope and in 16% of epitope Tyrosines in hydrophobic clusters. The aromatic interactions account for a 7% of paratope Tyrosines participating in hydrophobic clusters, while this type of coordinated interaction occurs at even lower probability in the epitope Tyrosines.

Similarly, we analyzed the role of Ser residues (Figure 10D). Over a third of Ser residues in the paratope have C atoms participating in hydrophobic clusters, while almost 20% of them are forming H-bonds. Noticeably, only 14% of Ser residues in the paratope have no role in the Ab-Ag interaction. According to this analysis, Ser residues may be located at the boundary of the hydrophobic clusters, contributing with few C atoms (backbone atoms or the C side-chain atom) and using their hydroxyl group to delineate the border of a hydrophobic cluster (*see* Supplementary Figure S16).

### 3.5 Impact of the Resolution of the Structures

The mean resolution of the analysed structures was $3.38\overset{{}^{\circ}}{A}$ , with a standard deviation of $1.92\overset{{}^{\circ}}{A}$ . In order to see how much the resolution affected our results, the analysis was performed also on the subset of complexes obtained after filtering for resolution $<=2.5\overset{{}^{\circ}}{A}$ . The filtered subset was significantly smaller, only 318 complexes, but the results of the performed analyses were substantially confirmed, with some differences in the proportion of observed interactions, especially water mediated ones. A comparative analysis on most of the studied quantities can be found in the last section of the Supplementary Data.

## 4 Discussion

The present study aims at characterizing the antibody-antigen interface taking advantage of the remarkable availability of resolved structures of this type of complexes. Table 1 summarizes the structural features of Ab-Ag interfaces that we have found.

**TABLE 1:** Summary of the Ab-Ag structural features (the most relevant, in Authors’ opinion, are in bold).

Paratope features	Results
Size	**15.6 ± 4.7 residues**
Charge	−0.4 ± 1.8. **Close to neutral**
Chain contribution	53% CDR H, 29% CDR L, 14% FR H, 4% FR L
Amino acid composition	High occurrence: **Tyr**, Ser, Trp, Gly
	Low occurrence: Cys, Met, Gln, Lys, His
Other features	CDR H3 lenght modifies other CDRs binding at short interval range
	**Participation of Fw residues to binding is common and not negligible**
**Epitope features**	
Size	**14.6 ± 4.9 residues**
Charge	0.3 ± 2.0. **Close to neutral**
Surface characterization	75% is constituted by one single patch
	When multiple patches, 80.0% is covered by the broadest patch
exposedness	Deep: 29 ± 13%, medium: 38 ± 15%, superficial: 34 ± 16%
	**more than 70% is either at a high or medium exposedness, that is accessible to the CDR backbone**
Segmentation	80% epitopes have three to eight linear stretches of one to six residues
	the longest stretch contains five to seven residues
Secondary structure	**Enriched in coils**, depleted of helices and strands
Amino acid composition	Enriched: Tyr
	Depleted: aliphatic residues, Ser, Cys
**Ab-Ag interaction**	
Interface size	(1068 ± 314) Å^2^
Hydrophobic	Over 80% of the interfaces have 1 or 2 hydrophobic clusters
	**Biggest cluster of 65 ± 24 C atoms (22 ± 7 residues)**
	**located in 3 ± 1 CDRs**, and **centered on H3** in the paratope
Polar atoms	pKa shifts towards the stabilization of the most probable state at pH 7
	Ab side-chain: H-bonds (48%), salt bridges (12%), water-mediated (13%)
	Ag side-chain: hydrophob. shield (32%), H-bonds (42%), salt bridges (11%)
	**Polar bonds are mainly found in CDRs H3, L3 and H2**
Role of Tyr	**Hydrophobic cluster:** 75% of epitope Tyr, **80% of paratope Tyr**
	**H-bonds:** 35% of epitope Tyr, **40% of paratope Tyr**
	Marginal contribution of *π*-cation and *π*-anion ( < 5% of Tyr)
	**>** **20% of Tyr perform more than one type of interaction**
	15% of Tyr in hydrophobic clusters forms H-bond
Role of Ser	**Located at the border of hydrophobic clusters**
	> 50% form H-bonds

The present analysis found many agreements with previous works analysing the *paratope* structure. For example, we confirmed the participation of non-CDR residues to the binding (between one and four framework residues). This result, however, still supports the idea that CDRs should be considered the most important Ab regions for binding, while the framework has a more limited, but still not negligible, role. The paratope size has been previously estimated to be ranged between 15 and 25 residues (Rubinstein et al., 2008; Ramaraj et al., 2012; Stave and Lindpaintner, 2013). We found a bit lower number: about 15 residues. This broad variation depends on the data set dimension, but also on how the Ab-Ag interface is defined. To identify the Ab-Ag interfaces we used the buried Solvent Excluded Surface definition. In contrast with interfaces defined by employing cut-off distances between atoms of the binding partners, SES well captures the details of the interface, including the creation of possible cavities that can play a significant role in binding. Previous works that also preferred the surface-based methods used the Solvent Accessible Surface definition, which is easier to calculate, although less accurate. While we did not perform a systematic comparison, we however advocate the adoption of the SES definition, since it better corresponds to the intuitive concept of molecular surface. The amino acid composition of the paratope is one of the most extensively analyzed features. There is a global consensus in the over-representation of Tyr in the paratope (Wang et al., 2018). Most of the previous works also agree with our results of the higher representation of Ser and aromatic residues, as well as the depletion of Lys, Pro, Met and Cys. Indeed, the amino acid paratope occurrences reported by Nguyen et al. (2017) are very similar to those obtained here. Wang et al. (2018) compared the amino acid propensities in the paratopes with a database of non-antibody protein-protein complexes, reporting also a depletion of the hydrophobic aliphatic amino acids. We found very similar values also for the analysed Ab-Ag interfaces, containing around 5% of aliphatic amino acids. These values are certainly higher that the frequencies observed in our paratope database for the hydrophobic aliphatic residues.

The prominent role of *Tyrosines*, and to a lesser extent, of other aromatic residues, in the Ab-Ag interfaces has been often observed in previous works. However, the reasons for this occurrence are still debated. The presence of Tyr in the paratope is overwhelming in comparison to other amino acids, while they are only slightly enriched in the epitope. In fact, the paratope amino acidic composition is restricted because antibodies are all derived from the germ line sequences, which are rather limited. Many of the sequences of the D regions within H3 already contain several Tyrs and therefore the latter remain even after affinity maturation. One possible explanation is that Tyrosines were selected during evolution to enhance binding capacity. The vast majority of paratope Tyr are participating in hydrophobic clusters. One third of hydrophobic-participating Tyr is also interacting with a polar atom, *via* H-bond, water-mediated, pi-cation or pi-anion contacts. Therefore, we agree with the explanation of Dalkas et al. that the capability of Tyr to be the “jack of all trades,” able to participate in all types of interactions except salt bridges could be the main reason of its high occurrence in paratopes (Dalkas et al., 2014). The similar abilities of Trp could be counterbalanced by its larger size, which might produce steric hindrance in some cases. On the other hand, Wang *et al.* argued that the reduced cost of the side chain entropy of aromatic residues could be the responsible of this enrichment (Wang et al., 2018). However, this justification alone can not explain Tyr rather than Trp or Phe enrichment.

Ser residues have also a marked presence in the paratope according to both our analysis and previous works (Ramaraj et al., 2012; Peng et al., 2014; Wang et al., 2018). We found that they play an important role as residues at the boundaries of hydrophobic clusters. Almost one-third of Ser in the paratope form H-bond at this location, balancing the disruption of the hydrophobic cluster with this specific interaction. Its presence in detriment of larger size polar residues, such as Thr or Asn, could be related to steric effects, since the small size of Ser residues balances the overpopulation of Tyr in the CDRs. In this respect, the high frequency of Gly in the paratope would follow the same reasoning, even though Gly lacks the ability to form H-bonds with its side-chain.

Another important structural aspect of the binding region is the length of CDR H3, already performed in the previous work of Tsuchiya *et al.* (Tsuchiya and Mizuguchi, 2016). We agree with their work that the length of H3 affects the binding of the other CDRs, but only when H3 contains less than 10 residues. Above this length, the participation of other CDRs to the paratope is minimally affected by the length of H3. This observation could be interpreted by considering that, above a certain chain length, the H3 loop might acquire a secondary structure in which the accessible residues for binding become limited and their influence on other CDRs is constant.

We found numerous agreements with the existing literature concerning *the epitope*. For instance, epitopes are found to be enriched in flexible coil structures and depleted of helix and strand structures (Rubinstein et al., 2008; Kunik and Ofran, 2013; Dalkas et al., 2014). Moreover, even with our SES-based definition, we found that more than 70% of the epitope surface is located in the most exposed regions of the antigen surface (Novotny et al., 1986; Rubinstein et al., 2008). These findings indicate that flexibility and solvent exposure are important factors in antigenic regions, probably because they favor a stronger binding. Regarding the epitope size, previous works estimated a [13–22 aa] interval (Sun et al., 2011; Ramaraj et al., 2012; Kringelum et al., 2013; Stave and Lindpaintner, 2013). We confirm this and find an average size of 15 residues. The amino acid composition of the epitope has also been extensively studied (Kringelum et al., 2013) reported that the differences of amino acid content of epitopes with respect to non-epitope surfaces are not statistically significant, as we also observed. Nevertheless, there is a consensus that epitopes are enriched in charged amino acids, present an over-representation of Tyr and Trp, and are depleted in aliphatic residues (Rubinstein et al., 2008; Sun et al., 2011; Kringelum et al., 2013; Kunik and Ofran, 2013). Wang et al. (2018) affirmed that the paratopes are mostly negatively charged while epitopes have an excess of positively charged residues. However, in our analysis we do not find any significant net charge preference either in the epitope or in the paratope, in favour of a clear bias towards net neutral interfaces.

In agreement with other findings, we observe that the majority of epitopes are conformational. This highlights the importance of structural data and related techniques, such as X-ray diffraction, to deeply understand the subtle Ab-Ag binding mechanisms. This must also be taken into account, in experimental identification of antibodies, and compels the development of more predictive computational design protocols, which are capable of leveraging available structural information. Moreover, our analysis supports the idea of Kringelum et al. (2013) that conformational epitopes can be seen as the combination of sequential patches. Indeed, we updated this information showing that 80% of epitopes contains three to eight different sequential patches, many of them containing only a few residues (1–3). However, the longest patch usually contains five to seven residues.

To characterize the *Ab-Ag binding*, we evaluated the most common types of interactions. Our results agree with Dalkas *et al.* in that the most frequent interactions are hydrogen bonds together with hydrophobic interactions (Dalkas et al., 2014). Interestingly, Dalkas et al. (2014) observed that hydrophobic interactions are more prevalent in non-antibody protein–protein interfaces than in Ab-Ag ones. Moreover, Shehata et al. (2019) showed experimental evidence that antibodies after somatic mutations have reduced levels of hydrophobicity compared to germline-encoded ones. A possible interpretation of this is that germline-encoded Abs make a larger use of hydrophobic interactions since they aim at a larger spectrum of potential antigenic targets. During maturation, interactions such as H-bonds, salt bridges or even water-mediated contacts prevail since they confer a better specificity towards given targets.

*Polar bonds* are considered an important source of specificity for antibodies. We observed a significant percentage of residues at the interface forming polar bonds and at the same time participating to a hydrophobic cluster. This scenario is compatible with their being located at the boundaries of the hydrophobic clusters. This configuration seems to allow for specificity and energetically balances the decrease of hydrophobic size of the interface. In this respect, the antibody affinity maturation process in the cell might explain why the number of polar atoms in the Ab that only perform hydrophobic shielding is significantly lower than that in the Ag.

We wondered if in general the Ab-Ag interaction could be approximated as a combination of CDR-Ag interactions. The idea of segmenting Ab-Ag interfaces into independent CDR-Ag units is certainly intriguing and potentially useful for optimizing the functioning of predicting algorithms. Our analysis of the interface formation with NanoShaper (Decherchi and Rocchia, 2013) suggested that a low number of epitope residues are interacting simultaneously with two CDR loops. However, the hydrophobic analysis indicated that these residues may play essential roles in the formation of the hydrophobic cluster, since the biggest cluster usually is contributed by more than one CDR loop. Therefore, approximating the Ab-Ag interfaces as a combination of CDR-Ag would require also taking into account the stability of the hydrophobic cluster.

Considering the studies performed over the last 10 years on this topic, we would like to stress the importance to reach a consensus on what features best characterize this important process. This could be achieved by comparing the different studies and possibly updating them in parallel with the increasing availability of structural information. To draw a timeline, a structural analysis of about 10 years ago involved 53 structures (Ramaraj et al., 2012) while at present, more than 1000 structures are available. We hope this work is a positive step in this direction. In this context, the weekly update of the SabDab database (Schneider et al., 2021) is noteworthy and supports the development of this virtuous circle. On the other hand, we note that only 14% of entries in the SabDab have binding affinity information, i.e. only 746 out of 5426 structures (including non-protein targets). Much more affinity data would be needed to permit the establishment of a real quantitative structure-activity relationship and the assessment of the impact that each of the mentioned descriptors has on binding. This information should be needed not only for high affinity complexes, but also for low and intermediate cases, as well as for single mutations, to better capture also the subtler details.

In summary, an exhaustive structural analysis of the biggest antibody-antigen database as of today has been here performed. We developed an accurate method to identify and characterize hydrophobic clusters in protein-protein interfaces, which is available for the community together with the code repository in https://github.com/concept-lab/AbAgInterface. This analysis shed light to the mechanism of binding of antibodies and its relationship with their physiological maturation process. Further analysis, such as on the role of water molecules or on the relationship between binding affinity and Ab-Ag binding conformation, should be performed in the future, as long as the number of available high-resolution structures including water molecules or the number of structures with binding affinity information increase.

## Data Availability

The datasets presented in this study can be found in online repositories. The names of the repositories and accession number(s) can be found below: https://github.com/concept-lab/AbAgInterface.
