# Supplementary material for: Antibody-Antigen Binding Interface Analysis in the Big Data Era
Source: Front Mol Biosci. 2022 Jul 14;9:945808. doi: 10.3389/fmolb.2022.945808 (PMC9329859; doi:10.3389/fmolb.2022.945808)
Supplement: Supplementary file 2 [file DataSheet1.pdf]

Supplementary Material

1 SUPPLEMENTARY DATA

2 SUPPLEMENTARY TABLES AND FIGURES

2.1 Figures

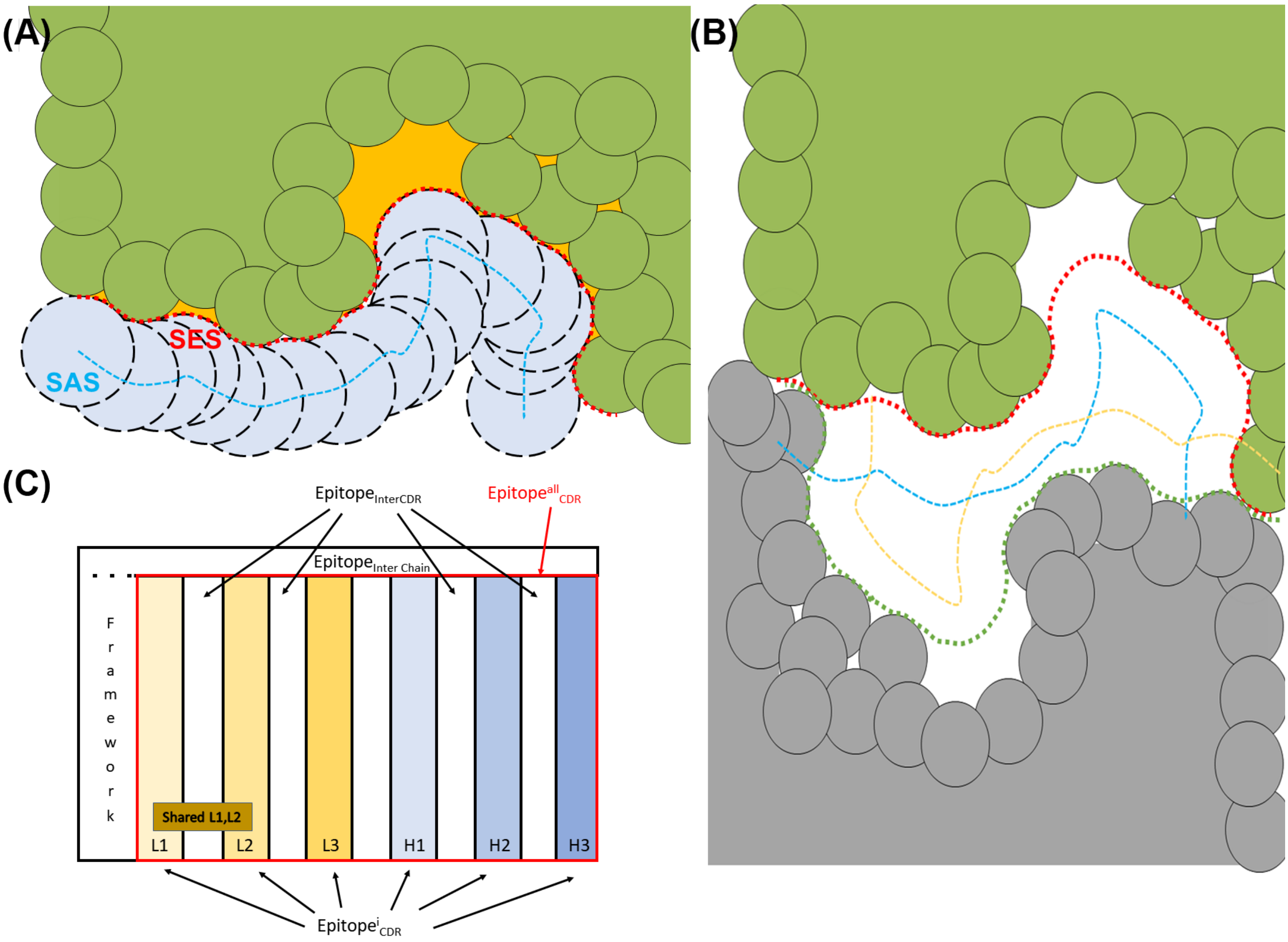

Figure S1. (A) Pictorial representation of the Solvent Accessible Surface (SAS) and the Solvent Excluded Surface (SES) definitions for a molecule, represented as union of possibly overlapping spheres, in green. In light blue, the probe sphere representing the solvent, $R = 1.4\AA$, rolls over the molecule. The blue dashed lines connects the centers of the probe spheres and generates a 2D representation of the SAS. The red dotted line represents the SES. The orange region is the volume not occupied by the molecule that the probe cannot access. (B) When two binding partners, here in green and grey, approach, there is a significant overlap of the volumes enclosed by their SASs, here in blue and light orange, also in the case of good complementarity, here only at the right and left borders of the binding interface. In contrast, in the regions of good complementarity, the two SESs, here in red and green dots, tend to coincide. (C) A schematic diagram of the epitope regions according to their interaction with the different portions of the paratope.

‘

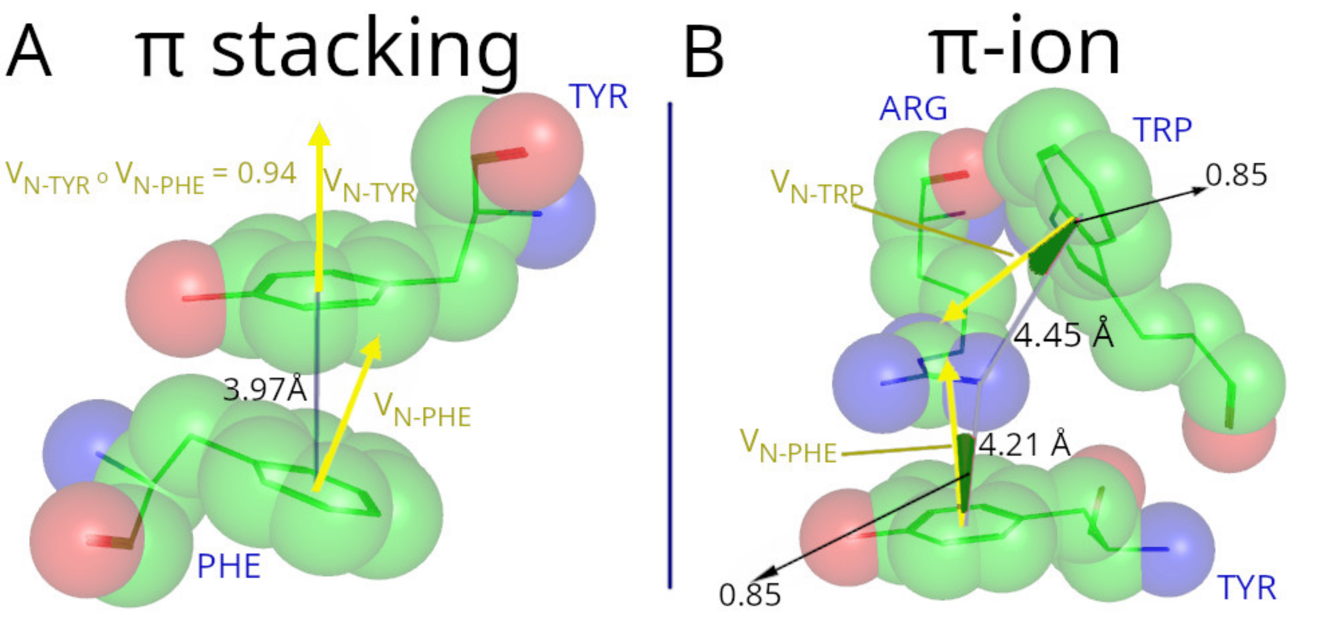

Figure S2. Representative examples of π stacking and ion-π interactions. A) Representation of a π stacking interaction from PDB id: 7ND7. B) Representation of two cation-π interactions from the PDB id: 5I5K.

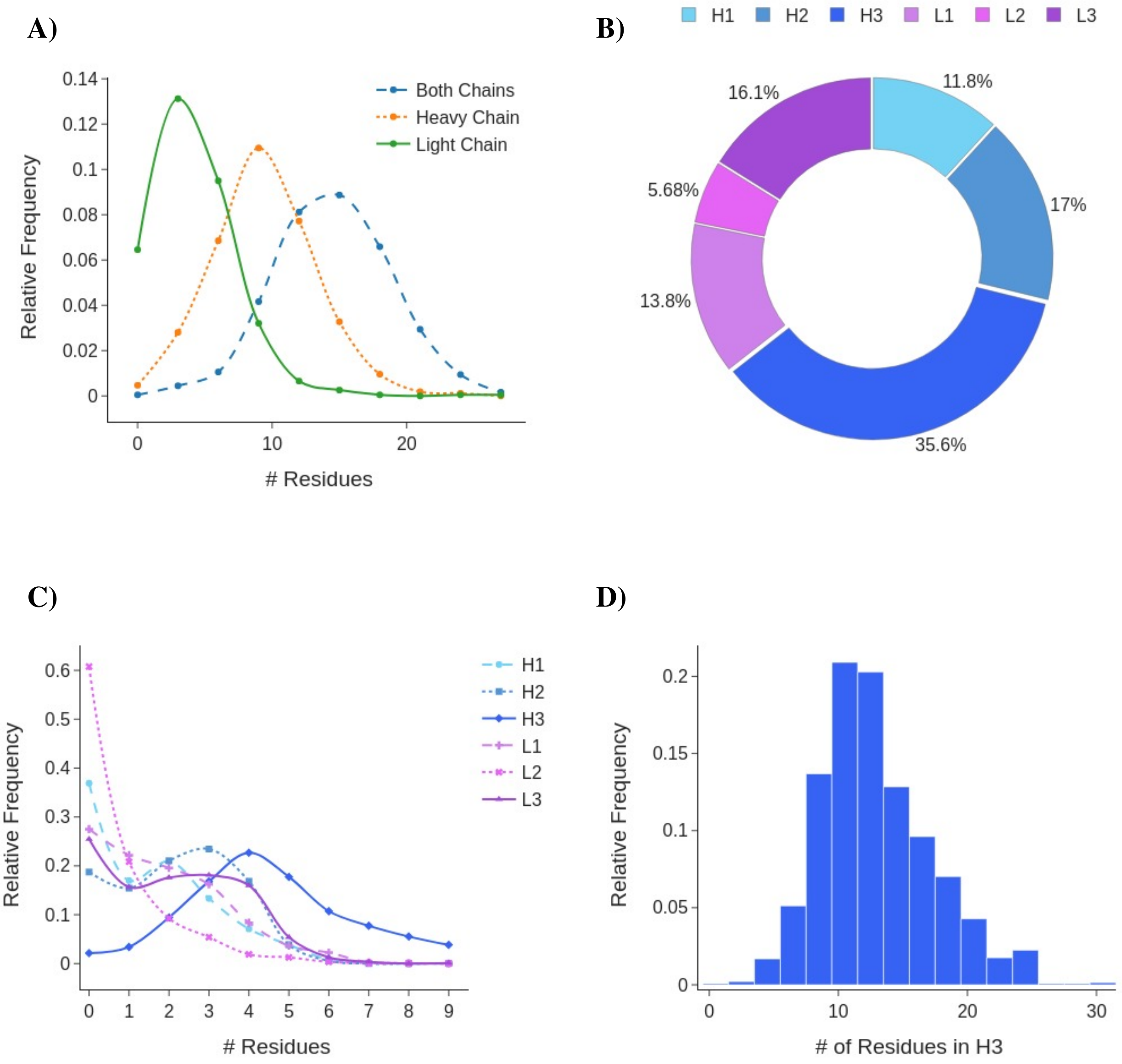

Figure S3. Additional structural features of the paratope. A) Distribution of the number of residues by chain at the paratope. B) Contribution in percentage of each CDR to the paratope. C) Distribution of the number of residues of each CDR that participate in the paratope. D) Distribution of the number of residues in the CDR H3.

A)

B)

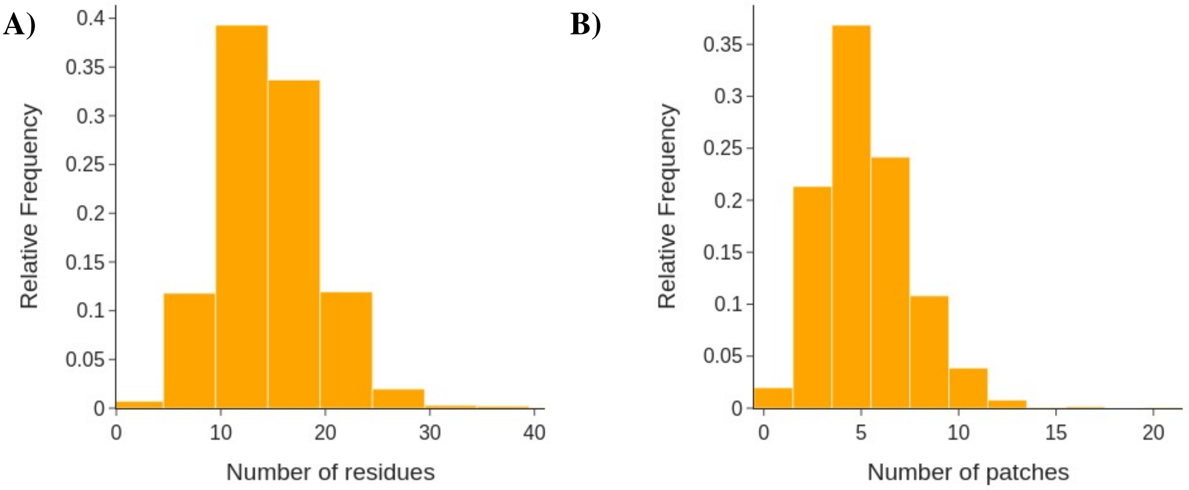

Figure S4. Additional structural features of the epitope. A) Distribution of the number of residues at the epitope. B) Number of continuous patches per epitope. A tolerance for gaps of 1 residue was considered.

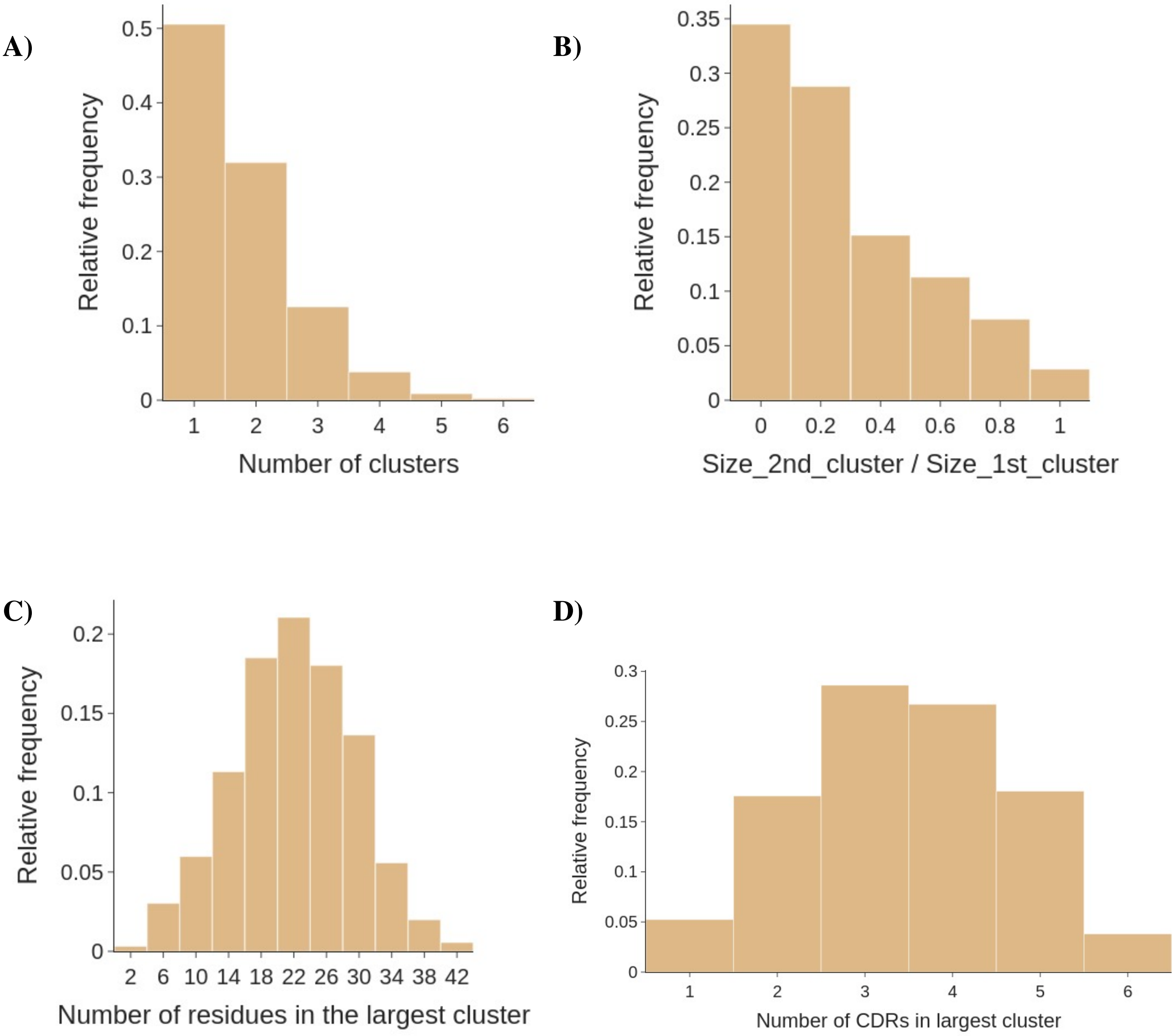

Figure S5. Additional characterization of hydrophobic clusters. A) Distribution of the number of hydrophobic clusters per Ab-Ag complex. B) Distribution of relative sizes between the biggest and the second biggest hydrophobic clusters. The sizes are expressed in number of C atoms contained in each cluster. C) Distribution of the number of residues in the biggest hydrophobic cluster. D) Distribution of the number of CDRs that participate in the largest cluster.

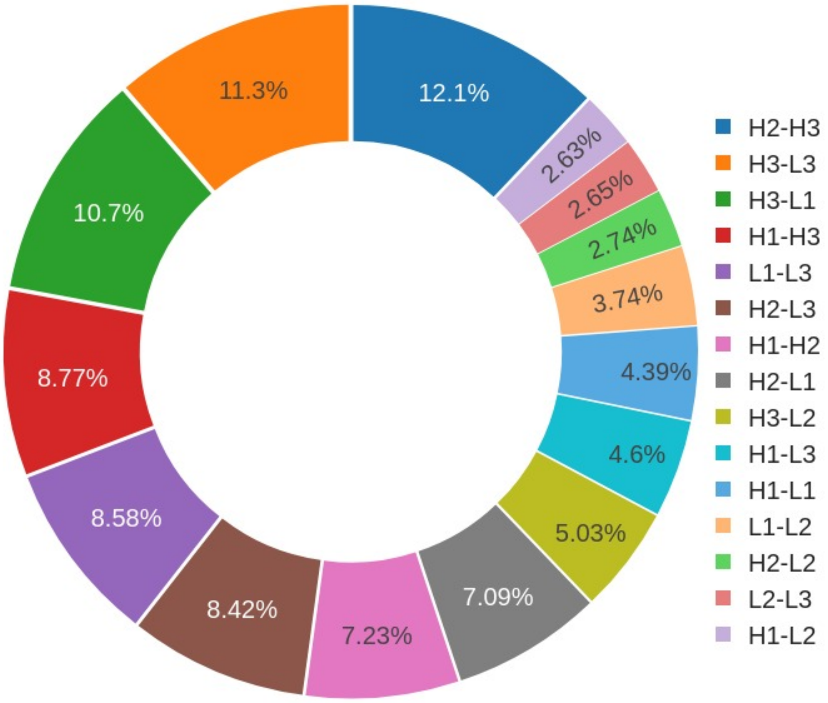

Figure S6. Distribution of the coordinated participation of 2 CDRs to the biggest cluster.

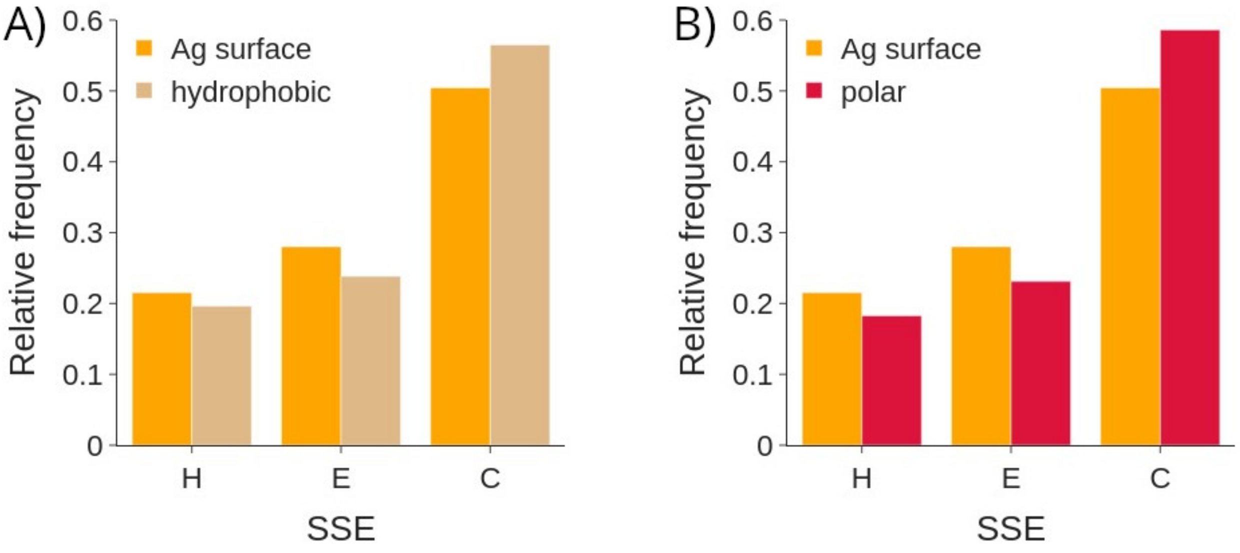

Figure S7. Distribution probability of the main secondary structure motifs of epitope residues participating to A) hydrophobic and B) polar interactions. The different secondary structure elements were grouped in helix (H), strand (E), and loops (C).

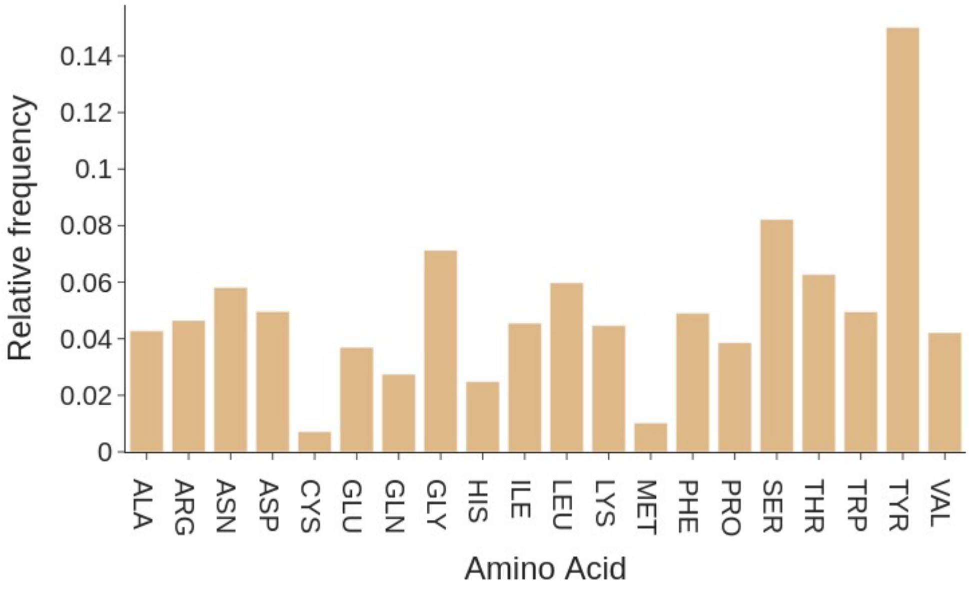

Figure S8. Amino acid participation in the biggest hydrophobic cluster, evaluated by counting once the residues that has at least one C atom participating in the cluster. Tyrosines are still the most over-represented, but now Serines and Glycines rise their participation with respect to to the Figure 6E).

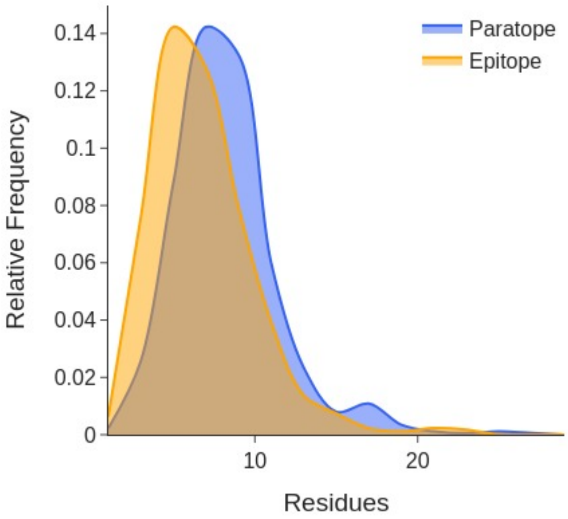

Figure S9. Distribution of the number of titratable residues in the paratope and epitope.

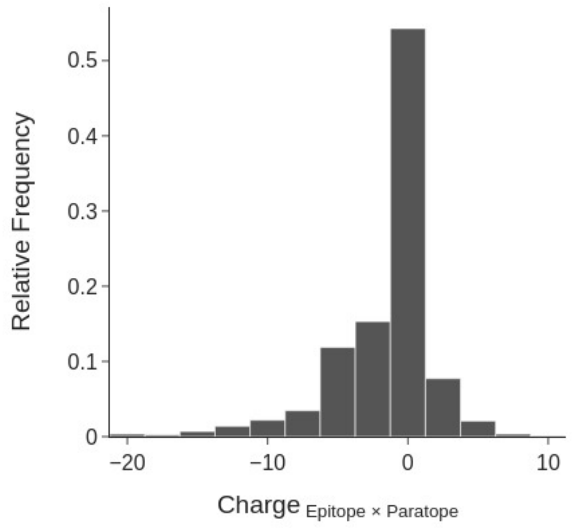

Figure S10. Distribution of the product between epitope and paratope net charges.

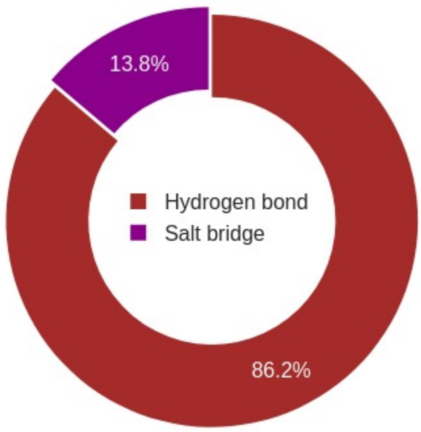

Figure S11. Distribution of polar interactions in the interface.

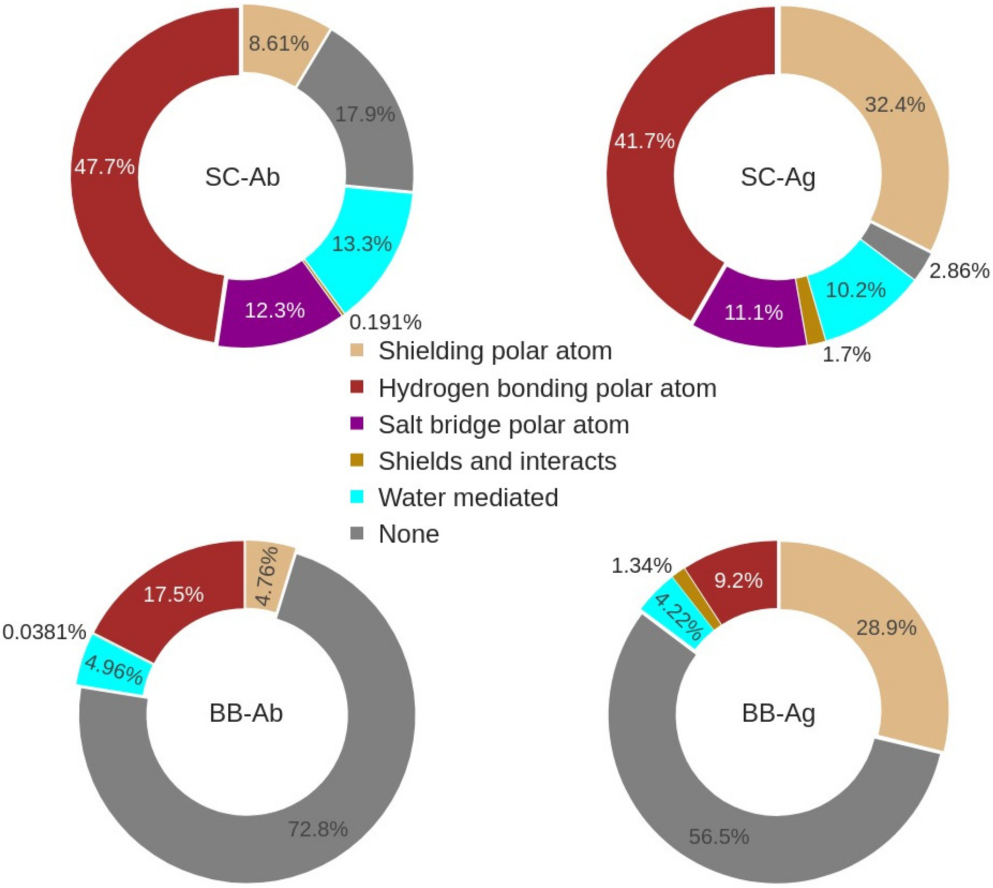

Figure S12. Probability distribution of the different roles of polar interactions including interfacial waters.

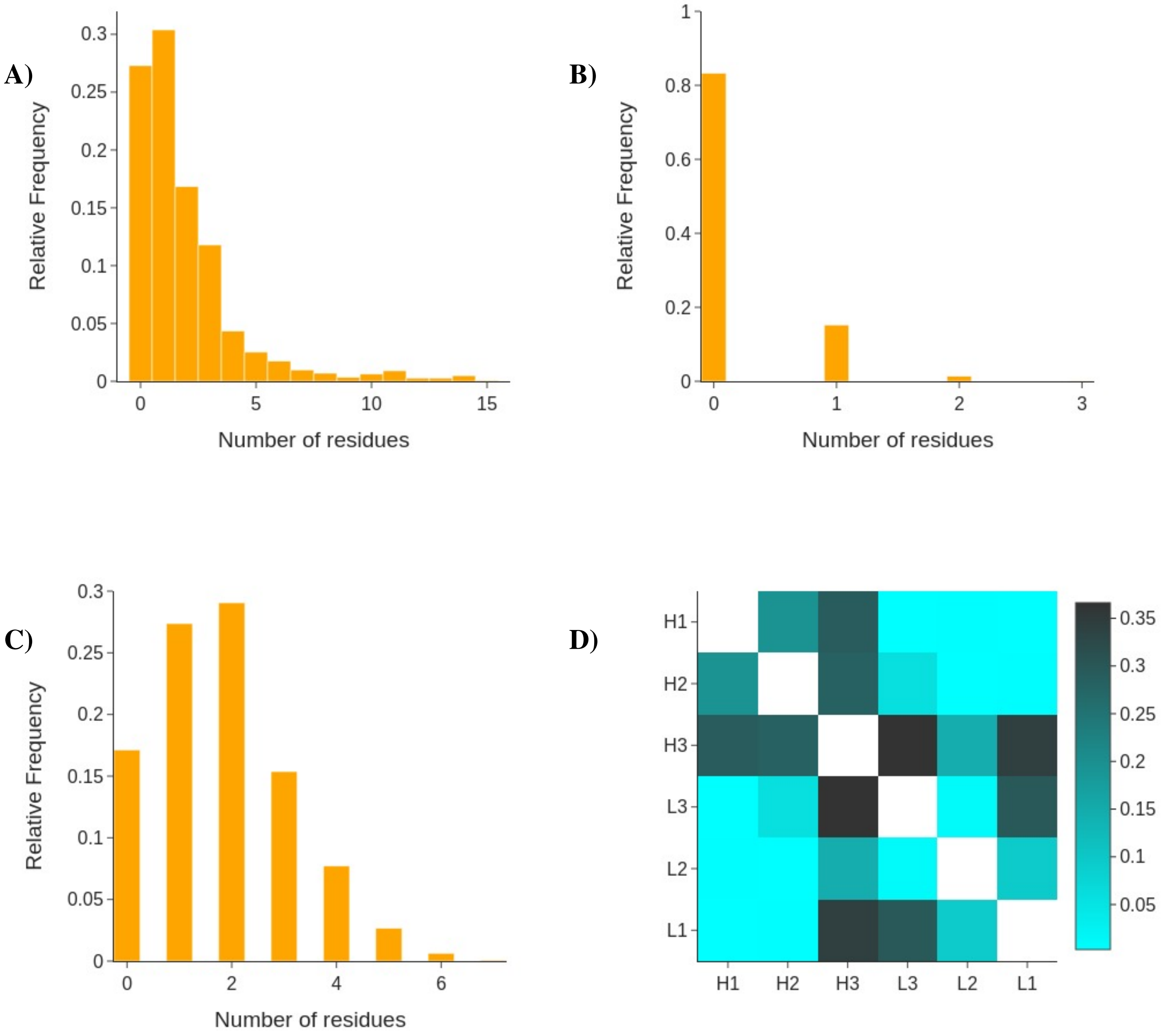

Figure S13. Cooperativity of CDRs in Ab-Ag binding. A) Distribution of Epitope$_{ExtraCDR}$ residues per Ab-Ag complex, the set of epitope residues that are not directly buried by any single CDR. B) Distribution of Epitope$_{InterChain}$ residues per Ab-Ag complex, residues that are only solvent excluded by the presence of the Ab Heavy and Light chains. C) Distribution of Epitope$_{Shared}$ residues per Ab-Ag complex, residues that are solvent excluded simultaneously by two or more CDRs. D) Matrix of interaction between the different CDRs.

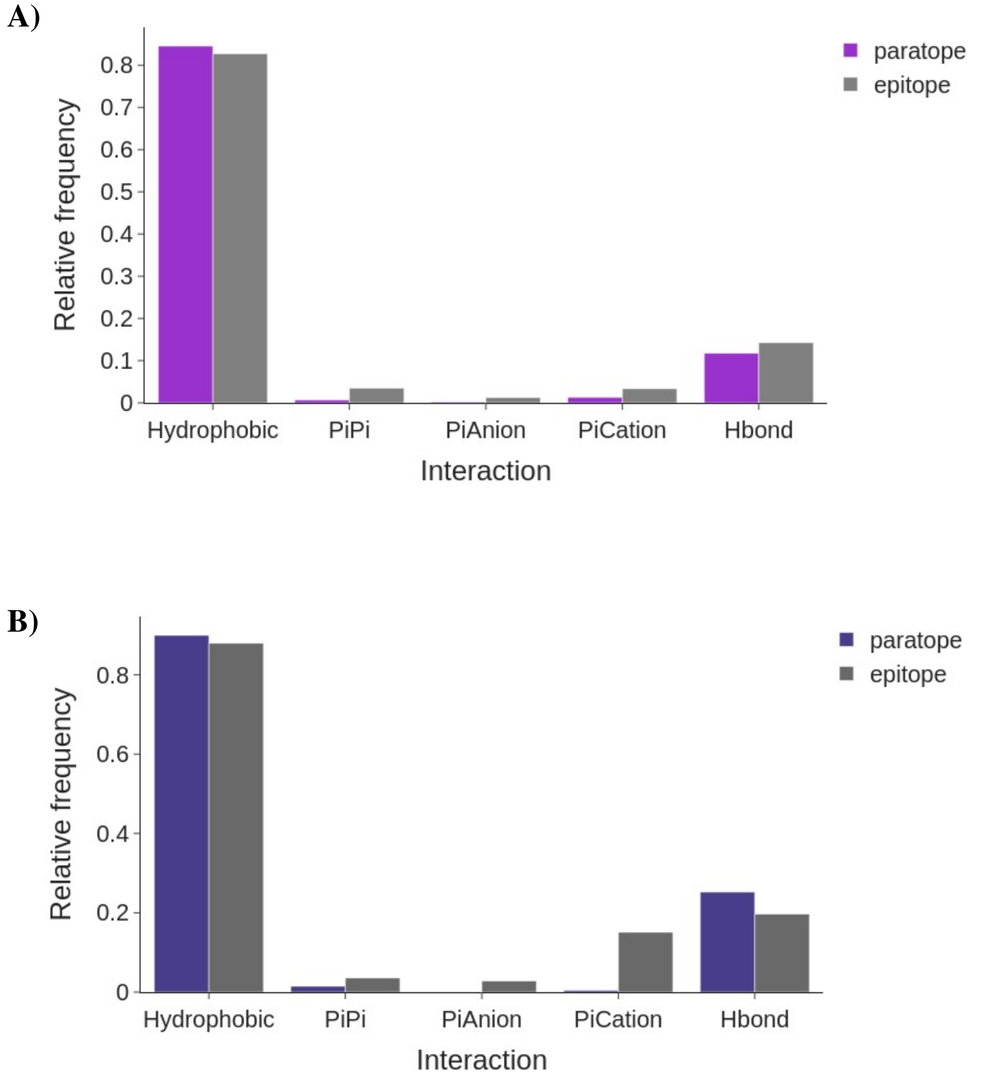

Figure S14. Type of interactions made by A) Phenylalanine and B) Tryptophan residues in the Ab-Ag interface.

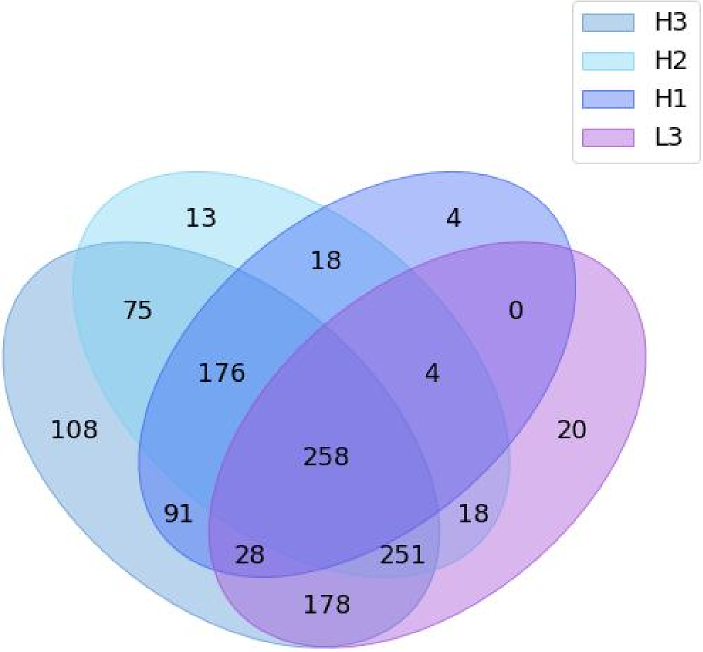

Figure S15. Numbers correspond to the structures that match the criteria and add up to 1242 structures of 1260, that is, only 18 structures don't include any of these 4 main CDRs. The main combinations are **H3-H2-H1-L3**, **H3-H2-L3**, **H3-L3** and **H3-H2-H1**, in order of relevance.

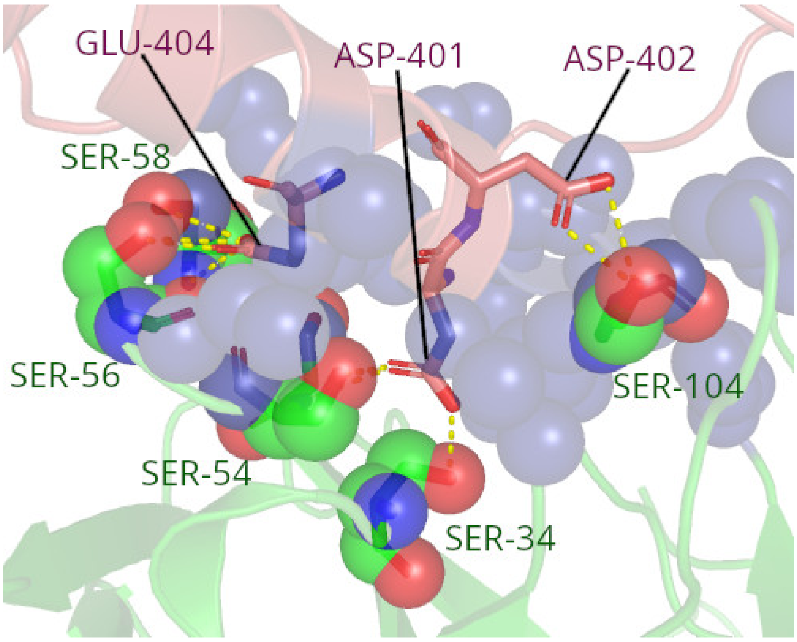

Figure S16. Close up of the Ag (salmon) and the Ab (green) from PDB id: 5VK2. 5 Serines (58, 56, 54, 34 and 104; shown as sticks and spheres), outlining a hydrophobic cluster (translucent blue spheres), while forming hydrogen bonds with 3 antigen residues (GLU404, ASP401, ASP402; shown in salmon colored sticks).

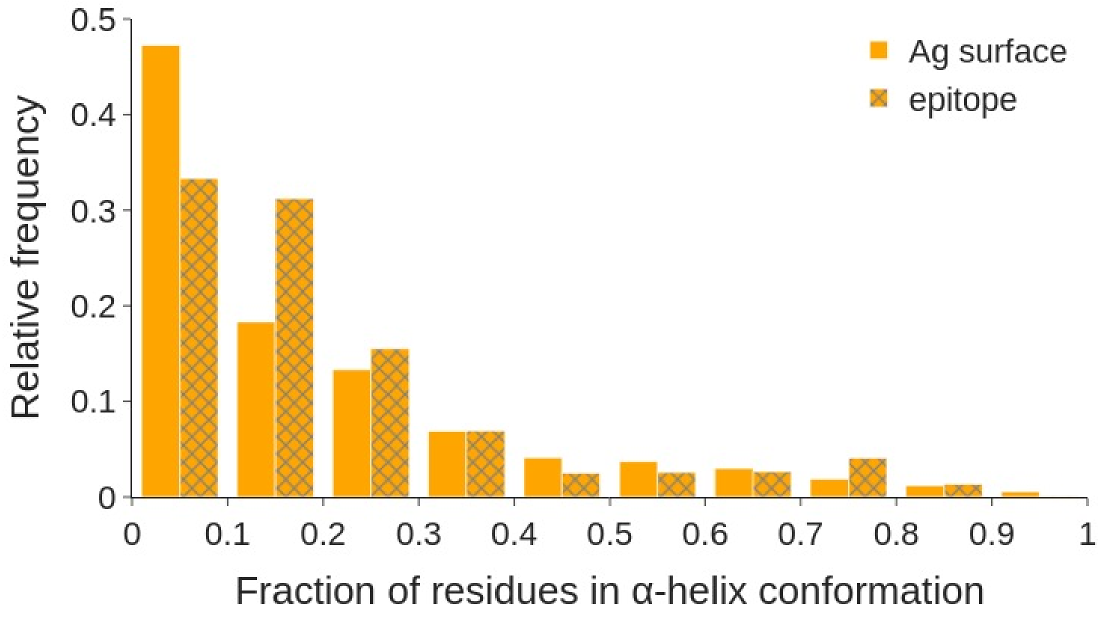

Figure S17. Distribution of residues in -helix conformation for the Ag surface and epitope.

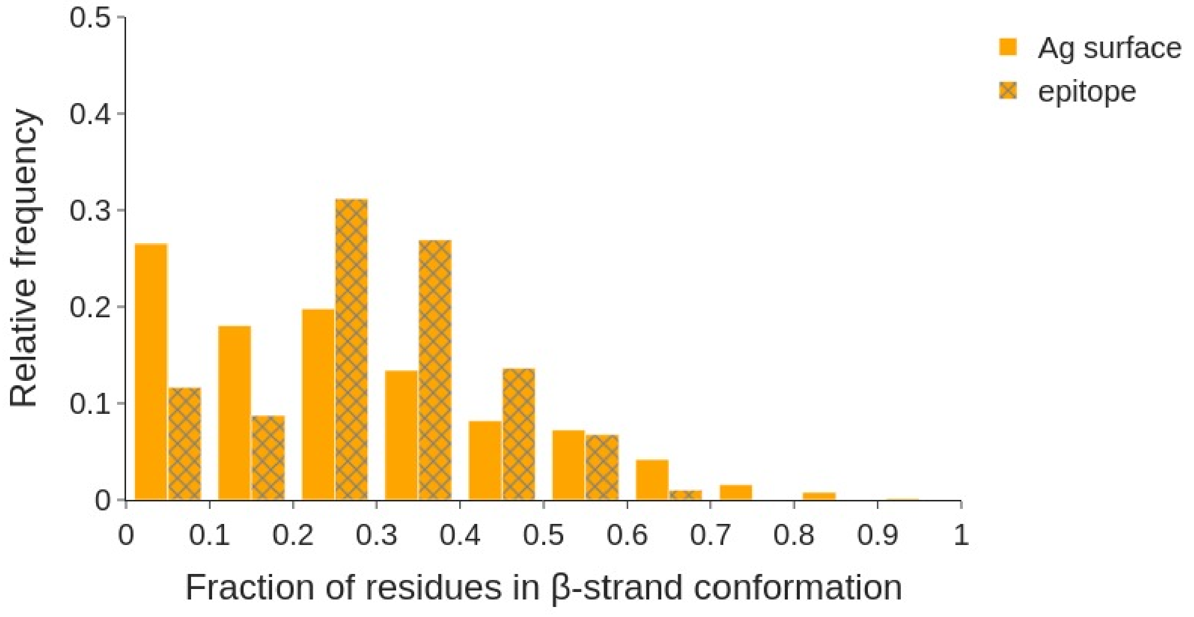

Figure S18. Distribution of residues in -strand conformation for the Ag surface and epitope.

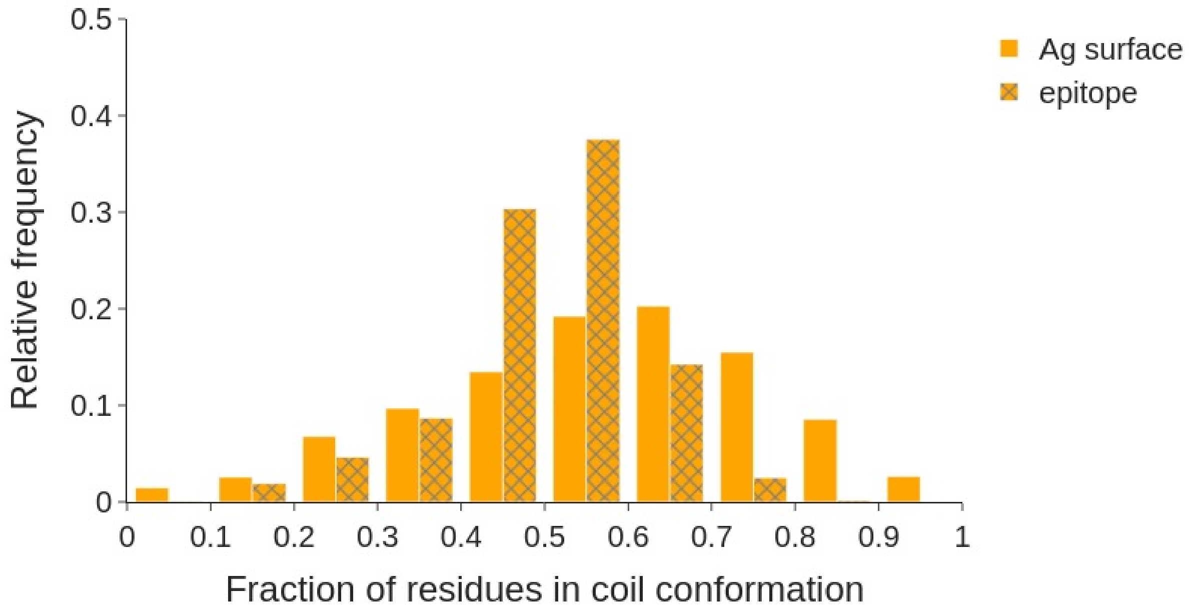

Figure S19. Participation (in %) of each CDR to the biggest hydrophobic cluster.

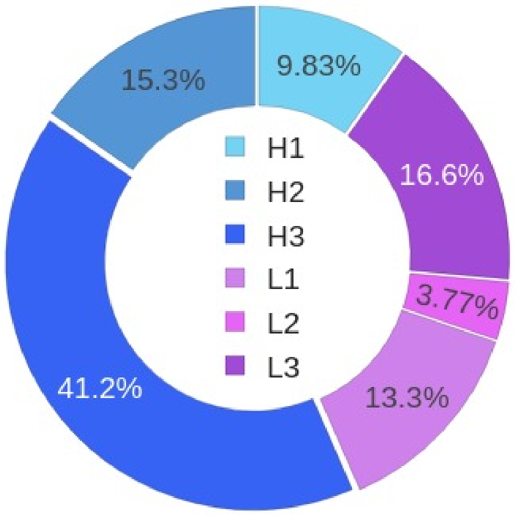

Figure S20. Participation (in %) of the polar atoms in each CDR to the polar bonds, for the reduced subset of structures with a resolution lower than 2.5A.

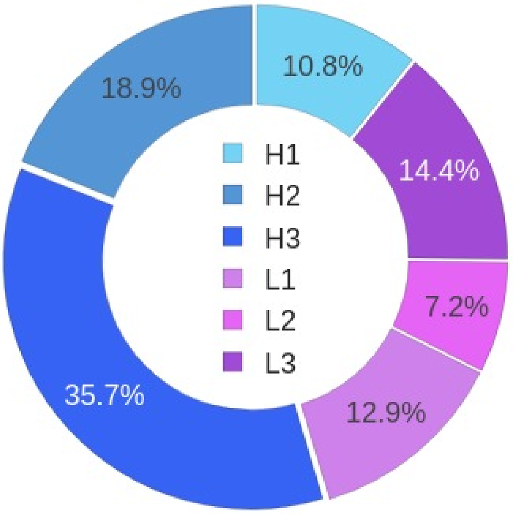

Figure S21. Participation (in %) of the polar atoms in each CDR to the polar bonds, for the reduced subset of structures with a resolution lower than 2.5A.

3 COMPARISON WITH HIGH-RESOLUTION SUBSET

In the following pages, some comparative results between the results on the full dataset and those on the filtered one, res $< 2.5\mathring{A}$, are presented in the form of: current figure on the right and corresponding figure calculated on the filtered subset, on the left.

Results for filtered set
(filtered <2.5Å
318 complexes)

Results on the entire dataset
(no filter on resolution
1425 complexes)

Figure 5C

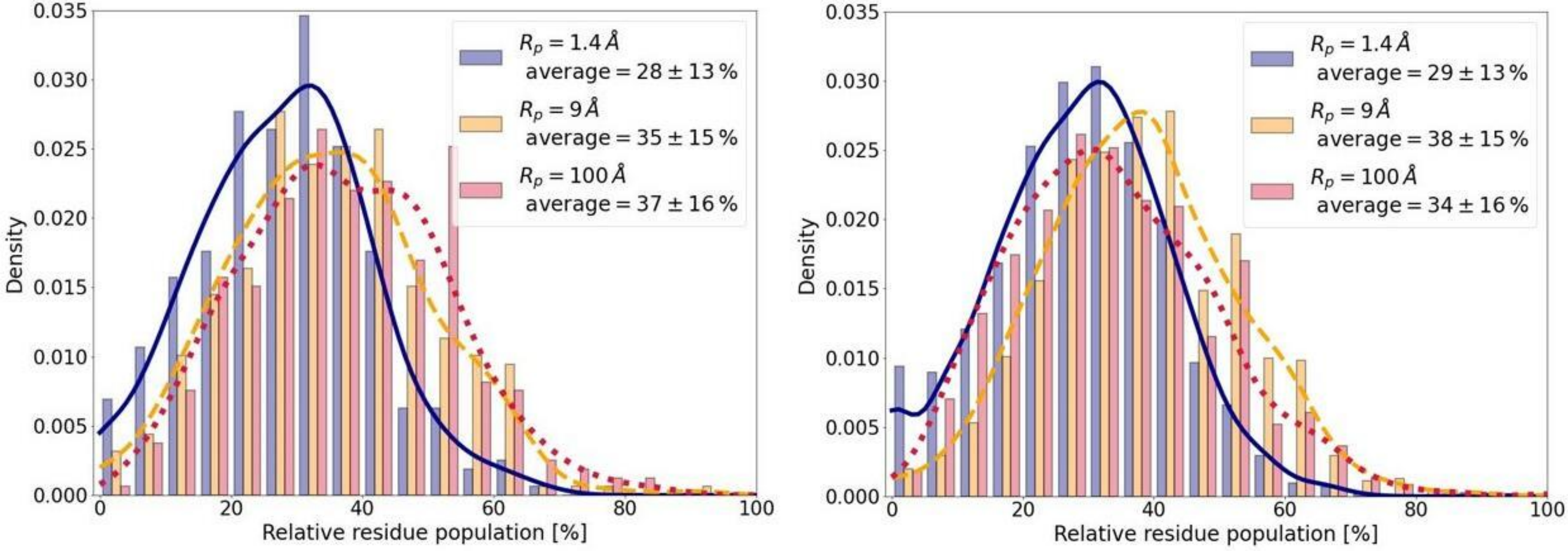
$R_p = 1.4\,Å$
average = 28 ± 13 %
$R_p = 9\,Å$
average = 35 ± 15 %
$R_p = 100\,Å$
average = 37 ± 16 %
Density
Relative residue population [%]
$R_p = 1.4\,Å$
average = 29 ± 13 %
$R_p = 9\,Å$
average = 38 ± 15 %
$R_p = 100\,Å$
average = 34 ± 16 %
Density
Relative residue population [%]

Figure 5D

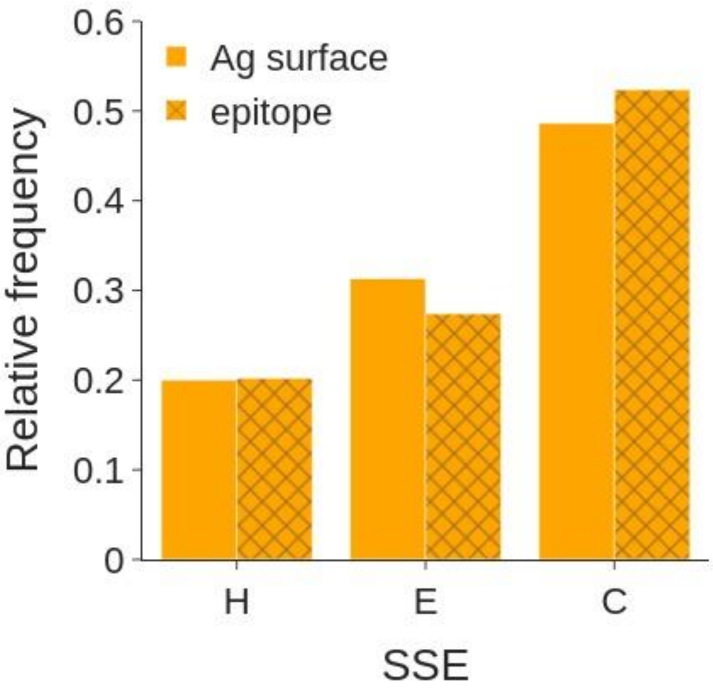

0.6
0.5
0.4
0.3
0.2
0.1
0
Relative frequency
Ag surface
epitope
H
E
C
SSE

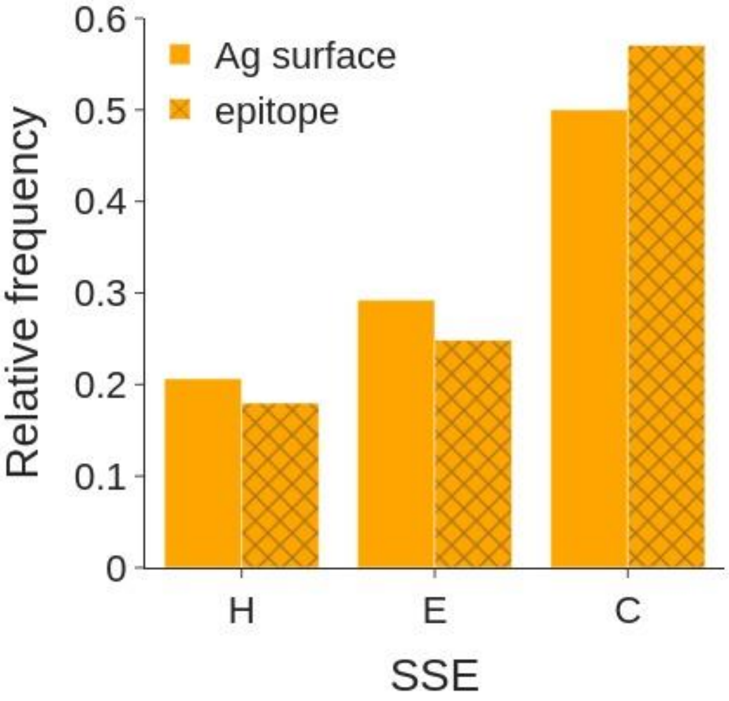

0.6
0.5
0.4
0.3
0.2
0.1
0
Relative frequency
Ag surface
epitope
H
E
C
SSE

Figure 6B

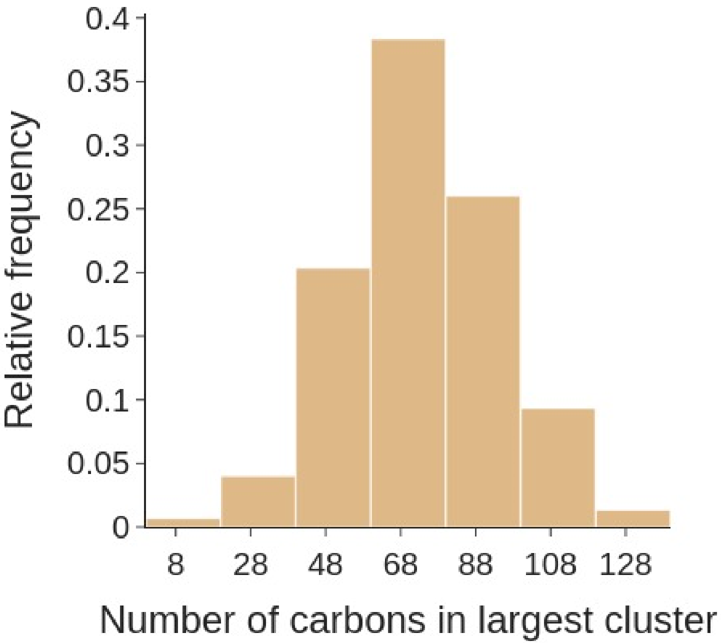
Relative frequency
Number of carbons in largest cluster
0.4
0.35
0.3
0.25
0.2
0.15
0.1
0.05
0
8
28
48
68
88
108
128

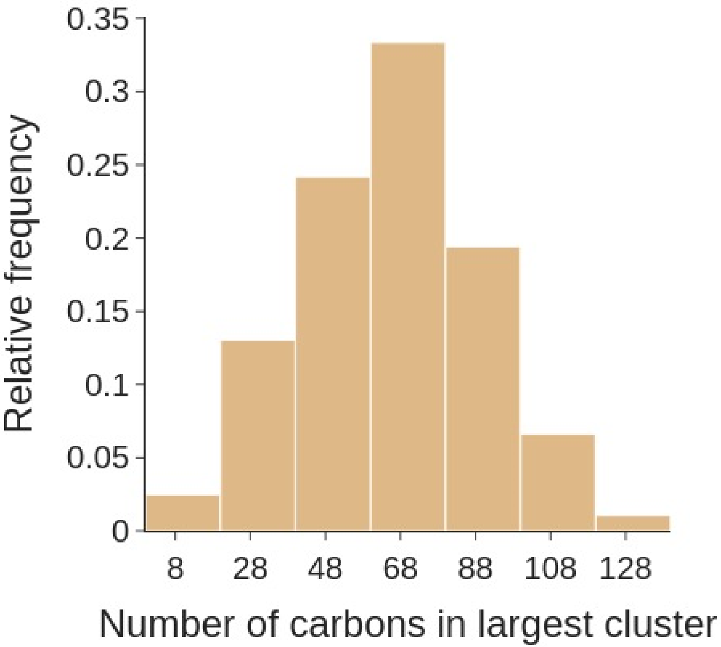
Relative frequency
Number of carbons in largest cluster
0.35
0.3
0.25
0.2
0.15
0.1
0.05
0
8
28
48
68
88
108
128

Figure 6C

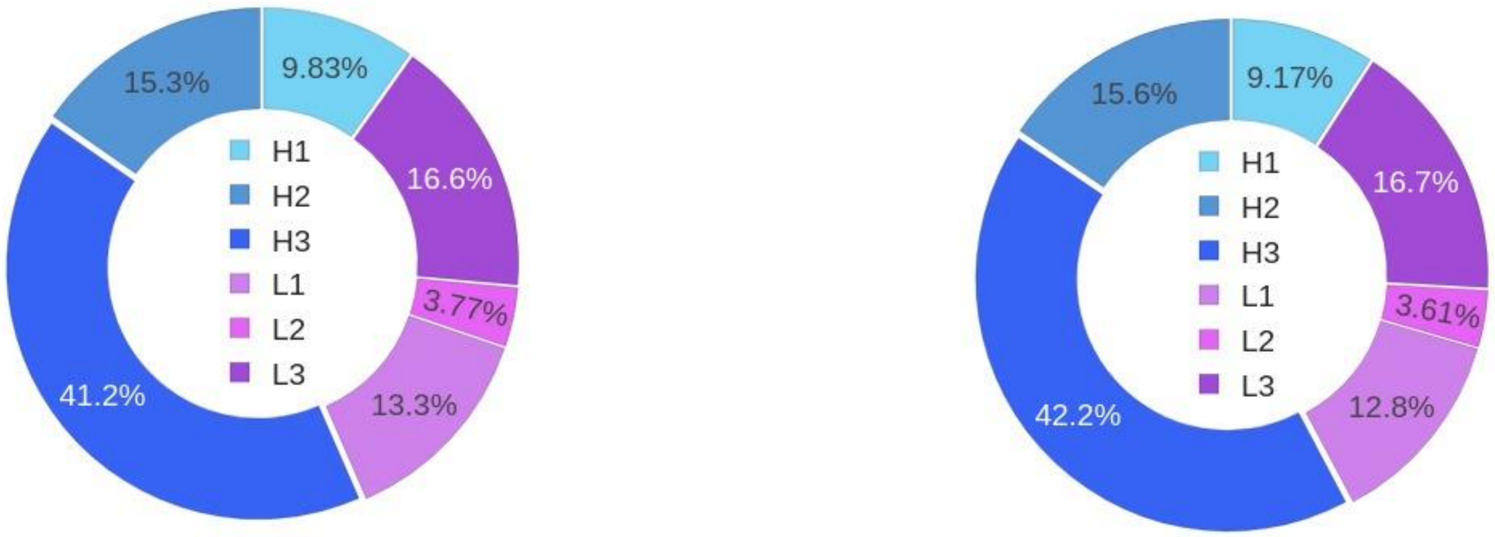
15.3%
9.83%
16.6%
3.77%
13.3%
41.2%
H1
H2
H3
L1
L2
L3
15.6%
9.17%
16.7%
3.61%
12.8%
42.2%
H1
H2
H3
L1
L2
L3

Figure 6D

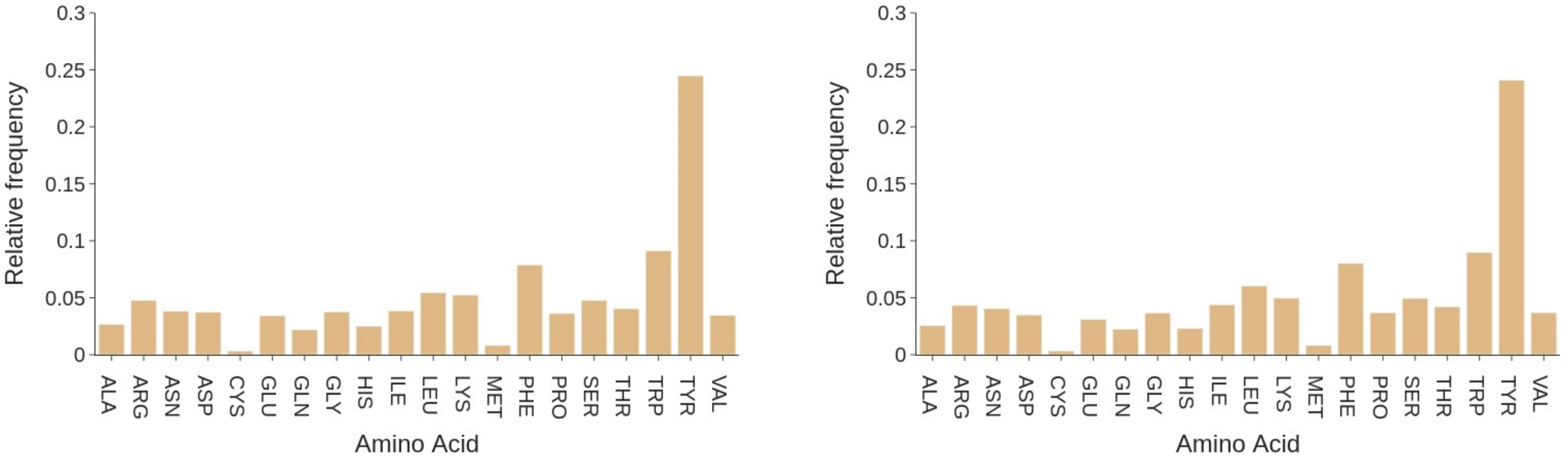
Relative frequency
0.3
0.25
0.2
0.15
0.1
0.05
0
ALA ARG ASN ASP CYS GLU GLN GLY HIS ILE LEU LYS MET PHE PRO SER THR TRP TYR VAL
Amino Acid
Relative frequency
0.3
0.25
0.2
0.15
0.1
0.05
0
ALA ARG ASN ASP CYS GLU GLN GLY HIS ILE LEU LYS MET PHE PRO SER THR TRP TYR VAL
Amino Acid

Figure 8B

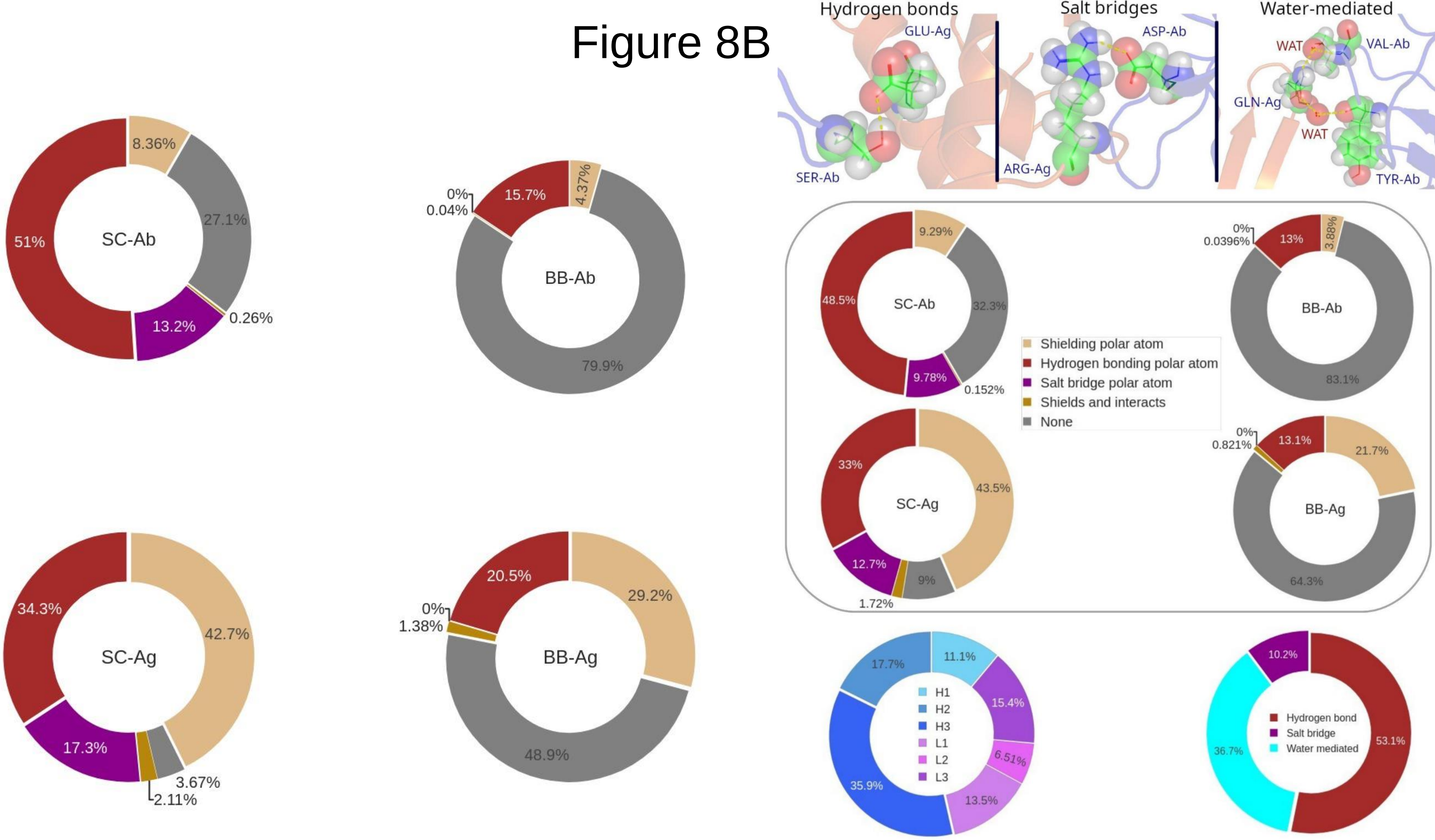

SC-Ab
8.36%
27.1%
0.26%
13.2%
51%
BB-Ab
4.37%
15.7%
0%
0.04%
79.9%
SC-Ag
42.7%
3.67%
2.11%
17.3%
34.3%
BB-Ag
29.2%
48.9%
0%
1.38%
20.5%
Hydrogen bonds
Salt bridges
Water-mediated
GLU-Ag
SER-Ab
ASP-Ab
ARG-Ag
WAT
VAL-Ab
GLN-Ag
WAT
TYR-Ab
SC-Ab
9.29%
32.3%
0.152%
9.78%
48.5%
BB-Ab
3.88%
13%
0%
0.0396%
83.1%
Shielding polar atom
Hydrogen bonding polar atom
Salt bridge polar atom
Shields and interacts
None
SC-Ag
43.5%
9%
1.72%
12.7%
33%
BB-Ag
21.7%
64.3%
0%
0.821%
13.1%
H1
H2
H3
L1
L2
L3
11.1%
15.4%
6.51%
13.5%
35.9%
17.7%
Hydrogen bond
Salt bridge
Water mediated
53.1%
36.7%
10.2%

Figure 8C

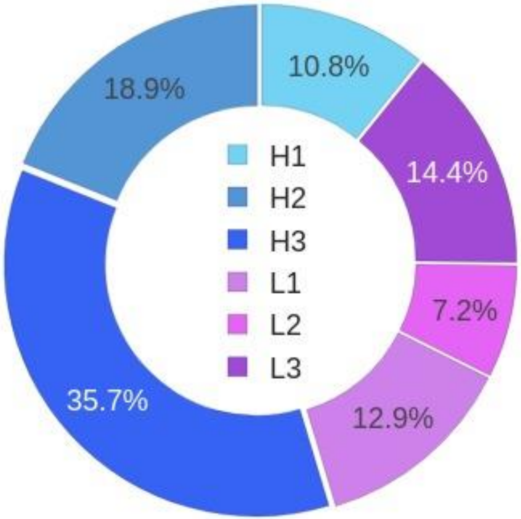
18.9%
10.8%
14.4%
7.2%
12.9%
35.7%
H1
H2
H3
L1
L2
L3

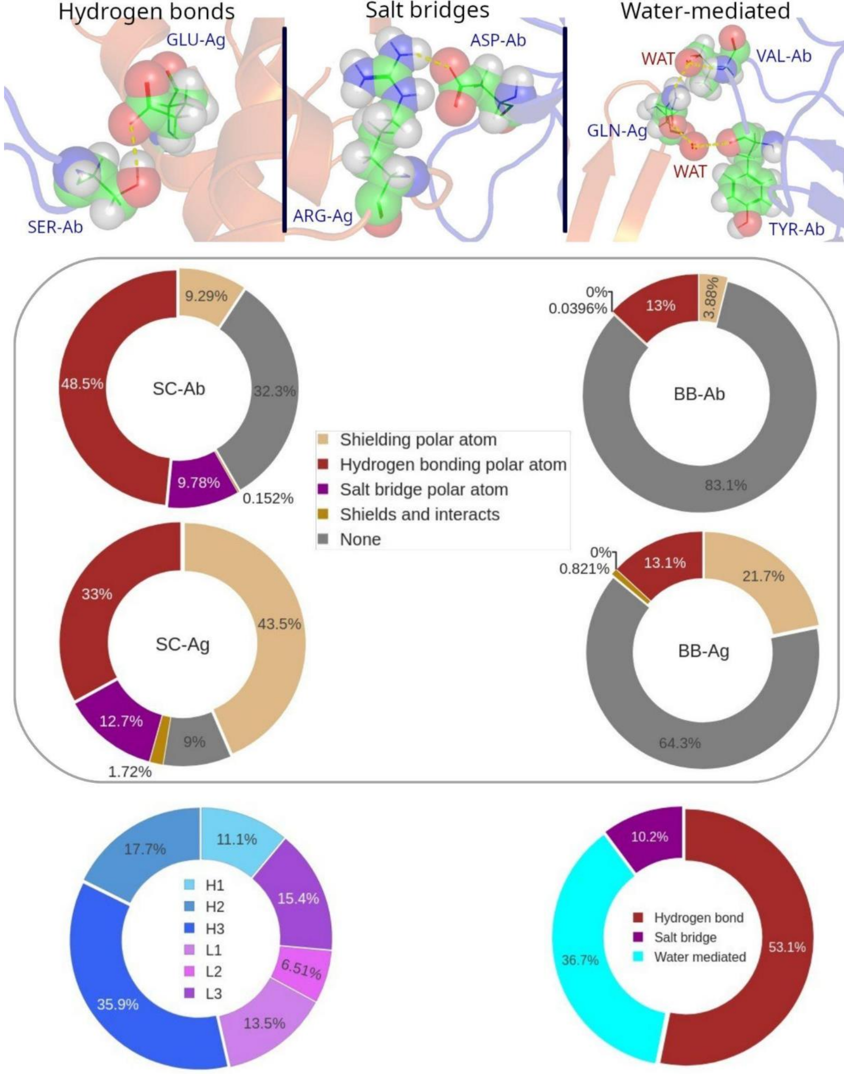
Hydrogen bonds
Salt bridges
Water-mediated
GLU-Ag
SER-Ab
ASP-Ab
ARG-Ag
WAT
VAL-Ab
GLN-Ag
WAT
TYR-Ab
SC-Ab
9.29%
32.3%
0.152%
9.78%
48.5%
BB-Ab
0%
0.0396%
13%
3.88%
83.1%
Shielding polar atom
Hydrogen bonding polar atom
Salt bridge polar atom
Shields and interacts
None
SC-Ag
43.5%
9%
1.72%
12.7%
33%
BB-Ag
0%
0.821%
13.1%
21.7%
64.3%
11.1%
15.4%
6.51%
13.5%
35.9%
17.7%
H1
H2
H3
L1
L2
L3
10.2%
53.1%
36.7%
Hydrogen bond
Salt bridge
Water mediated

Figure 8D

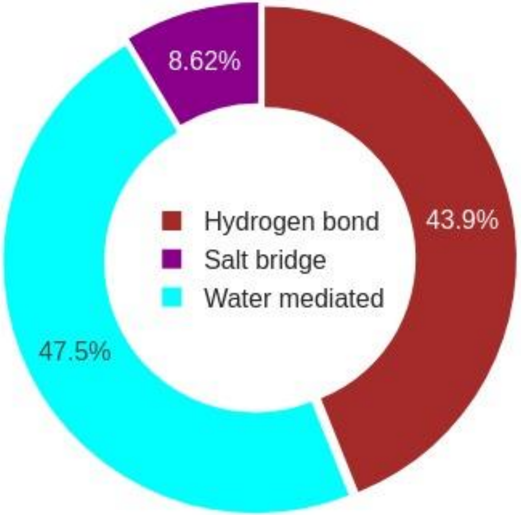
8.62%
Hydrogen bond
Salt bridge
Water mediated
43.9%
47.5%

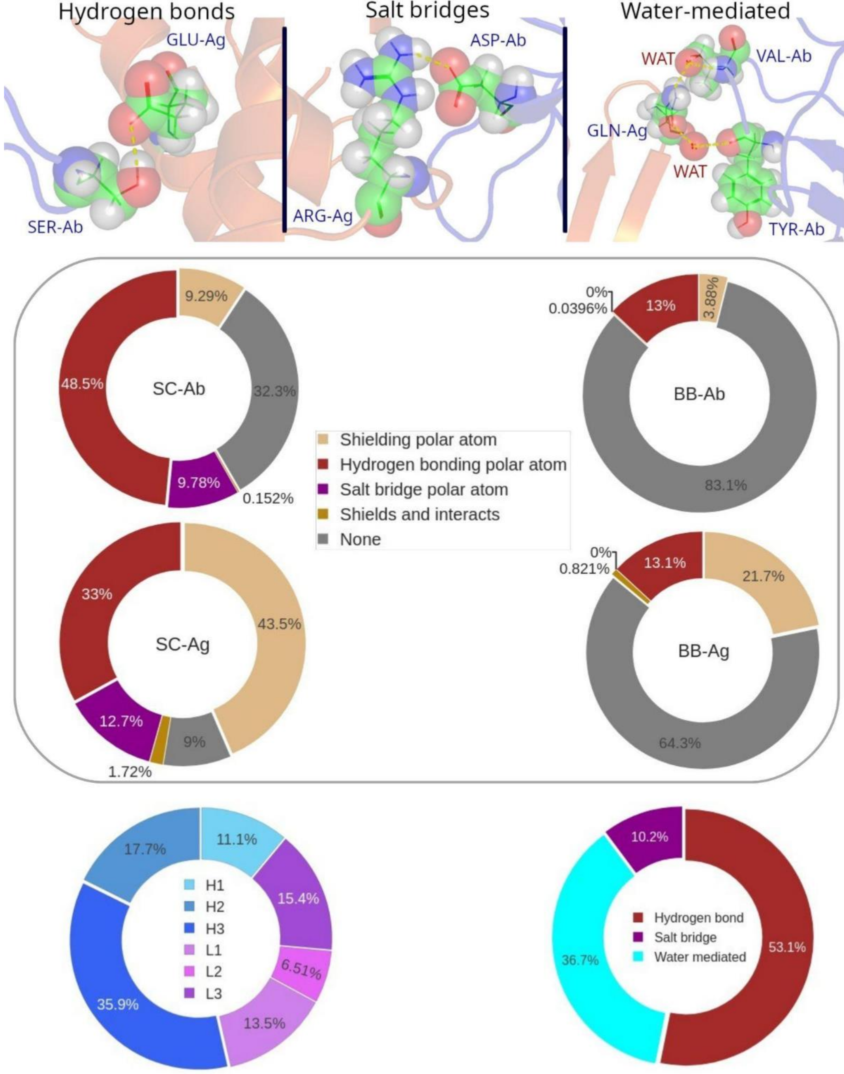
Hydrogen bonds
GLU-Ag
SER-Ab
Salt bridges
ASP-Ab
ARG-Ag
Water-mediated
WAT
VAL-Ab
GLN-Ag
WAT
TYR-Ab
9.29%
48.5%
SC-Ab
32.3%
9.78%
0.152%
0%
0.0396%
13%
3.88%
BB-Ab
83.1%
Shielding polar atom
Hydrogen bonding polar atom
Salt bridge polar atom
Shields and interacts
None
33%
SC-Ag
43.5%
12.7%
9%
1.72%
0%
0.821%
13.1%
21.7%
BB-Ag
64.3%
17.7%
11.1%
H1
H2
H3
L1
L2
L3
15.4%
6.51%
13.5%
35.9%
10.2%
Hydrogen bond
Salt bridge
Water mediated
53.1%
36.7%

Figure 10

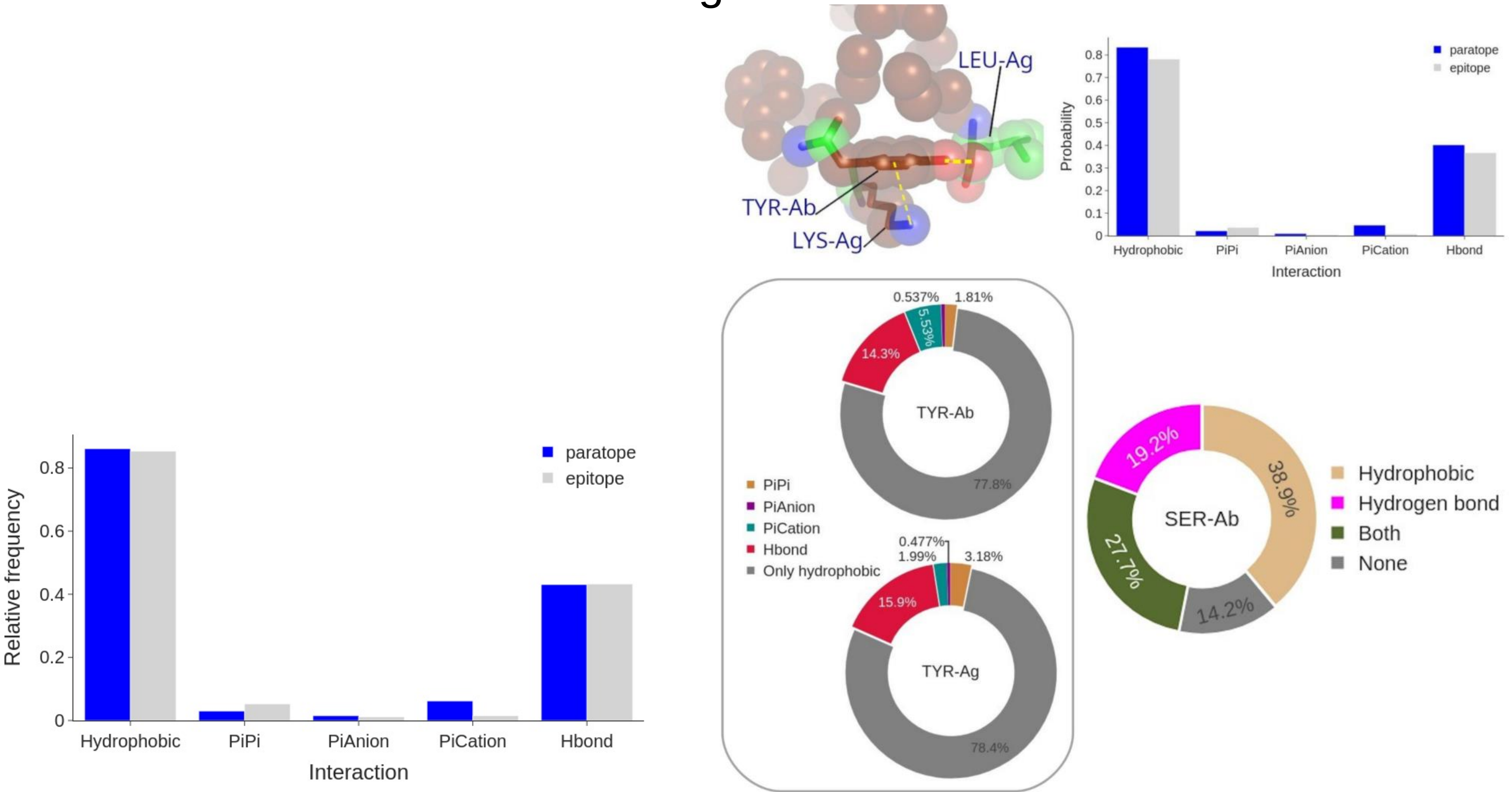

LEU-Ag
TYR-Ab
LYS-Ag
Probability
0.8
0.7
0.6
0.5
0.4
0.3
0.2
0.1
0
paratope
epitope
Hydrophobic
PiPi
PiAnion
PiCation
Hbond
Interaction
0.537%
1.81%
5.53%
14.3%
TYR-Ab
77.8%
PiPi
PiAnion
PiCation
Hbond
Only hydrophobic
0.477%
1.99%
3.18%
15.9%
TYR-Ag
78.4%
19.2%
38.9%
SER-Ab
27.7%
14.2%
Hydrophobic
Hydrogen bond
Both
None
Relative frequency
0.8
0.6
0.4
0.2
0
paratope
epitope
Hydrophobic
PiPi
PiAnion
PiCation
Hbond
Interaction

fig10

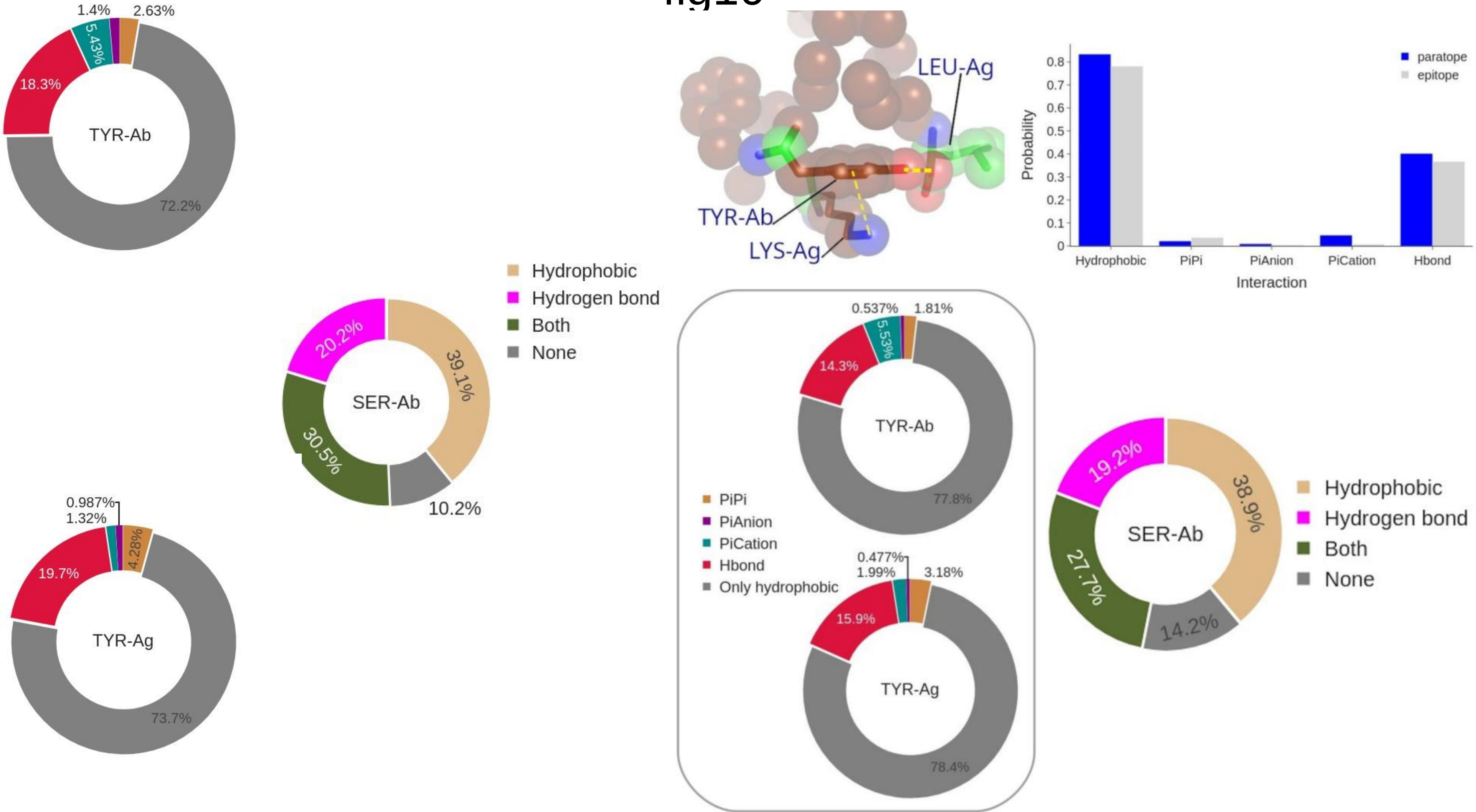
1.4%
2.63%
5.43%
18.3%
TYR-Ab
72.2%
Hydrophobic
Hydrogen bond
Both
None
20.2%
39.1%
SER-Ab
30.5%
10.2%
0.987%
1.32%
4.28%
19.7%
TYR-Ag
73.7%
LEU-Ag
TYR-Ab
LYS-Ag
paratope
epitope
Probability
0.8
0.7
0.6
0.5
0.4
0.3
0.2
0.1
0
Hydrophobic
PiPi
PiAnion
PiCation
Hbond
Interaction
0.537%
1.81%
5.53%
14.3%
TYR-Ab
77.8%
PiPi
PiAnion
PiCation
Hbond
Only hydrophobic
0.477%
1.99%
3.18%
15.9%
TYR-Ag
78.4%
19.2%
38.9%
SER-Ab
27.7%
14.2%
Hydrophobic
Hydrogen bond
Both
None

Figure S5

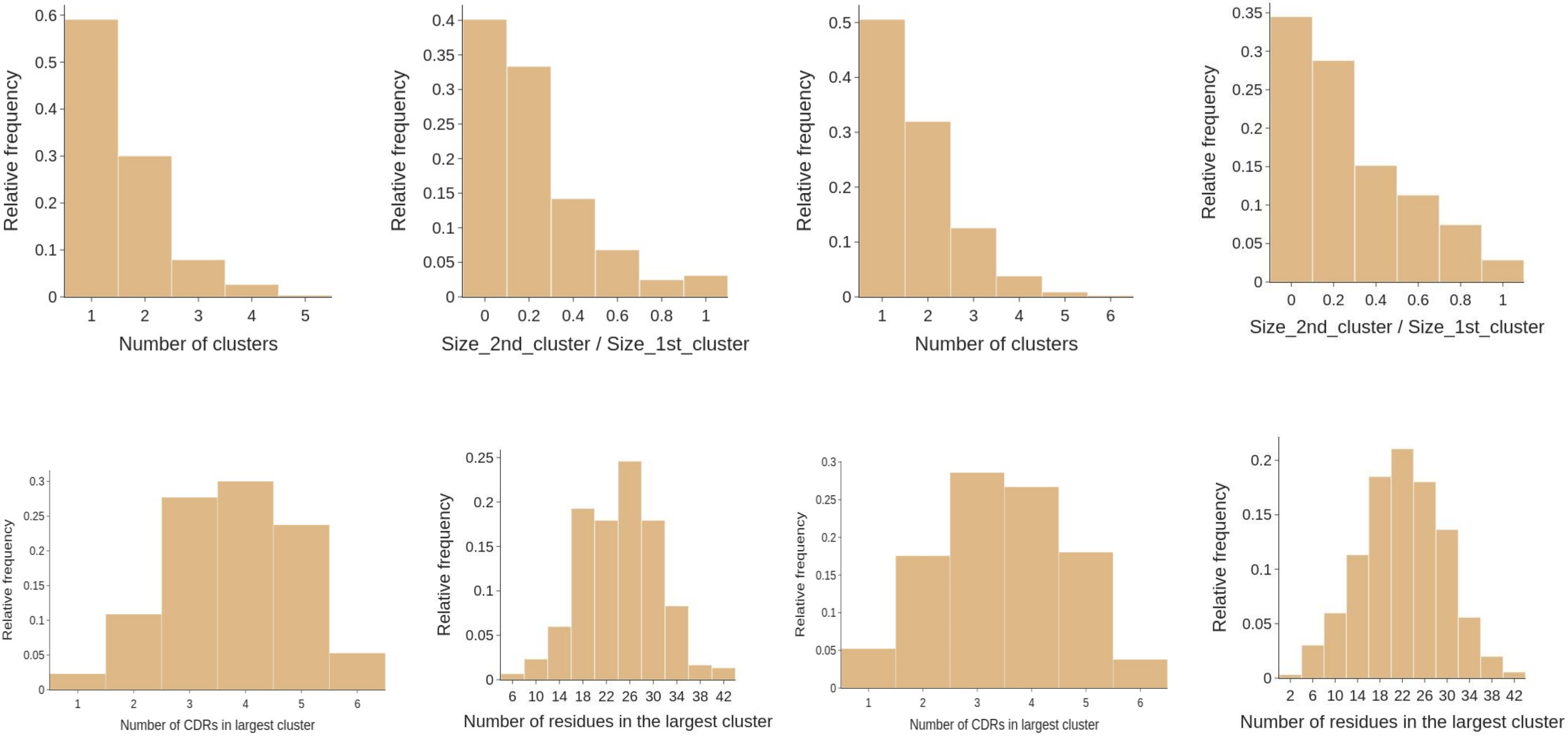

Relative frequency
0.6
0.5
0.4
0.3
0.2
0.1
0
1 2 3 4 5
Number of clusters
Relative frequency
0.4
0.35
0.3
0.25
0.2
0.15
0.1
0.05
0
0 0.2 0.4 0.6 0.8 1
Size_2nd_cluster / Size_1st_cluster
Relative frequency
0.5
0.4
0.3
0.2
0.1
0
1 2 3 4 5 6
Number of clusters
Relative frequency
0.35
0.3
0.25
0.2
0.15
0.1
0.05
0
0 0.2 0.4 0.6 0.8 1
Size_2nd_cluster / Size_1st_cluster
Relative frequency
0.3
0.25
0.2
0.15
0.1
0.05
0
1 2 3 4 5 6
Number of CDRs in largest cluster
Relative frequency
0.25
0.2
0.15
0.1
0.05
0
6 10 14 18 22 26 30 34 38 42
Number of residues in the largest cluster
Relative frequency
0.3
0.25
0.2
0.15
0.1
0.05
0
1 2 3 4 5 6
Number of CDRs in largest cluster
Relative frequency
0.2
0.15
0.1
0.05
0
2 6 10 14 18 22 26 30 34 38 42
Number of residues in the largest cluster

Figure S5

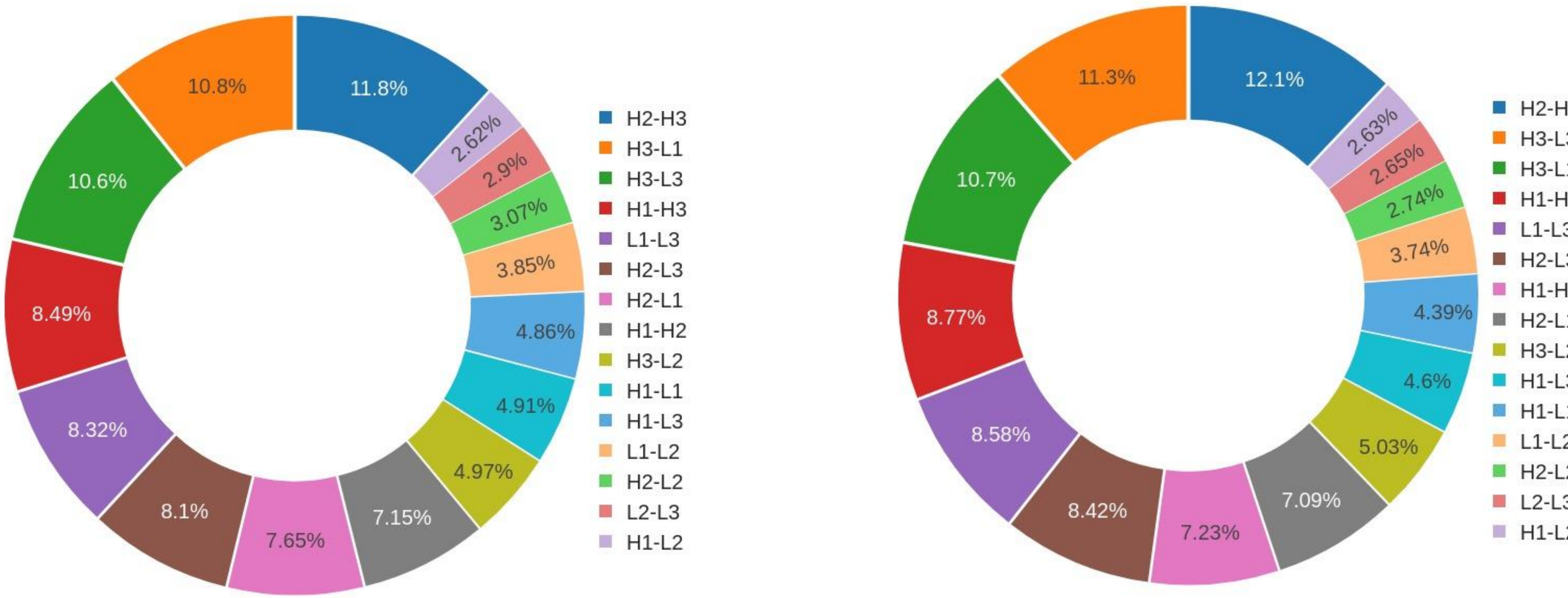

11.8%
10.8%
10.6%
8.49%
8.32%
8.1%
7.65%
7.15%
4.97%
4.91%
4.86%
3.85%
3.07%
2.9%
2.62%
H2-H3
H3-L1
H3-L3
H1-H3
L1-L3
H2-L3
H2-L1
H1-H2
H3-L2
H1-L1
H1-L3
L1-L2
H2-L2
L2-L3
H1-L2
12.1%
11.3%
10.7%
8.77%
8.58%
8.42%
7.23%
7.09%
5.03%
4.6%
4.39%
3.74%
2.74%
2.65%
2.63%

Figure S7

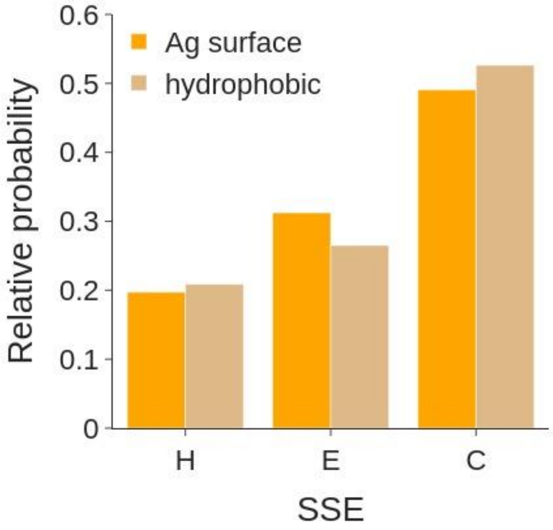
0.6
0.5
0.4
0.3
0.2
0.1
0
Ag surface
hydrophobic
Relative probability
H
E
C
SSE

A)

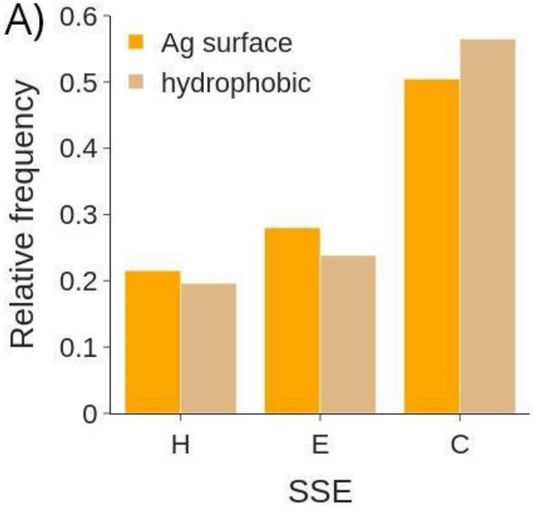
0.6
0.5
0.4
0.3
0.2
0.1
0
Ag surface
hydrophobic
Relative frequency
H
E
C
SSE

B)

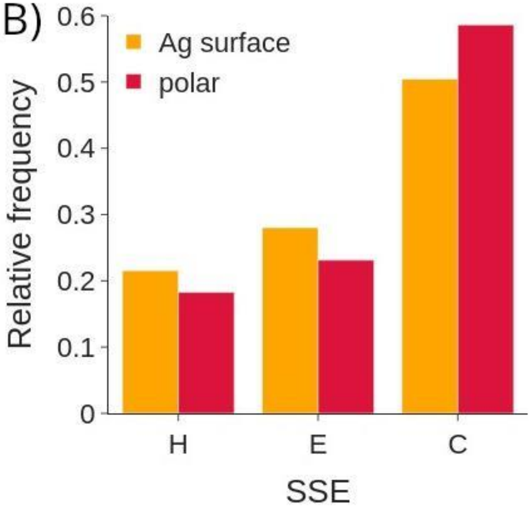
0.6
0.5
0.4
0.3
0.2
0.1
0
Ag surface
polar
Relative frequency
H
E
C
SSE

Figure S7

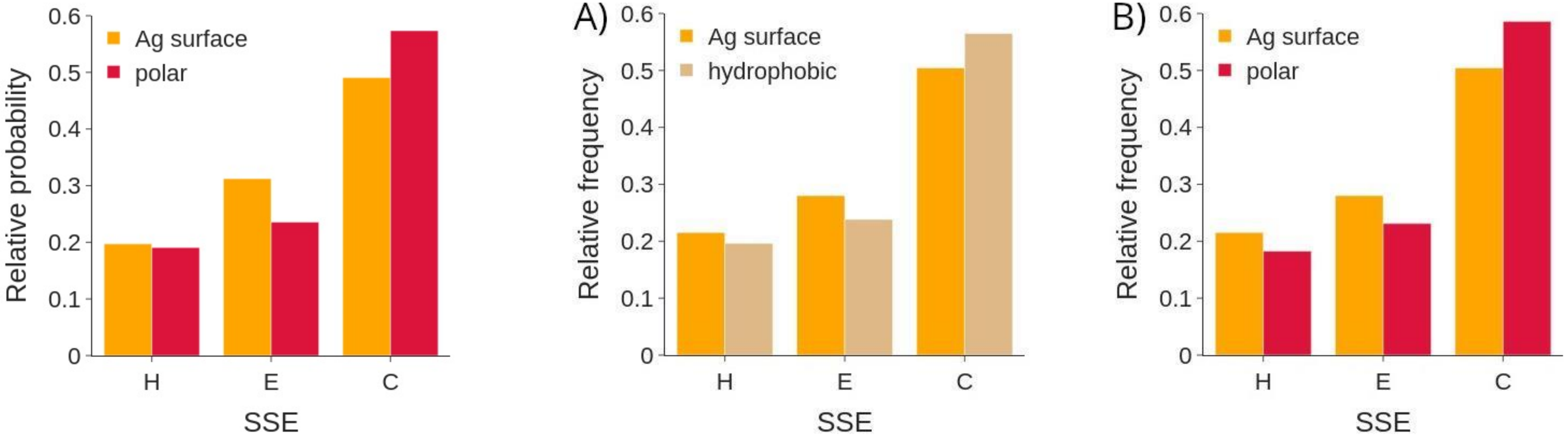
0.6
0.5
0.4
0.3
0.2
0.1
0
Ag surface
polar
Relative probability
H
E
C
SSE
A)
Ag surface
hydrophobic
Relative frequency
H
E
C
SSE
B)
Ag surface
polar
Relative frequency
H
E
C
SSE

Figure S8

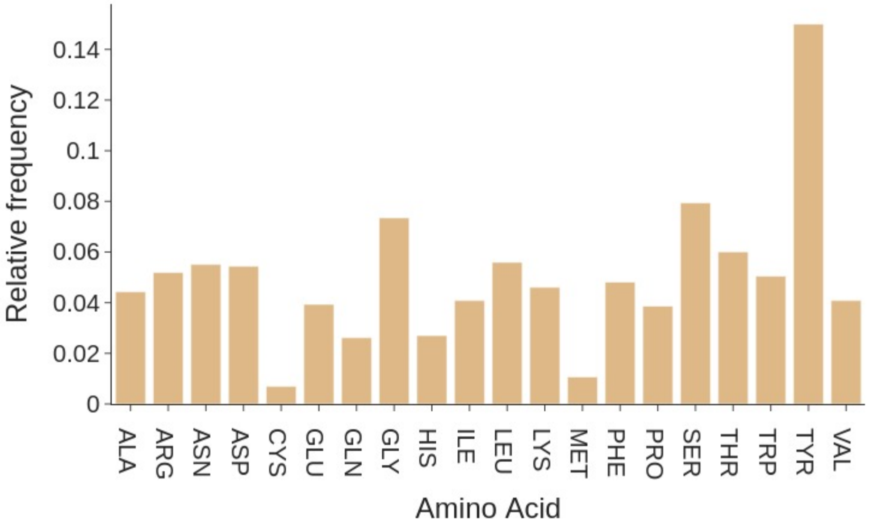

Relative frequency
0.14
0.12
0.1
0.08
0.06
0.04
0.02
0
ALA ARG ASN ASP CYS GLU GLN GLY HIS ILE LEU LYS MET PHE PRO SER THR TRP TYR VAL
Amino Acid

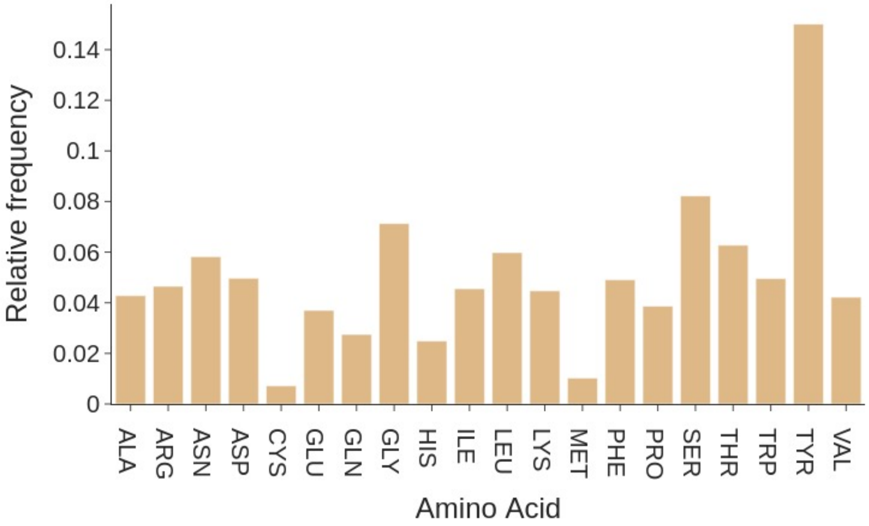

Relative frequency
0.14
0.12
0.1
0.08
0.06
0.04
0.02
0
ALA ARG ASN ASP CYS GLU GLN GLY HIS ILE LEU LYS MET PHE PRO SER THR TRP TYR VAL
Amino Acid

Figure S11

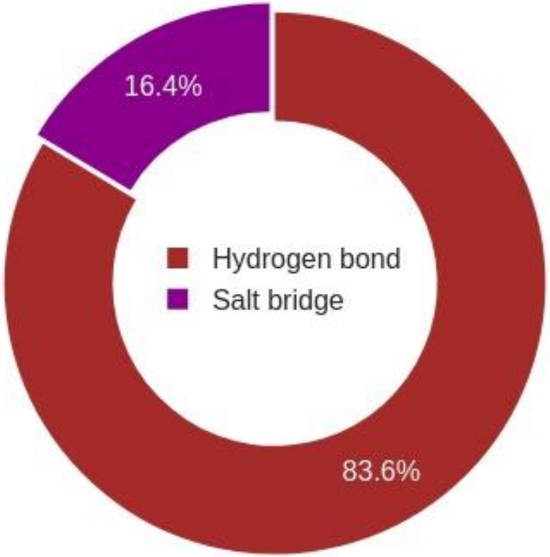
16.4%
Hydrogen bond
Salt bridge
83.6%

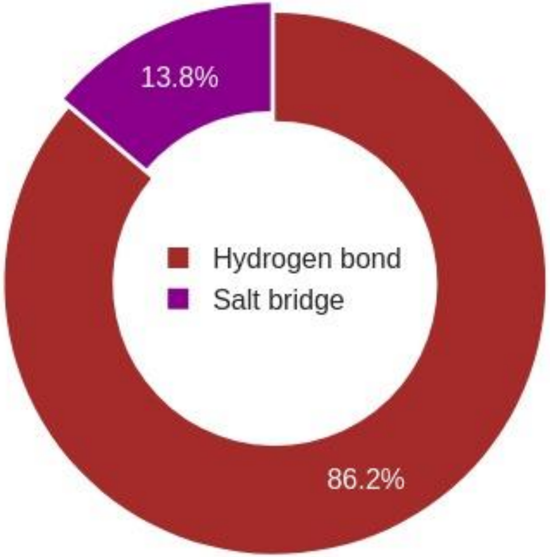
13.8%
Hydrogen bond
Salt bridge
86.2%

Figure S12

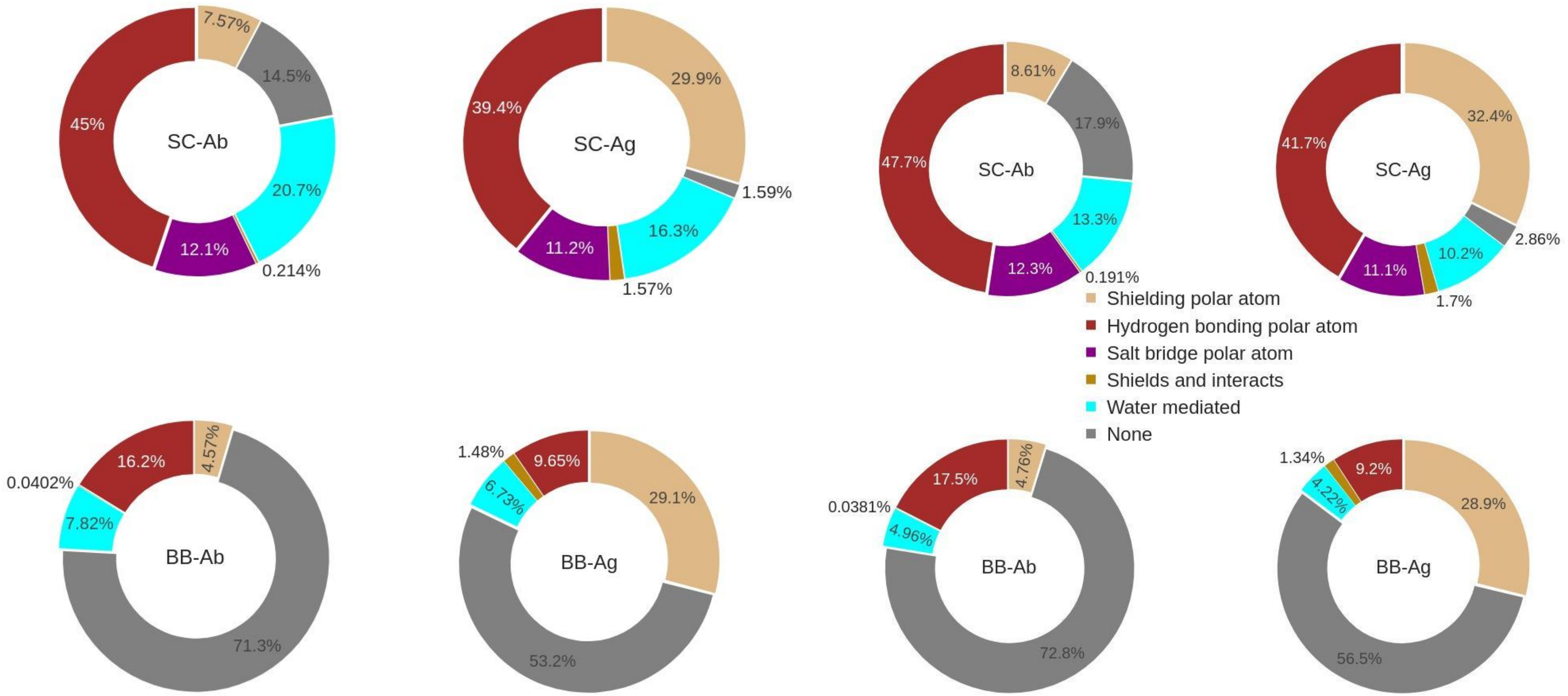
SC-Ab
7.57%
14.5%
45%
20.7%
12.1%
0.214%
SC-Ag
29.9%
39.4%
1.59%
16.3%
11.2%
1.57%
SC-Ab
8.61%
17.9%
47.7%
13.3%
12.3%
0.191%
SC-Ag
32.4%
41.7%
2.86%
10.2%
11.1%
1.7%
Shielding polar atom
Hydrogen bonding polar atom
Salt bridge polar atom
Shields and interacts
Water mediated
None
BB-Ab
16.2%
4.57%
0.0402%
7.82%
71.3%
BB-Ag
1.48%
9.65%
6.73%
29.1%
53.2%
BB-Ab
17.5%
4.76%
0.0381%
4.96%
72.8%
BB-Ag
1.34%
9.2%
4.22%
28.9%
56.5%

Figure S14

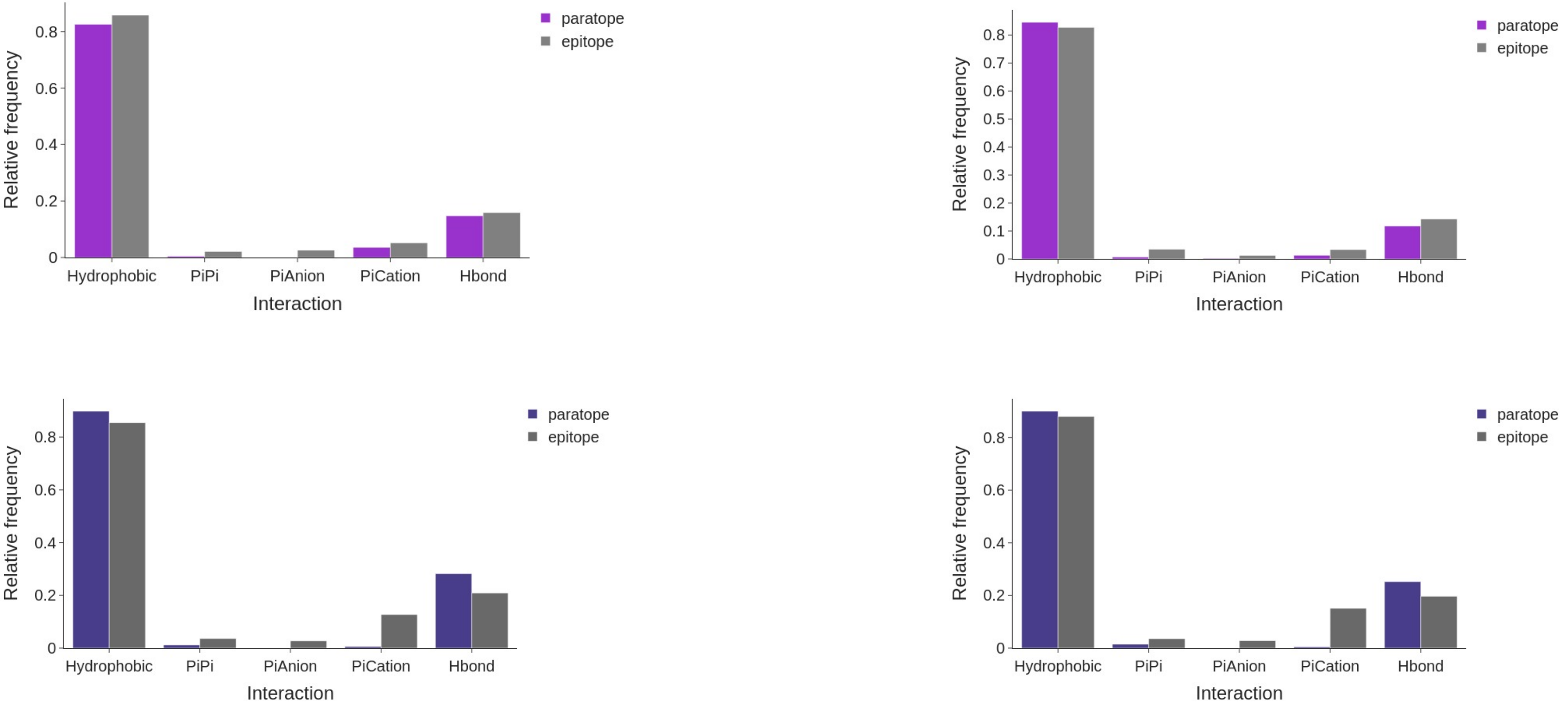

paratope
epitope
Relative frequency
0
0.2
0.4
0.6
0.8
Hydrophobic
PiPi
PiAnion
PiCation
Hbond
Interaction
paratope
epitope
Relative frequency
0
0.1
0.2
0.3
0.4
0.5
0.6
0.7
0.8
Hydrophobic
PiPi
PiAnion
PiCation
Hbond
Interaction
paratope
epitope
Relative frequency
0
0.2
0.4
0.6
0.8
Hydrophobic
PiPi
PiAnion
PiCation
Hbond
Interaction
paratope
epitope
Relative frequency
0
0.2
0.4
0.6
0.8
Hydrophobic
PiPi
PiAnion
PiCation
Hbond
Interaction

Figure S15

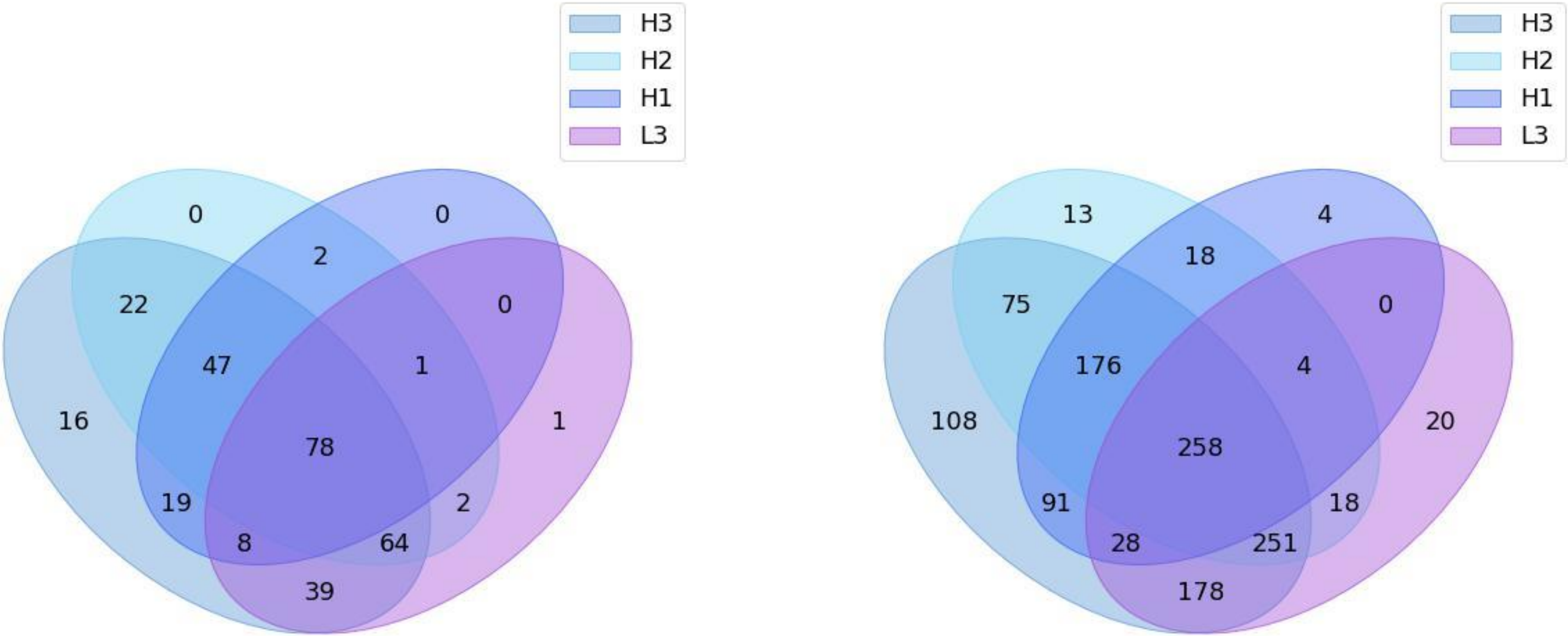
H3
H2
H1
L3
0
0
2
22
0
47
1
16
1
78
19
2
8
64
39
H3
H2
H1
L3
13
4
18
75
0
176
4
108
20
258
91
18
28
251
178

Figure S17

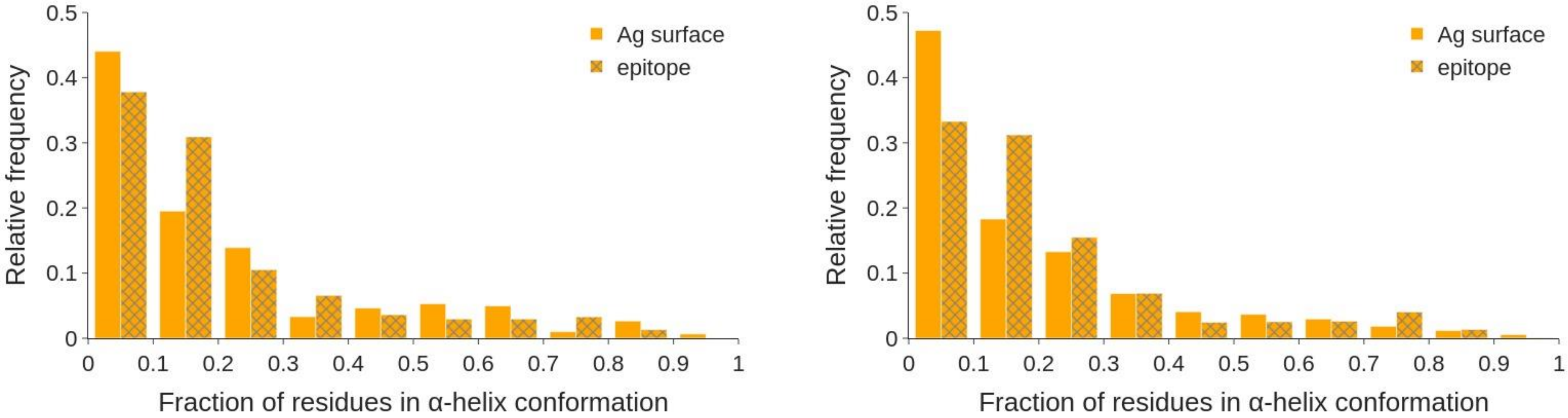
Ag surface
epitope
Relative frequency
Fraction of residues in α-helix conformation
0
0.1
0.2
0.3
0.4
0.5
0.6
0.7
0.8
0.9
1
Ag surface
epitope
Relative frequency
Fraction of residues in α-helix conformation

Figure S18

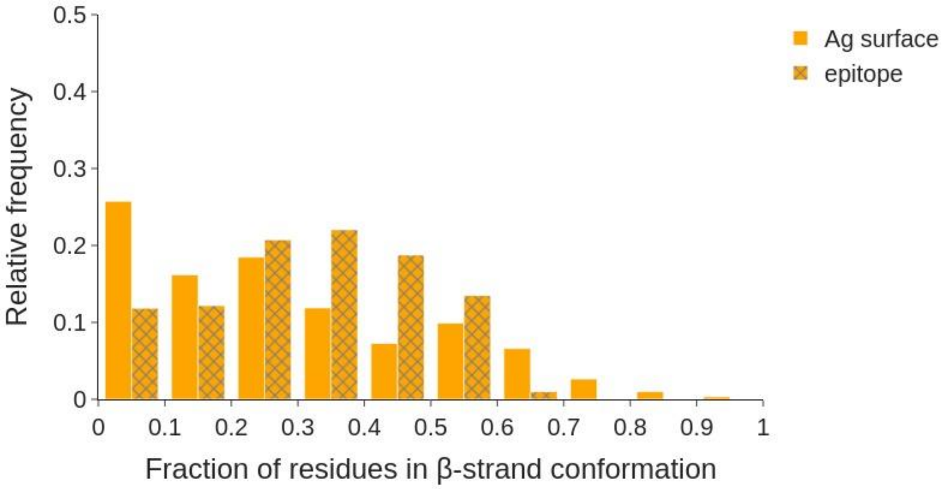

Ag surface
epitope
Relative frequency
Fraction of residues in β-strand conformation
0.5
0.4
0.3
0.2
0.1
0
0 0.1 0.2 0.3 0.4 0.5 0.6 0.7 0.8 0.9 1

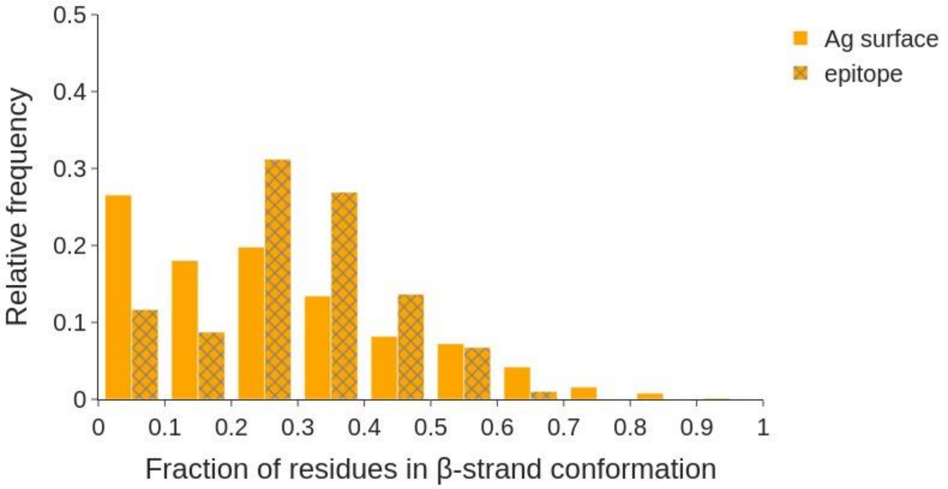

Ag surface
epitope
Relative frequency
Fraction of residues in β-strand conformation
0.5
0.4
0.3
0.2
0.1
0
0 0.1 0.2 0.3 0.4 0.5 0.6 0.7 0.8 0.9 1

Figure S19

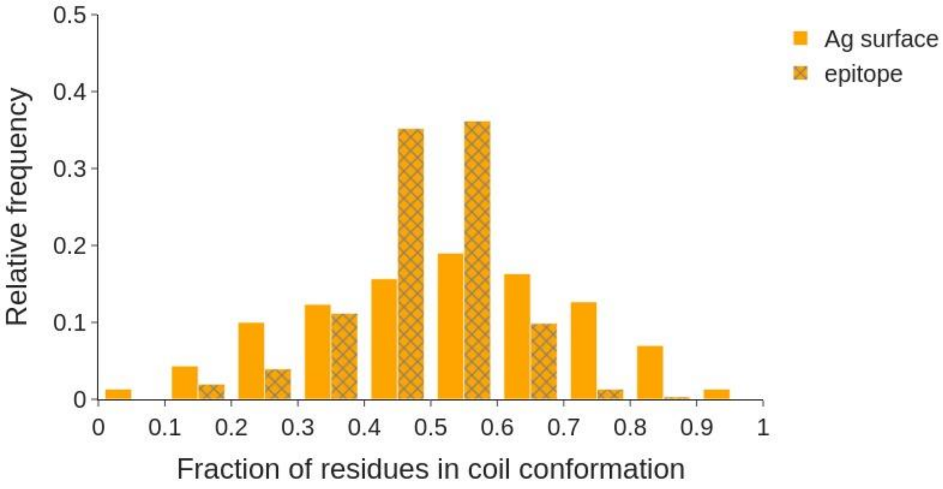

0.5
0.4
0.3
0.2
0.1
0
Relative frequency
Ag surface
epitope
0
0.1
0.2
0.3
0.4
0.5
0.6
0.7
0.8
0.9
1
Fraction of residues in coil conformation

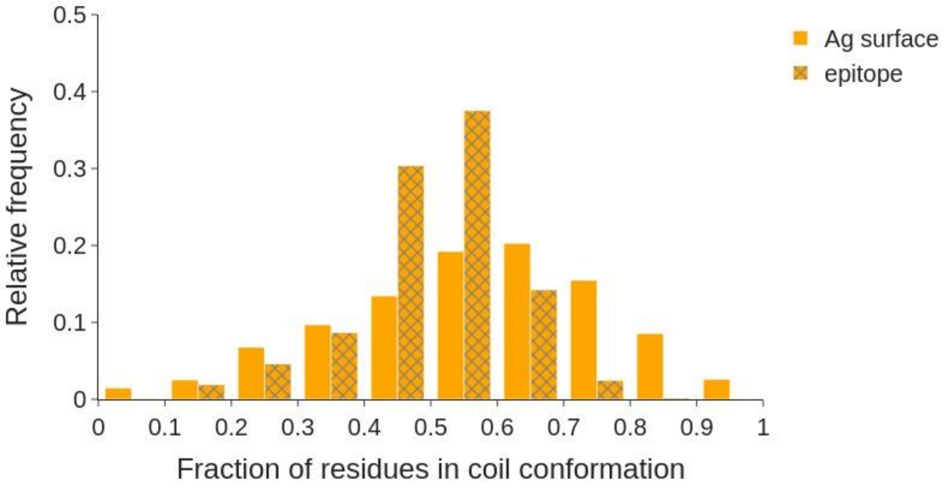

0.5
0.4
0.3
0.2
0.1
0
Relative frequency
Ag surface
epitope
0
0.1
0.2
0.3
0.4
0.5
0.6
0.7
0.8
0.9
1
Fraction of residues in coil conformation
